# Dietary sodium enhances the expression of SLC4 family transporters, IRBIT, L-IRBIT, and PP1 in rat kidney: Insights into the molecular mechanism for renal sodium handling

**DOI:** 10.3389/fphys.2023.1154694

**Published:** 2023-04-04

**Authors:** Lu Cai, Dengke Wang, Tianxiang Gui, Xiaoyu Wang, Lingyu Zhao, Walter F. Boron, Li-Ming Chen, Ying Liu

**Affiliations:** ^1^ Key Laboratory of Molecular Biophysics of Ministry of Education, School of Life Science and Technology, Huazhong University of Science and Technology, Wuhan, Hubei, China; ^2^ Department of Physiology and Biophysics, Case Western Reserve University School of Medicine, Cleveland, OH, United States

**Keywords:** solute carriers (SLC), Na^+^-bicarbonate cotransporter, SLC4A2, SLC4A7, SLC4A10, thick ascending limb of Henle’s loop, Na^+^-Cl^−^cotransporter NCC, renal sodium reabsorption

## Abstract

The kidney plays a central role in maintaining the fluid and electrolyte homeostasis in the body. Bicarbonate transporters NBCn1, NBCn2, and AE2 are expressed at the basolateral membrane of the medullary thick ascending limb (mTAL). In a previous study, NBCn1, NBCn2, and AE2 are proposed to play as a regulatory pathway to decrease NaCl reabsorption in the mTAL under high salt condition. When heterologously expressed, the activity of these transporters could be stimulated by the InsP3R binding protein released with inositol 1,4,5-trisphosphate (IRBIT), L-IRBIT (collectively the IRBITs), or protein phosphatase PP1. In the present study, we characterized by immunofluorescence the expression and localization of the IRBITs, and PP1 in rat kidney. Our data showed that the IRBITs were predominantly expressed from the mTAL through the distal renal tubules. PP1 was predominantly expressed in the TAL, but is also present in high abundance from the distal convoluted tubule through the medullary collecting duct. Western blotting analyses showed that the abundances of NBCn1, NBCn2, and AE2 as well as the IRBITs and PP1 were greatly upregulated in rat kidney by dietary sodium. Co-immunoprecipitation study provided the evidence for protein interaction between NBCn1 and L-IRBIT in rat kidney. Taken together, our data suggest that the IRBITs and PP1 play an important role in sodium handling in the kidney. We propose that the IRBITs and PP1 stimulates NBCn1, NBCn2, and AE2 in the basolateral mTAL to inhibit sodium reabsorption under high sodium condition. Our study provides important insights into understanding the molecular mechanism for the regulation of sodium homeostasis in the body.

## Introduction

The kidney maintains the systemic homeostasis of fluid and electrolytes in the body, and therefore plays a central role in the long-term control of arterial blood pressure (Guyton, 1989; Gonzalez-Vicente et al., 2019). The systemic sodium content is a key factor that determines the volume of blood and extracellular fluid. Sodium retention would increase the blood volume, which in turn would increase the arterial blood pressure. Most of, if not all, the monogenic forms of hypertension are associated with aberrant renal function in sodium handling (Wadei and Textor, 2012).

The thick ascending limb (TAL) reabsorbs ∼25%–30% of the NaCl filtered by the glomerulus (Dimke and Schnermann, 2018; Gonzalez-Vicente et al., 2019). The entry of vast majority of the Na^+^ anc Cl^−^ into the epithelial cells is accounted for by the apical furosemide-sensitive Na^+^-K^+^-Cl^−^ cotransporter NKCC2 (SLC12A1) and the Na^+^-H^+^ exchanger NHE3 (SLC9A3) in the TAL. In the basolateral membrane, Na^+^ is extruded by the Na^+^-K^+^-ATPase, whereas Cl^−^ enters the interstitial fluid *via* the chloride channel CLC-K (for review, see refs. (Bazua-Valenti et al., 2016; Gonzalez-Vicente et al., 2019). Loss-of-function mutations in NKCC2 causes hypotension due to urinary waste of salt, whereas overactivity of NKCC2 results in hypertension (Ji et al., 2008; Ares et al., 2011). In the TAL, NKCC2 is stimulated upon phosphorylation by the SPS/Ste20-related proline-alanine-rich kinase (SPAK), which is phosphorylated by the with-no-Lys kinase WNK (Mutig, 2017; Caceres and Ortiz, 2019). Loss-of-function mutations in WNK and SPAK are associated with hypotension (Richardson and Alessi, 2008; Murthy et al., 2017; Shekarabi et al., 2017).

Genetics studies show that a number of bicarbonate transporters of the solute carrier 4 (SLC4) family are associated with hypertension. These include *SLC4A1* encoding the Cl^−^-HCO_3_^−^ exchanger AE1 (Kokubo et al., 2006), *SLC4A2* encoding AE2 (Sober et al., 2009), *SLC4A4* encoding the Na^+^-HCO_3_^−^ cotransporter NBCe1 (Yang et al., 2012; Guo et al., 2016), *SLC4A5* encoding NBCe2 (Barkley et al., 2004; Carey et al., 2012; Groger et al., 2012), and *SLC4A7* encoding NBCn1 (Ehret et al., 2011; Lu et al., 2015). *SLC4A10* encoding NBCn2 is associated with the regulation of systemic fluid balance (Boger et al., 2017).

The abovementioned SLC4 family members are all expressed in the kidney, making a major contribution to the regulation of systemic acid-base balance in the body. In rat kidney, the activities of Cl^−^-HCO_3_^−^ exchange and Na^+^-dependent HCO_3_^−^ transport are both expressed in the basolateral membrane of the medullary (m) TAL (Kikeri et al., 1990; Bourgeois et al., 2002; Odgaard et al., 2004). In the basolateral mTAL, the Cl^−^-HCO_3_^−^ exchange activity is attributable to the expression of AE2 (Alper et al., 1997), whereas the Na-HCO_3_^−^ cotransport actrivity is attributable to NBCn1 and NBCn2 (Kim et al., 2003; Praetorius et al., 2004; Guo et al., 2017). AE2 mediates the extrusion of HCO_3_^−^ during the transepithelial HCO_3_^−^ reabsorption in the mTAL (Bourgeois et al., 2002). In rat kidney, the abundances of NBCn1, NBCn2, and AE2 are upregulated by overload of NaCl or NaHCO_3_ (Quentin et al., 2004; Wang J. L. et al., 2020). NaHCO_3_ overload induces metabolic alkalosis. As an adaptive response to metabolic alkalosis, the HCO_3_^−^ reabsorption in the mTAL would be expected to be downregulated. The upregulation in AE2 by NaHCO_3_ appears to contradict the role of AE2 in HCO_3_^−^ reabsorption. Similar to NaCl, administration of NaHCO_3_ also causes sodium overload. Thus, it is proposed that, under high sodium condition, the NBCs (NBCn1 and NBCn2) together with AE2 inhibit NaCl reabsorption by counteracting the action of the apical NKCC2 and the basolateral Na^+^-K^+^ ATPase in mTAL (Wang J. L. et al., 2020).

When heterologously expressed in *Xenopus* oocytes, the activities of NBCn1 and NBCn2 are greatly stimulated by protein interaction with the InsP3R binding protein released with inositol 1,4,5-trisphosphate (IRBIT) and L-IRBIT (Wang J. L. et al., 2020). Both IRBIT and L-IRBIT could interact with AE2. The expression and activity of AE2 is stimulated by L-IRBIT, but not IRBIT in human embryonic kidney HEK-293 cells (Itoh et al., 2021). In rat kidney, the expression of IRBIT and L-IRBIT is upregulated under high salt condition (Wang J. L. et al., 2020). In HEK-293 cells, the activity of NBCn1 is downregulated by SPAK and upregulated by protein phosphatase 1 (PP1) (Hong et al., 2013).

Taken together, the above studies suggest the presence of a potential regulatory network involved in the modulation of NaCl reabsorption in the mTAL. However, some major gaps in knowledge remain to be addressed. Are IRBIT, L-IRBIT, and PP1 expressed in the mTAL where NBCn1, NBCn2, and AE2 are localized? Is there an effect of chloride in the upregulation of renal NBCn1, NBCn2, and AE2 by NaCl overload? Would the expression of the bicarbonate transporters and their regulatory partners be affected by varying just the dietary sodium content? In the present study, we investigated the localization of NBCn1, IRBIT, L-IRBIT, and PP1 in rat kidney by immunofluorescence. We also examined the effect of altering dietary sodium on the protein expression of selected transporters and their potential regulators in rat kidney. Our data indicate that the abubndances of these proteins in the kidney are closely correlated with the dietary sodium intake.

## Methods

### Animals and ethical approval

Sprague Dawley (SD) rats were purchased from the Hubei Provincial Center for Disease Control (Wuhan, China) and housed in rodent cages. *Xenopus laevis* clawed frogs were purchased from Shanghai Institute of Biochemistry and Cell Biology, Chinese Academy of Sciences (Shanghai, China) and housed in a fish tank at 18^°^C. All procedures regarding the usage of experimental animals were approved by the Institutional Committee on Animal Care and Use at Huazhong University of Science and Technology.

Rodent chows were purchased from Trophic Animal Feed High-tech Co. Ltd. (Nantong, Jiangsu, China). The standard chow (AIN93M) contained 0.1% Na^+^. The high-sodium chow contained 1%, 2%, or 3% Na^+^. All chows with varying sodium levels were carefully balanced to keep the chloride unchanged in the chow.

To examine the effect of dietary sodium on the kidney, adult SD rats weighing 200∼300 g were randomly assigned into 4 groups. Each group contained at least 8 rats. The control group received standard AIN93 chow containing 0.1% of Na^+^. The other groups received modified chow containing 1%, 2%, or 3% sodium, respectively. All rats had free access to drinking water. After 10 days of treatment, the rats were anesthetized by subcutaneous injection of pentobarbital sodium (2% w/v) and sacrificed for tissue collection. The tissues were immediately frozen in liquid nitrogen upon isolation and stored at −80°C until usage.

### Antibodies

The polyclonal antibody anti-NBCn2 was described previously (Guo et al., 2017). This anti-NBCn2 was directed against the unique Nt of MEIK-NBCn2 (Liu et al., 2013) that is expressed at the basolateral membrane of mTAL (Guo et al., 2017). The polyclonal antibodies anti-NBCn1 and anti-L-IRBIT were described in a previous study (Wang J. L. et al., 2020). Polyclonal rabbit anti-IRBIT was commercially available (catalogue no. 94248S; Cell Signaling Technology, Danvers, MA, USA). Mouse anti-actin was purchased from Beyotime (catalogue no. AA128; Haimen, China). Rabbit anti-AE2 was purchased from ABclonal (catalogue no. A7729; Wuhan, China). Rabbit anti-PP1Cβ (catalogue no. ab53315), mouse anti-*α*1 (catalogue no. AB7671), and mouse anti-calbindin (catalogue no. ab82812) were purchased from Abcam (Shanghai, China). Rabbit anti-pNCC for phosphorylated NCC was purchased from PhosphoSolutions (catalogue no. p1311-53; Aurora, CO, USA). Mouse anti-NKCC2 was purchased from Santa Cruz (catalogue no. sc-293222; Santa Cruz, California, USA).

HRP-conjugated goat anti-rabbit IgG (catalogue no. A0218) and goat anti-mouse IgG (catalogue no. A0216) were purchased from Beyotime (Haining, Jiangsu, China]. DyLight549-conjugated goat anti-rabbit IgG catalogue no. A23320 and DyLight488-conjugated goat anti-mouse IgG (catalogue no. A23210) were purchased from Abbkine Scientific (Wuhan, China).

### Protein preparation and Western blotting

For protein preparation, a rat kidney tissue of about 500 mg was placed in 6 mL of precooled protein isolation buffer (in mM: 7.5 NaH_2_PO_4_, 250 sucrose, 5 EDTA, 5 EGTA, pH 7.0) supplemented with 1% protease inhibitor cocktail (catalog no. P8340; Sigma-Aldrich, St. Louis, MO, USA). The tissue was homogenized for 15 strokes with a Glas-Col Teflon glass homogenizer (Glas-Col, Terre Haute, IN, USA) bathed in ice. The crude homogenate was centrifuged at 3,000*g* for 10 min at 4°C to remove the cell debris. A vial of the resultant supernatant was saved, representing the “total proteins.” The membrane proteins were pelleted from the supernatant by ultra-centrifuged at 100,000×*g* for 60 min at 4°C and resuspended in a buffer containing 20 mM Tris, 5 mM EDTA, 5% SDS, pH 8.0. Protein concentration was determined by using Enhanced BCA Protein Assay Kit (catalog no. P0010; Beyotime). The protein preparations were stored in aliquots at −80°C until usage.

Proteins were separated by sodium dodecyl sulfate polyacrylamide gel electrophoresis (SDS-PAGE) and blotted onto a PVDF membrane for Western blotting. The blot was blocked with 5% milk in 1 × TBST (1 mM Tris, 150 mM NaCl, 0.1% Tween 20, pH 7.4) for 1 h at room temperature (RT), incubated with primary antibody in 1 × TBST containing 1% milk, and then washed five times with 1 × TBST. The blot was then incubated with HRP-conjugated secondary antibody in 1 × TBST containing 1% milk at RT and washed five times with 1×TBST. Chemiluminescence was performed with SuperSignal West Pico PLUS Chemiluminescent Substrate (catalogue no. 3580, Thermo Scientific, Rockford, IL, USA) prior to X-ray film exposure. Densitometry was performed with ImageJ (National Institutes of Health, USA).

### Tissue fixation and immunofluorescence

Adult rats were fixed by transcardial perfusion with 4% paraformaldehyde (PFA) in PBS (in mM: 77.4 Na_2_HPO_4_, 22.6 NaH_2_PO_4_, pH 7.4). For immunofluorescence, cryo-sections of kidney were cut with thickness of 8 µm. The section was dehydrated overnight at 60°C and then rehydrated for 1 h in 1 × TBS (1 mM Tris, 150 mM NaCl, pH 7.4). After five 5-min washes with 1 × TBS, the section was incubated in Improved Citrate Antigen Retrieval Solution (catalogue no. P0083; Beyotime) at 98°C for 20 min. After five 5-min washes with 1 × TBS, the section was then blocked for 1 h with Immunol Staining Blocking Buffer (catalogue no. P0102; Beyotime) at RT, and incubated with primary antibody at 4°C overnight. The section was washed five times with 1 × TBS, incubated with DyLight-conjugated secondary antibody at room temperature for 1 h followed by five washes with 1 × TBS, and then mounted with Antifade Polyvinyl Pyrrolidone Medium (catalogue no. P0131; Beyotime). The mounting medium contains 4,6-Diamidino-2-Phenylindole (DAPI). Images were acquired on a FluoView FV3000 confocal microscope (Olympus, Tokyo, Japan).

### Molecular biology

Construction of vectors was performed by regular molecular cloning approaches. For generation of the constructs for yeast-2-hybrid, the cDNA encoding mouse IRBIT (accession no. NM_145542) was fused in-frame to the DNA-binding domain of the transcription factor GAL4 in pGBKT7. The cDNA encoding the amino-terminal portion (residues 1−115) of NBCn1 (accession no. AKS30237.1) was fused in-frame to the activation domain of GAL4 in pGADT7.

For generation of the constructs for expression in *Xenopus* oocytes, the target cDNA was subcloned into pGH19 which contained the T7 promoter for transcription (Trudeau et al., 1995). The plasmid containing the cDNA encoding NBCe1-B tagged with EGFP at the carboxyl-terminus was described previously (Su et al., 2021). All constructs used for electrophysiology analysis were tagged with EGFP at the carboxyl-termini. The constructs were linearized for the preparation of capped cRNAs by *in vitro* transcription with T7 mMessage mMachine kit (Ambion, Austin, TX, USA).

### Yeast two-hybrid

Yeast two-hybrid (Y2H) was performed with Matchmaker® Gold Yeast Two-Hybrid System (catalog no. PT4084-1, Clontech Takara, Mountain View, CA, USA) to investigate the protein interaction between IRBIT and NBCn1. Vectors pGBKT7 (expressing IRBIT) and pGADT7 (expressing NBCn1 Nt) were transformed into haploid yeast stains Y2HGold and Y187, respectively. Y187 and Y2HGold were mated to form diploid and then cultured on either double-dropout (DDO) medium lacking or quadruple-dropout (QDO) medium containing X-α-gal at 30^°^C for 3–4 days. Protein interaction was judged by the formation of blue colonies.

### Electrophysiology recordings

*Xenopus* oocytes were used as expression system for electrophysiology recordings. For oocyte isolation, a female *Xenopus laevis* was anesthetized by immersion in 0.2% ethyl 3-aminobenzoate methanesulfonate or tricaine (catalog no. A5040, Sigma-Aldrich, St. Louis, MO, USA). An ovary lobe was isolated and digested with 2 mg/mL collagenase Type 1A (Sigma-Aldrich, St. Louis, MO, USA) in Ca^2+^-free NRS solution (in mM: 82 NaCl, 2 KCl, 20 MgCl_2_, 5 HEPES, pH 7.50) for 90 min at room temperature. Stage V−VI oocytes were selected for usage. cRNA injection into oocytes was performed with Nanoject II Auto-Nanoliter Injector (Drummond Scientific Company, Broomall, PA, USA). For protein expression, the oocytes injected with cRNA were incubated at 18^°^C for 4–5 days in modified Gibco^TM^ Leibovitz’s L-15 Medium (catalog no. 41300-039, Thermo Fisher Scientific, Waltham, MA, USA) containing 100 units/mL of Gibco^TM^ penicillin and 100 μg/mL of streptomycin (Thermo Fisher Scientific,) with 200 mOsm osmolarity.

Two-electrode voltage clamp were performed with an OC-725C oocyte clamp (Warner Instruments, Hamden, CT, USA) controlled by pCLAMP10.2 (Molecular Devices, San Jose, CA, USA). For voltage-clamp recordings, an oocyte was placed in nominally “HCO_3_^−^-free” ND96 (in mM: 96 NaCl, 2 KCl, 1 MgCl2, 1.8 CaCl2, 5 HEPES, pH 7.5, 200 mOsm) in a chamber and impaled with two microelectrodes filled with 3 M KCl, one for sensing membrane potential (*Vm*) and the other for measuring current. The cell was equilibrated in ND96 until the *Vm* reached stable. The activity of NBCe1 was determined as previously described (Su et al., 2021). Briefly, the current-voltage (I-V) relationships were first acquired for ND96 solution and then for a CO_2_/HCO_3_^−^ solution (in mM: 63 NaCl, 2 KCl, 1 MgCl_2_, 1.8 CaCl_2_, 5 HEPES, 33 NaHCO_3_ bubbled with 5% CO_2_, pH 7.50). The difference between the current in ND96 and that in CO_2_/HCO_3_^−^ represented the net HCO_3_^−^-dependent current mediated by NBCe1. The slope conductance (*G*_*NBC*_) of this net I-V curve between ±40 mV was an index for NBCe1 activity.

### Co-immunoprecipitation

For co-immunoprecipitation, 2.5 mg of membrane proteins of rat kidney were mixture with 1 μL of antibody against L-IRBIT in 1.2 mL of IP buffer (in mM: 20 Tris, 150 NaCl, 2 EDTA, pH 7.5) containing 1% (v/v) Triton X-100 and 1% (v/v) protease inhibitor cocktail (Sigma-Aldrich) and incubated at 4 °C overnight. The mixture was then added with Dynabeads^TM^ M-280 Sheep anti-Rabbit IgG (catalog no. 11204D, Thermo Fisher Scientific) and incubated for 2 h at room temperature. After 3× washes with the IP buffer, the beads were resuspended in 60 μL of SDS sample buffer and incubated at 98°C for 10 min. The supernatant was collected for Western blotting analysis.

### Data analysis

Statistical analyses were performed with GraphPad Prism (GraphPad Software, Inc. URL: www.graphpad.com).

## Results

### NBCn1 is expressed in mTAL, but not in cTAL of rat kidney

**FIGURE 1:**
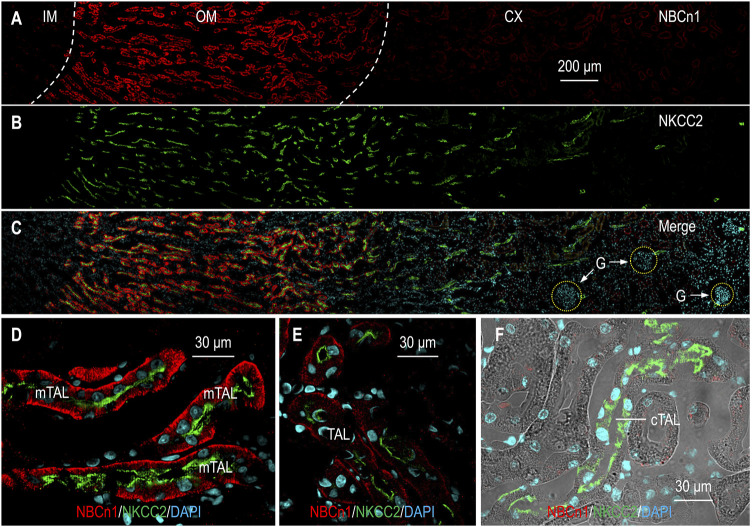
Indirect immunofluorescence of NBCn1 in rat kidney. **(A–C)** Overview of the double staining of NBCn1 and NKCC2 in a rat kidney section. To provide a whole view of the tissue distribution of NBCn1 (red) and NKCC2 (green), the figure was generated by overlaying four continuous images acquired from the inner medulla through the cortex. NKCC2 was present along the whole TAL from the OM through the CX. The fluorescence signal of NBCn1 was strong in the OM, relatively weak in the OM-CX boundary region, and negligible in the CX. **(D)** NBCn1 was highly expressed in the basolateral domain of mTAL. **(E)** NBCn1 was expressed in relatively lower level in the basolateral domain of the TAL in the OM-CX boundary region. **(F)** NBCn1 was not detectable in the cortical TAL. IM, inner medulla; OM, outer medulla; CX, cortex; G, glomerulus. The blue indicates nuclei counter-stained by DAPI.

It has been established that NBCn1 is expressed in the basolateral membrane of mTAL (Praetorius et al., 2004; Wang J. L. et al., 2020). What remains uncertain is whether NBCn1 is expressed in tubular segments beyond the mTAL. In a previous study, we showed by Western blotting that NBCn1 expression is largely limited to the outer medulla in rat kidney (Wang J. L. et al., 2020). A comprehensive RNA-seq study showed that, in addition to mTAL, *Slc4a7* transcripts are also expressed in the descending (DTL), ascending thin limb (ATL) of the Henle’s loop, and cTAL in mouse kidney (Chen et al., 2021; URL: esbl.nhlbi.nih.gov/MRECA/Nephron/). In the present study, we examined the tissue and cellular distribution of NBCn1 in rat kidney by immunofluorescence. We used NKCC2 as a specific marker for the TAL (Ecelbarger et al., 1996; Gimenez and Forbush, 2003; Bazua-Valenti et al., 2016). Figures 1A−C show the overview of double staining of anti-NBCn1 and anti-NKCC2 in a kidney section. The fluorescence signals for NBCn1 were highly enriched in the outer medulla, but not present in the cortex or the inner medulla. High magnification view showed that the expression of NBCn1 was highly abundant in most of the mTAL (Figure 1D) but became much lower in the TAL segments approaching the cortex (Figure 1E), indicating expression heterogeneity in the mTAL. Figure 1F shows the absence of NBCn1 staining in the cTAL. The present immunofluorescence data are consistent with our previous Western blotting data (Wang J. L. et al., 2020), but are somewhat inconsistent with the RNA-seq data obtained from mouse kidney (Chen et al., 2021). The inconsistency could be due to the application of different technical approaches. It could also reflect the species specificity in the expression of *SLC4A7* in the kidney.

### Expression and localization of IRBIT in rat kidney

**FIGURE 2:**
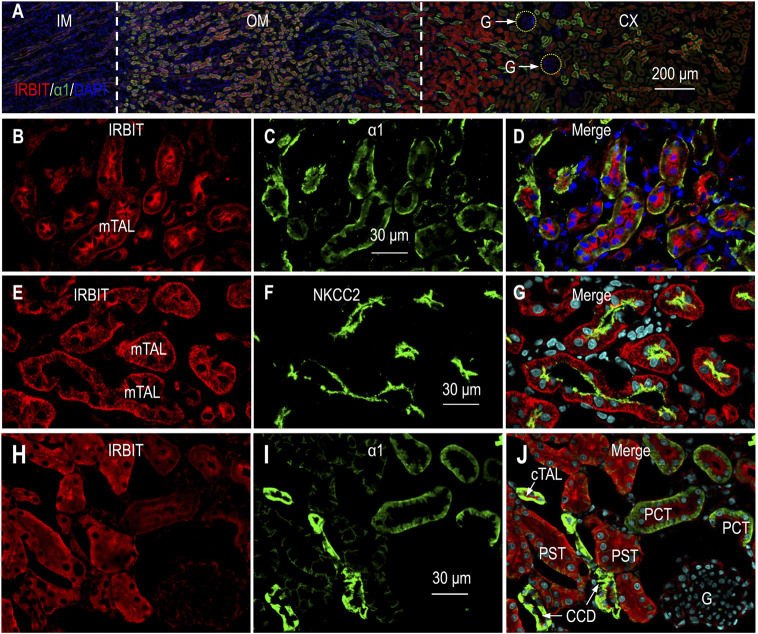
Indirect immunofluorescence of IRBIT in rat kidney. **(A)** Merged overview of double staining of IRBIT (red) and α1 (green) in a rat kidney section. The original unmerged images of IRBIT and α1 were shown in Supplementary Figure S1. **(B–D)** High magnification view showing that IRBIT is abundantly expressed in mTAL. **(E–G)** Localization of IRBIT and NKCC2 in mTAL. **(H–J)** Localization of IRBIT (red) and α1 (green) in cortex. G, glomerulus; mTAL, medullary thick ascending limb; CX, cortex; OM, outer medulla; IM, inner medulla. Blue indicates the nuclei counter-stained with DAPI.

The α1 subunit of the Na^+^-K^+^-ATPase is expressed in virtually all segments along the nephron, with the highest abundance from the mTAL through the cortical connecting tubule (CNT) (Wetzel and Sweadner, 2001; Bankir et al., 2020). We utilized α1 as a marker to determine the localization of IRBIT in rat kidney. Figure 2A shows the overview of the double staining of anti-IRBIT and anti-α1 in a kidney section (also see Supplementary Figure S1). IRBIT was predominantly expressed in the outer medulla and the inner portion of the cortex. The fluorescence signals of IRBIT were also present in specific structures in the outer portion of the cortex, but very weak in the inner medulla.

High magnification view showed that the expression of IRBIT was highly enriched in the mTAL that was intensively labeled by α1 (Figures 2B−D). A double-staining with IRBIT and NKCC2 confirmed the expression of IRBIT in the mTAL (Figures 2E−G).

Figures 2H–J shows the double-staining of IRBIT and α1 in the cortex. Here, strong immunofluorescence signals of IRBIT were observed in the tubules intensively labeled by anti-α1, indicating IRBIT expression in the cTAL through the cortical collecting duct (CCD). The expression of IRBIT in the cTAL was confirmed by double-staining with anti-IRBIT and anti-NKCC2 (data not shown). In the cortex, IRBIT was also abundantly expressed in the proximal tubules. The abundance of IRBIT appeared higher in the PST and the CCD than it was in the proximal convoluted tubules (PCT). Note that, the abundance of α1 was highest in the CCD, lower in the PCT, and even lower in the PST. The fluorescence signal of IRBIT was very faint in the glomeruli.

We further characterized the localization of IRBIT in the cortex by co-staining with calbindin. The Ca^2+^-binding-protein calbindin is a specific marker for the distal convoluted tubules (DCT) through the CCD, with highest abundance in the CNT, and lower in the DCT and CCD (Campean et al., 2001; Wetzel and Sweadner, 2001). Supplementary Figures S2A−C illustrates the representative staining of IRBIT and calbindin in renal cortex. IRBIT was highly enriched in the tubules positive with calbindin. In Supplementary Figures S2D−F, the tubules with the highest expression of calbindin were presumably the CNT, whereas the structures with relatively lower level of calbindin were presumably the CCD. Taken together, the calbindin data show that IRBIT was abundantly expressed in the DCT through the CNT in the renal cortex.

Supplementary Figures S3A−D shows the double-staining of IRBIT and NKCC2 in the cortex of the kidney. Here, IRBIT was expressed in the NKCC2-positive cTAL reaching the macula densa, a specialized tubule juxtaglomerular that plays a critical role for sensing the NaCl content in the tubular fluid (Peti-Peterdi and Harris, 2010). The tubules with IRBIT expression but lacking NKCC2 were presumably the CNT. Supplementary Figures S3E−H shows the double-staining for IRBIT and calbindin around a glomerulus. Here, the juxtaglomerular structure strongly labeled by IRBIT but lacking calbindin was presumably the cTAL, whereas those with both IRBIT and calbindin were presumably the CNTs.

### Expression and localization of L-IRBIT in rat kidney

**FIGURE 3:**
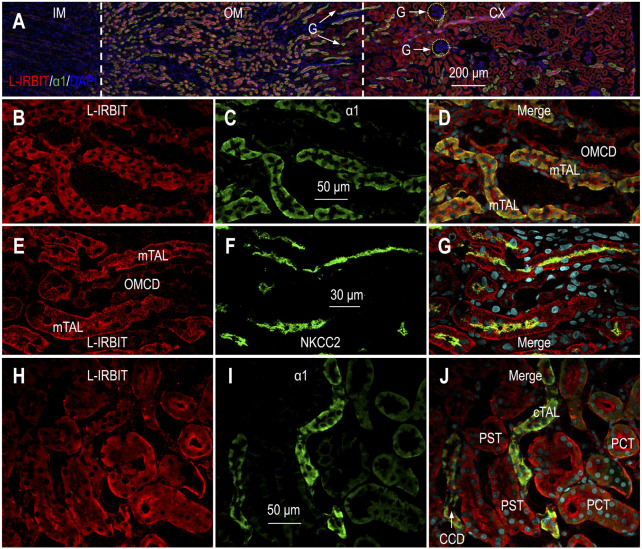
Indirect immunofluorescence of L-IRBIT in rat kidney. **(A)** Merged overview of the double-staining of L-IRBIT (red) and α1 (green). The original unmerged images of L-IRBIT and α1 were shown in Supplementary Figure S4. **(B–D)** Localization of L-IRBIT and α1 of Na^+^ -K^+^ -ATPase in outer medulla. **(E–G)** Localization of L-IRBIT and NKCC2 in outer medulla. **(H–J)** Localization of L-IRBIT (red) and α1 (green) in cortex. CX, cortex; IM, inner medulla; OM, outer medulla; mTAL, medullary thick ascending limb; OMCD, outer medullary collecting duct. Blue indicates the nuclei counter-stained by DAPI.

Figure 3A shows the overview of the double-staining of L-IRBIT and α1 in a kidney section (also see Supplementary Figure S4). Our data show that L-IRBIT was widely expressed from the outer medulla throughout the cortex. Similar to the case of IRBIT, the immunofluorescence signals of L-IRBIT were weak in the inner medulla.

Figures 3B–D represent the high magnification view of L-IRBIT staining in the outer medulla. Here, L-IRBIT was highly enriched in the mTAL intensively labeled by α1. L-IRBIT expression was also observed in relatively lower abundance in the outer medullary collecting duct (OMCD) that was only slightly labeled by α1. Figures 3E−G show the double-staining of L-IRBIT and NKCC2 in the outer medulla. Here, strong signals of L-IRBIT were observed in the mTAL with NKCC2 at the apical domain, as well as in the OMCD lacking NKCC2.

In the cortex, L-IRBIT was ubiquitously expressed in high abundance in the PST with the lowest level of α1, the PCT modestly labeled by α1, and the cTAL and the CCD with the highest level of α1 (Figures 3H−J). Supplementary Figures S5A−C shows the double-staining for L-IRBIT and calbindin in the cortex. The abundance of L-IRBIT was slightly higher in the tubules with calbindin than those without calbindin. Supplementary Figures S5D−F shows the expression of L-IRBIT in NKCC2-positive cTAL.

Taking together, we conclude that L-IRBIT was predominantly expressed from the TAL through the CCD, and was also expressed in relatively lower level in the PCT and the PST in rat kidney.

### Expression and localization of PP1 in rat kidney

**FIGURE 4:**
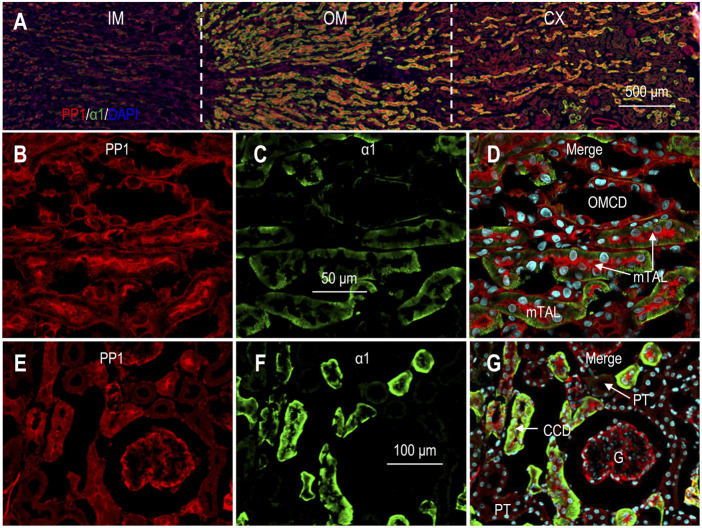
Indirect immunofluorescence of PP1 in rat kidney. **(A)** Merged overview of the double-staining of PP1 and α1 in rat kidney. The original unmerged images of PP1 and α1 were shown in Supplementary Figure S6. **(B–D)** Expression of PP1 and α1 in out medulla. Strong fluorescence signals of PP1 were observed in the mTAL and the OMCD. **(E–G)** Expression of PP1 and α1 in cortex. Fluorescence signals of PP1 were observed in the glomerulus and the cortical collecting duct. G, glomerulus; CCD, cortical collecting duct; mTAL, medullary thick ascending limb; OMCD, outer medullary collecting duct.

Figure 4A shows the overview of the double staining of anti-PP1 and anti-α1 in a kidney section (also see Supplementary Figure S6). The inner medulla was slightly labeled by anti-PP1. In the outer medulla and the cortex, the immunofluorescence signals of PP1 were most intensive in the tubules with the highest levels of α1, suggesting that PP1 expression was enriched from the mTAL through the CCD. Figures 4B−D shows PP1 staining in the outer medulla in high magnification view. Here, PP1 was most abundantly expressed in the mTAL with high levels of α1. PP1 was also expressed in high abundance in the OMCD, the basolateral domain of which was slightly labeled by anti-α1.

In the cortex, strong signals of PP1 were observed in the tubules intensively labeled by α1, suggesting that PP1 was highly expressed from the cTAL through the CCD (Figures 4E−G). In the cortex, PP1 signals were always seen in the tubules positive with calbindin, confirming the expression of PP1 in the DCT, CNT, and CCD (Supplementary Figures S7A−C). Taking together, we conclude that PP1 was predominantly expressed from cTAL through CCD in renal cortex.

Finally, the expression of PP1 was also present in the glomeruli (Figures 4E−G; Supplementary Figure S7).

### Effect of dietary sodium on expression of transporters in rat kidney

We examined the effect of dietary sodium on the expression of selected transporters in rat kidney. Adult rats were treated for 10 days either with standard rodent chow containing 0.1% Na^+^ or modified rodent chow containing high sodium. The membrane preparations of whole kidneys were used for Western blotting analyses.

**FIGURE 5:**
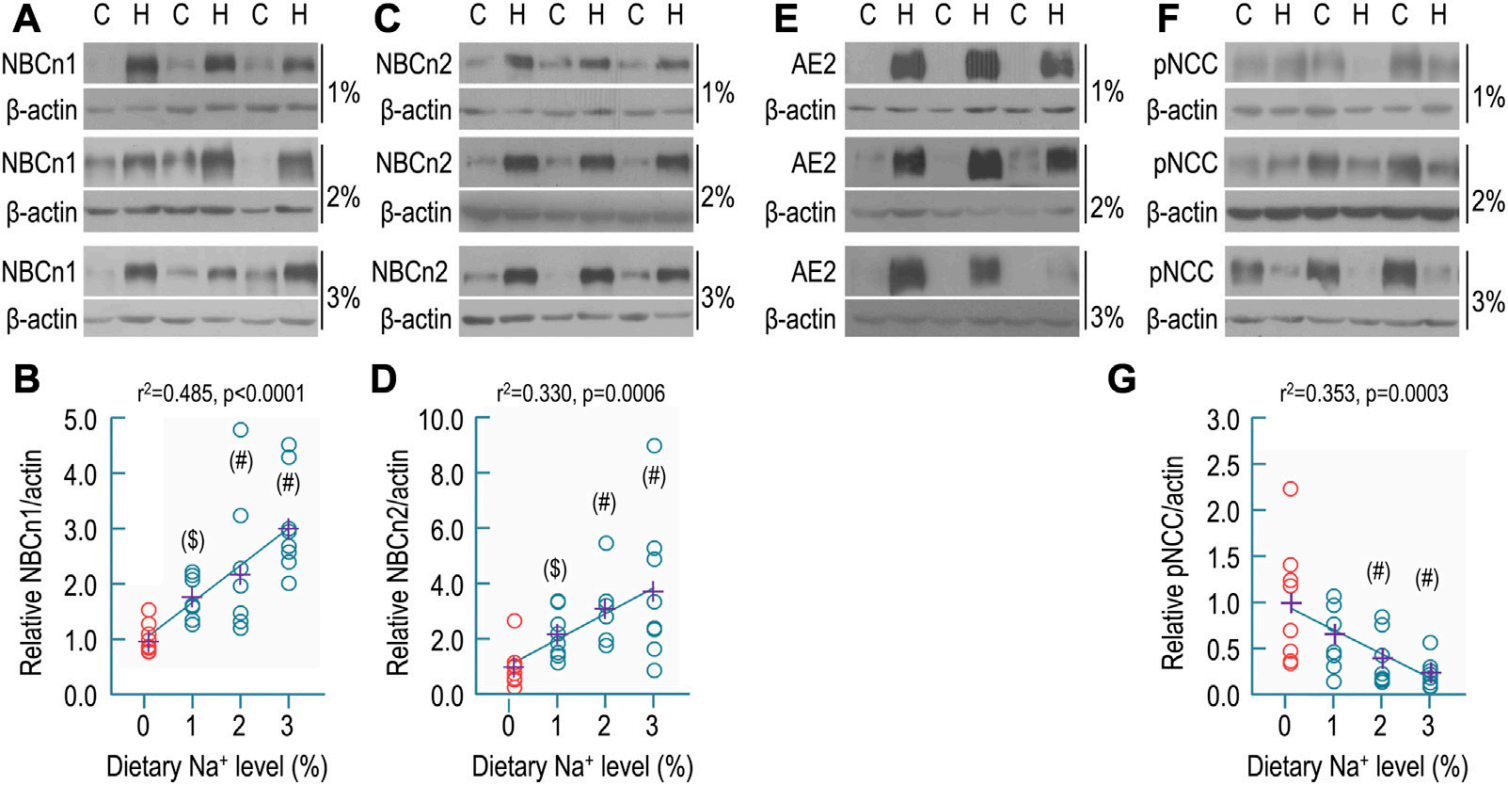
Effect of dietary sodium on the expression of NBCn1, NBCn2, AE2, and pNCC in rat kidney. **(A)** Representative western blots for the expression of NBCn1 in the kidney. **(B)** Summary for the relative abundance of renal NBCn1. **(C)** Representative western blots for the expression of NBCn2 in the kidney. **(D)** Summary for the relative abundance of renal NBCn2. **(E)** Representative western blots for the expression of AE2 in the kidney. The abundance of AE2 was strikingly increased by dietary sodium. It was not practical to determine the relative ratio of AE2 abundance of high sodium groups over the control. **(F)** Representative western blots for the expression of pNCC in the kidney. **(G)** Summary for the relative abundance of renal pNCC. Western blotting analyses were performed with the membrane preparations of whole kidney. C: control normal (0.1% Na^+^). H: high sodium diet. Each data point (circles) represents an individual rat (*n* = 8 for each condition). The data points of the control (0.1% of Na^+^) are presented in red. The crosses in purple indicate the means of protein levels in each condition. #: significantly different from the control (red) by one-way ANOVA followed by Dunnet’s multiple comparison (*p* < 0.05). $: significantly different from the control by Student’s t-test (*p* < 0.05).

Figure 5A shows representative Western blots for the expression of renal NBCn1. As summarized in Figure 5B, compared to the control (1.00 ± 0.27), the relative abundances of NBCn1 in the high-sodium groups were significantly increased to 1.78 ± 0.38 for 1% Na^+^, 2.20 ± 1.24 for 2% Na^+^, and 3.05 ± 0.89 for 3% Na^+^, respectively. By regression analysis, the relative abundance of NBCn1 was significantly positively correlated with the level of dietary sodium (*r* ^2^ = 0.485, *p* < 0.0001).

Shown in Figure 5C are representative Western blots for the expression of NBCn2 in the kidney. As summarized in Figure 5D, compared to the control (1.00 ± 0.74), the relative abundances of NBCn2 were significantly increased to 2.18 ± 0.86 for 1% Na^+^, 3.09 ± 1.13 for 2% Na^+^, and 3.74 ± 2.63 for 3% Na^+^, respectively. Again, by regression analysis, the relative abundance of NBCn2 was significantly positively correlated with the level of dietary sodium (*r* ^2^ = 0.330, *p* = 0.006).

Figure 5E shows representative Western blots for the effect of dietary sodium on the expression of renal AE2. Compared to the control, the abundances of AE2 were so strikingly increased that the AE2 in the control was almost invisible even when the AE2 was over-exposed in the high-sodium groups. Thus, it was not practical to obtain the relative ratio of AE2 of the high-sodium groups over the control.

Finally, we examined the effect of dietary sodium on the expression of Na-Cl cotransporter NCC (SLC12A3) in rat kidney. NCC is the major transporter in the capical DCT responsible for NaCl reabsorption by the DCT (Murthy et al., 2017; Shekarabi et al., 2017). Phosphorylation increases the activity of NCC (Glover et al., 2010). Others have shown that high-salt diet decreases the abundance of phosphorylated NCC (pNCC) in mouse kidney (Torres-Pinzon et al., 2021). Shown in Figure 5F are representative Western blots for the expression of pNCC in rat kidney. As summarized in Figure 5G, compared to the control (1.00 ± 0.66), the relative abundances of pNCC in the high-sodium groups were decreased to 0.66 ± 0.37 for 1% Na^+^, 0.40 ± 0.28 for 2% Na^+^, and 0.24 ± 0.16 for 3% Na^+^, respectively. The decreases for the groups of 2% and 3% Na^+^ are statistically significant compared to the control. Moreover, the relative abundance of pNCC in rat kidney was significantly negatively correlated with dietary sodium level by regression analysis (*r* ^2^ = 0.353, *p* = 0.0003). Our data are consistent with the previous study on mice (Torres-Pinzon et al., 2021).

### Effect of dietary sodium on expression of IRBIT, L-IRBIT, and PP1 in rat kidney

**FIGURE 6:**
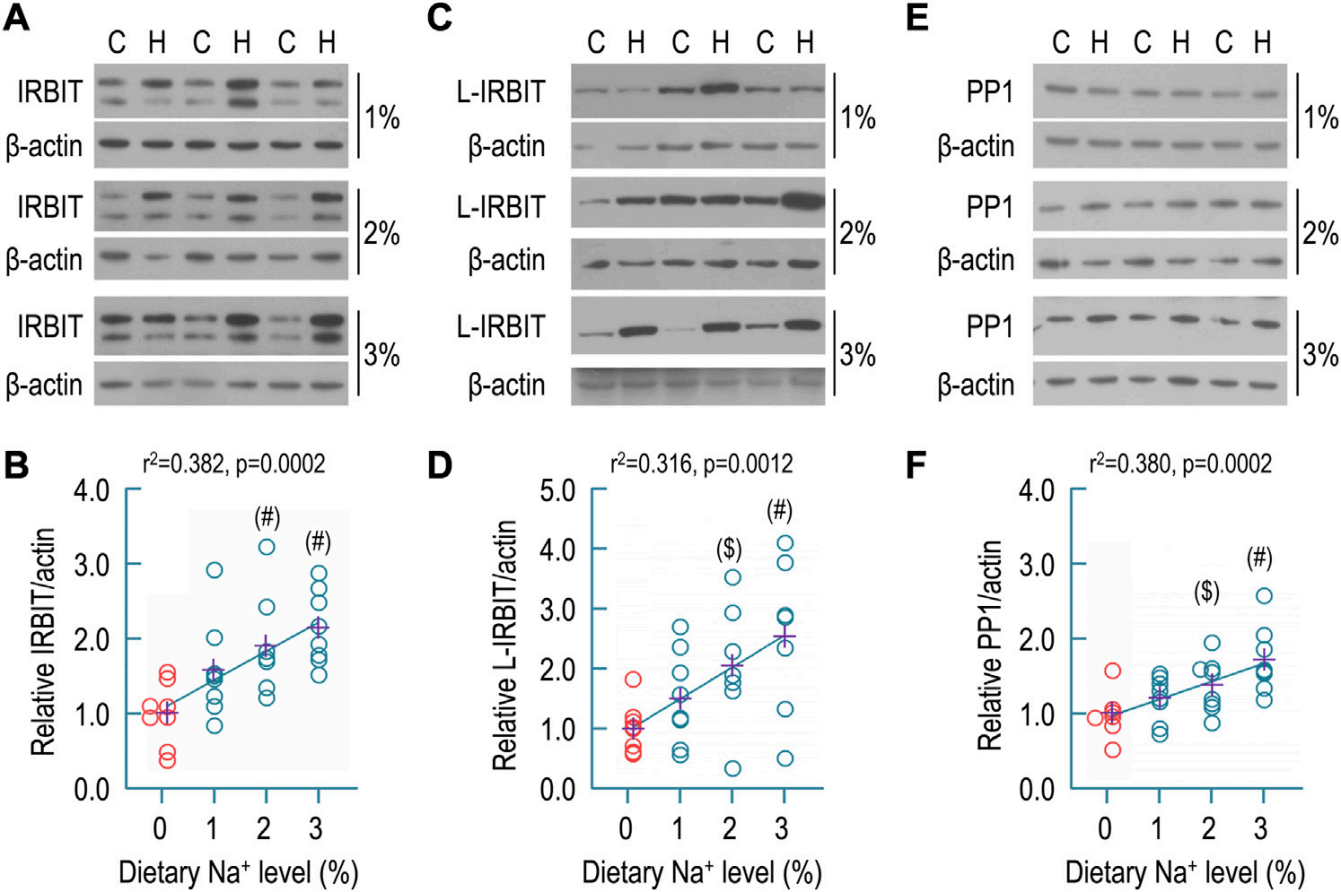
Effect of dietary sodium on expression of IRBIT, L-IRBIT, and PP1 in rat kidney. **(A)** Representative Western blots for the expression of IRBIT in the kidney. **(B)** Summary for the relative abundance of renal IRBIT. **(C)** Representative Western blots for the expression of L-IRBIT in the kidney. **(D)** Summary for the relative abundance of renal L-IRBIT. **(E)** Representative western blots for the expression of PP1 in the kidney. **(F)** Summary for the relative abundance of renal PP1. Western blotting analyses were performed with the membrane preparations of whole kidney. C: control normal (0.1% Na^+^); H: high sodium diet. Each data point represents an individual rat (*n* = 7–8 for each condition). The data points of the control (0.1% of Na^+^) are presented in red. The crosses in purple indicate the means of protein levels in each condition. #: significantly different from the control (red) of 0.1% Na^+^ by one-way ANOVA followed by Dunnet’s multiple comparison (*p* < 0.05). $: significantly different from the control by Student’s t-test (*p* < 0.05). In Panel **(B)** (the condition of 0.1% Na^+^), and Panel **(F)** (the conditions of 0.1% and 2% Na^+^), several data points were artificially shifted to left because these were virtually overlapped with other data points.

Next, we examined the effect of dietary sodium on the expression of IRBIT, L-IRBIT, and PP1 in the membrane preparations of whole rat kidney. Shown in Figure 6A are representative Western blots for the expression of IRBIT in the kidney. As summarized in Figure 6B, compared to the control (1.00 ± 0.42), the relative abundances of IRBIT were increased to 1.58 ± 0.65 for 1% Na^+^, 1.90 ± 0.65 for 2% Na^+^, and 2.15 ± 0.49 for 3% Na^+^, respectively. The increases in the groups of 2% and 3% Na^+^ are statistically compared to the control. Moreover, the relative abundance of renal IRBIT was significantly positively correlated to the level of dietary sodium by regression analysis (*r* ^2^ = 0.382, *p* = 0.0002).

Similar analyses were performed with L-IRBIT. Shown in Figure 6C are representative Western blots for the expression of L-IRBIT in rat kidney. As summarized in Figure 6D, compared to the control (1.00 ± 0.41), the relative abundances of L-IRBIT were increased to 1.51 ± 0.78 for 1% Na^+^, 2.05 ± 1.03 for 2% Na^+^, and 2.54 ± 1.28 for 3% Na^+^, respectively. The increases in the groups of 2% and 3% Na^+^ were statistically significant compared to the control. Moreover, the relative abundance of L-IRBIT was significantly positively correlated to the level of dietary sodium by regression analysis (*r* ^2^ = 0.316, *p* = 0.0012).

Figure 6E shows representative Western blots for PP1 expression in rat kidney. As summarized in Figure 6F, compared to the control (1.00 ± 0.29), the relative abundances of PP1 were increased to 1.20 ± 0.30 for 1% Na^+^, 1.38 ± 0.36 for 2% Na^+^, and 1.72 ± 0.44 for 3% Na^+^, respectively. The increases in the groups of 2% and 3% Na^+^ are statistically significantly compared to the control. Again, the relative abundance of renal PP1 was significantly positively correlated to dietary sodium level by regression analysis (*r* ^2^ = 0.380, *p* = 0.0002).

### Interaction of NBCn1 with IRBIT/L-IRBIT

**FIGURE 7:**
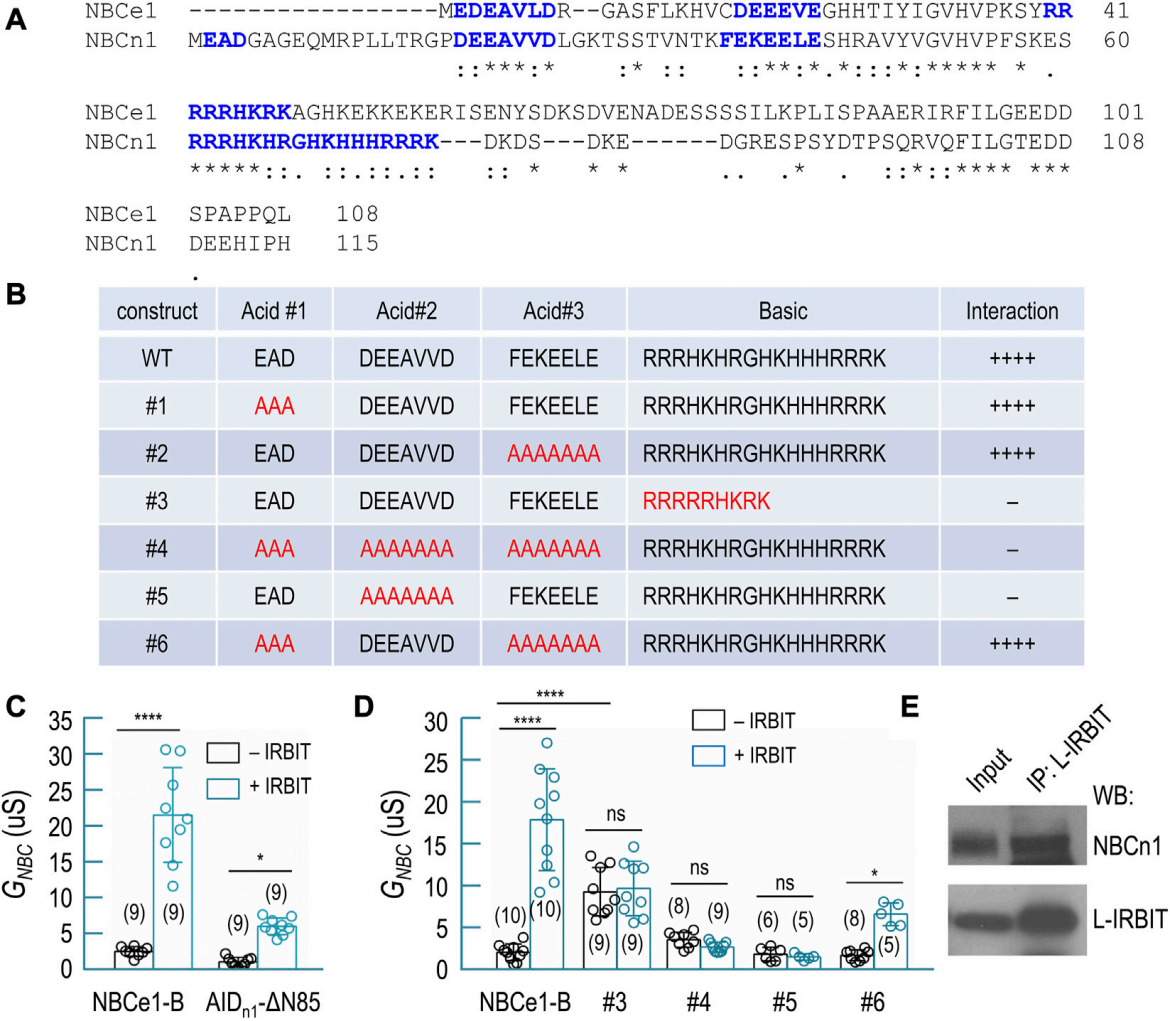
Interaction of IRBIT and L-IRBIT with NBCn1. **(A)** Alignment of the initial Nt of NBCn1 with the unique Nt of NBCe1 (variant B). **(B)** Characterization of structural elements of NBCn1 essential for binding of IRBIT by yeast-2-hybrid assay. **(C)** Summary for the effect of the auto-inhibition domain of NBCn1 (residues 1–115) on transport activity of NBCe1. **(D)** Summary for the transport activity of NBCe1 containing variants of AID of NBCn1. **(E)** Co-immunoprecipitation analysis showing the interaction of NBCn1 with L-IRBIT in rat kidney. In Panels **(C,D)**, data are presented in mean ± S.D. in Panels **(C,D)**. The numerals in the parentheses indicate the number of individual oocytes included in each bars. For statistical analysis, one-way ANOVA followed by Tukey’s comparison was performed. *p < 0.05; ****p < 0.001. ns: not significant.

IRBIT and L-IRBIT (collectively the IRBITs) can bind to and greatly stimulate the activities of many NBCs, such as NBCe1-B, NBCn1, and NBCn2. These NBCs all contain an auto-inhibition domain (AID) as well as an IRBIT-binding domain at the flexible amino-termini (for review, see refs. (Parker and Boron, 2013; Liu et al., 2015). Figure 7A shows an alignment of the amino-termini of NBCe1-B and NBCn1. The interaction of IRBIT with NBCe1-B essentially requires the presence of the two acidic motifs “EDE” and “DEEEVE” as well as the basic cluster “RRRRRHKRK” (Lee and Boron, 2018; Su et al., 2021).

The amino-terminus of NBCn1 contains three acid motifs and a much longer basic cluster spanning from Arg^61^ through Lys^77^. We employed yeast-2-hybrid (Y2H) to investigate the structural determinants in NBCn1 for its interaction with IRBIT. Based on an amino-terminal fragment of NBCn1 containing residues 1–115, we generated a series of mutants by modifying the three acid motifs and/or the basic cluster (Figure 7B). Our data indicate that the interaction between NBCn1 and IRBIT essentially requires the simultaneous presence of the acidic motif “DEEAVVD” and the basic cluster in the amino-terminus of NBCn1. Neither the acidic motif “EAD” nor “FEKEELE” was essential for the interaction of NBCn1 with IRBIT. Interestingly, replacing the basic cluster of NBCn1 with that of NBCe1 abolished the interaction between NBCn1 and IRBIT (construct #3, Figure 7B).

Removing the AID-containing region (residues 1–85) fully activates NBCe1-B (McAlear et al., 2006; Lee et al., 2012; Su et al., 2021). We generated a chimera (designated as AID_n1_-ΔN85) of NBCn1 and NBCe1-B by fusing residues 1–115 of NBCn1 to the amino-terminal end of ΔN85, an NBCe1-B mutant lacking residues 1–85. We expressed AID_n1_-ΔN85 in *Xenopus* oocyte to examine the inhibitory effect of the AID of NBCn1 on the activity of NBCe1 by electrophysiology. As summarized in Figure 7C, IRBIT fully stimulated wild-type NBCe1-B (2.69 ± 0.67 μS without IRBIT vs. 21.63 ± 6.56 μS with IRBIT, *p* < 0.001). However, IRBIT just modestly stimulated AID_n1_-ΔN85 (1.25 ± 0.62 μS without IRBIT vs. 6.17 ± 1.15 μS with IRBIT, *p* < 0.05). The data indicate that the AID of NBCn1 elicited much greater inhibition on NBCe1 than the AID of NBCe1-B. This greater inhibition was presumably due to the extremely long basic cluster of NBCn1. The basic cluster has been shown to play a critically important role in the auto-inhibition of NBCe1 (Lee and Boron, 2018; Su et al., 2021).

Starting from AID_n1_-ΔN85, we generated a series of mutants by introducing mutations to the AID corresponding to the constructs shown in Figure 7B. We measured the activity of these constructs ± IRBIT by electrophysiology. Generally, the electrophysiology data confirmed the conclusion derived from the Y2H data (Figure 7D). For example, based on AID_n1_-ΔN85, replacing “DEEAVVD” with “AAAAAAA” (construct #5) abolished the stimulation of the transporter by IRBIT (1.93 ± 0.80 μS without IRBIT vs. 1.61 ± 0.37 μS with IRBIT, *p* > 0.05), suggesting that “DEEAVVD” is essential for the interaction of NBCn1 with IRBIT. Again, replacing the basic cluster of NBCn1 with that of NBCe1-B (construct #3) abolished the stimulation of the transporter by IRBIT (9.34 ± 2.87 μS without IRBIT vs. 9.75 ± 3.25 μS with IRBIT, *p* > 0.05). However, in the absence of IRBIT, the basal activity of construct #3 is significantly higher than that of wild-type NBCe1-B, suggesting lower magnitude of auto-inhibition for construct #3.

Taken together, the above Y2H and electrophysiology data suggest that the interaction of NBCn1 with IRBIT requires the acidic motif “DEEAVVD” and the basic cluster in NBCn1. Moreover, it is essential to maintain sufficient number of alkaline residues in the basic cluster for the effective interaction between NBCn1 and IRBIT.

Finally, by co-immunoprecipitation with anti-L-RBIT, we obtained NBCn1 from rat kidney (Figure 7E). It is proposed that the IRBITs are involved in the regulation of NBCn1 and NBCn2 in the kidney (Wang J. L. et al., 2020). The co-immunoprecipitation data suggest the presence of protein interaction between NBCn1 and L-IRBIT in rat kidney.

## Discussion

**FIGURE 8:**
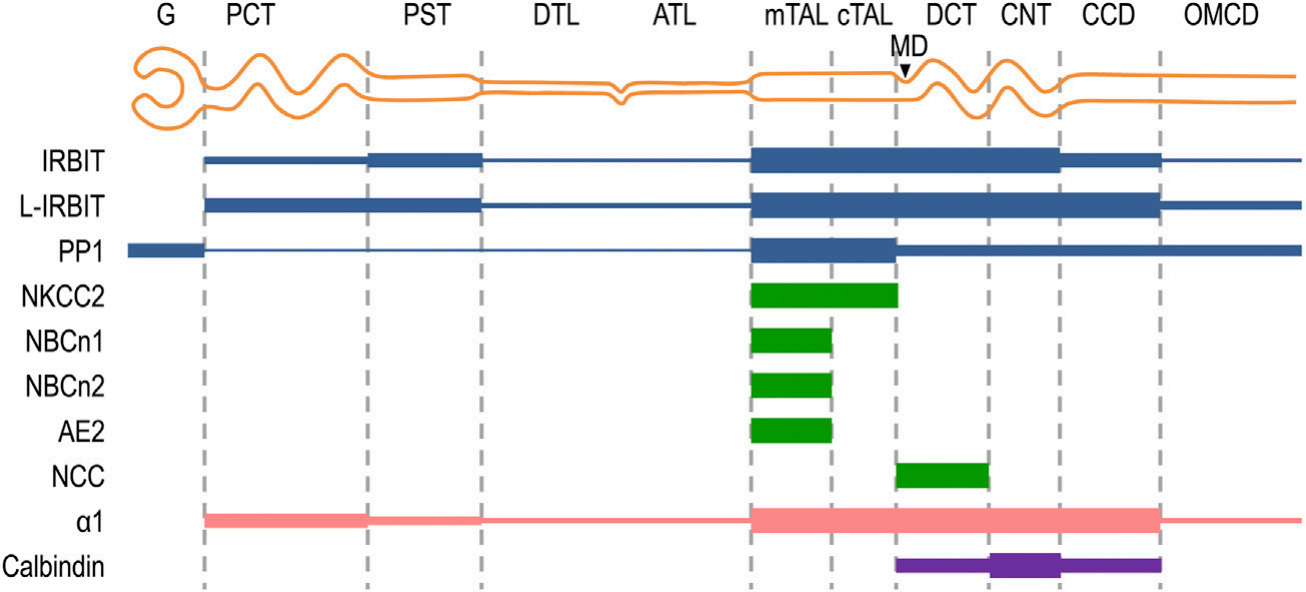
Schematic diagram summarizing the localization of NBCn1, IRBIT, L-IRBIT, PP1, NKCC2 along the nephron. The expression localizations of IRBIT, L-IRBIT, and PP1 in the kidney were systematically characterized for the first time in the present study. The expression of NBCn1, NBCn2 and AE2 in the mTAL was established previously (Alper et al., 1997; Kim et al., 2003; Praetorius et al., 2004; Guo et al., 2017). The present study confirmed the localization of NBCn1 in the mTAL, but not in other segments of the nephron. The specific localization of NCC in DCT was established previously (Campean et al., 2001). NKCC2 is a specific marker for the mTAL and cTAL (Ecelbarger et al., 1996; Gimenez and Forbush, 2003). Calbindin is a specific marker for DCT, CNT, and CCD (Campean et al., 2001; Wetzel and Sweadner, 2001). G, glomerulus; PCT, proximal convoluted tubule; PST, proximal straight tubule; DTL, descending thin limb; ATL, ascending thin limb; mTAL, medullary thick ascending limb; cTAL, cortical thick ascending limb; MD, macula densa; DCT, distal convoluted tubule; CNT, cortical connecting tubule; CCD, cortical collecting duct; OMCD, outer medullary collecting duct.

In the present study, we performed a careful characterization for the expression and localization of NBCn1, IRBIT, L-IRBIT, and PP1 in rat kidney. As summarized in Figure 8, the expression of NBCn1 is limited to the mTAL along the nephron. IRBIT and L-IRBIT are expressed almost everywhere along the nephron in rat kidney. The expression of IRBIT is most abundant from the mTAL through the CNT, relatively lower in the PST and the CCD, and even lower in other regions of the nephron. The expression of L-IRBIT is most abundant from the mTAL through the CCD, relatively lower in the PCT and the PST, and even lower in other regions of the nephron. PP1 is most abundant in the TAL, and relatively lower from the DCT through the MCD. PP1 is also expressed in high abundance in the glomerulus.

The expression of NBCn1, NBCn2, and AE2 are significantly stimulated by increasing dietary sodium intake, whereas the abundance of pNCC is significantly decreased by dietary sodium. Finally, the abundance of IRBIT, L-IRBIT, and PP1 are positively correlated to dietary sodium intake.

### The role of NBCn1, NBCn2, and AE2 in renal sodium handling

The SLC4 family transporters NBCn1, NBCn2, and AE2 are all expressed in the basolateral membrane of mTAL (Alper et al., 1997; Kim et al., 2003; Praetorius et al., 2004; Guo et al., 2017). In a previous study (Wang J. L. et al., 2020), the authors have proposed that NBCn1, NBCn2, and AE2 are involved in the regulation of NaCl handling by mTAL. According to the model in this previous study, the NBCs (NBCn1 and NBCn2) by coordination with AE2 mediate the back flux of Na^+^ and Cl^−^ under high salt condition, counteracting the NaCl reabsorption mediated by the apical NKCC2, the basolateral Na^+^-K^+^-ATPase and Cl^−^ channel. Supporting this theory are the following lines of observations. Firstly, NaCl overload upregulates the expression of NBCn1, NBCn2, and AE2 in rat kidney (Quentin et al., 2004; Wang J. L. et al., 2020). Secondly, NaHCO_3_ overload upregulates the expression of AE2 in rat kidney (Quentin et al., 2004). Finally, NaHCO_3_ upregulates the expression of renal NBCn1 and NBCn2 with a greater effect than NaCl does (Wang J. L. et al., 2020). The stimulatory effect of NaHCO_3_ on AE2 expression disagrees with its role in HCO_3_^−^ reabsorption inasmuch as an upregulation in AE2 would increase the capacity of HCO_3_^−^ reabsorption of the mTAL, causing further alkalosis. However, the upregulation of AE2 by NaHCO_3_ could be well explained by the proposed role of AE2 in NaCl reabsorption.

In the previous study (Wang J. L. et al., 2020), the greater effect of NaHCO_3_ on the expression of NBCn1 and NBCn2 than NaCl indicates a net effect of sodium. In the present study, we find the abundances of renal NBCn1 and NBCn2 are proportionally upregulated by increasing dietary sodium intake. The abundance of renal AE2 is also strikingly upregulated by increasing dietary sodium. These new findings further corroborate the idea that NBCn1 and NBCn2 together with AE2 inhibit renal reabsorption of NaCl under high salt condition. Moreover, the new findings indicate that sodium is the major factor that accounts for the upregulation of these bicarbonate transporters under high salt condition.

Dysfunctions of *SLC4A2* encoding AE2 and *SLC4A7* encoding NBCn1 are associated with hypertension (Sober et al., 2009; Boedtkjer et al., 2011; Ehret et al., 2011; Wang et al., 2017). The inhibitory role of NBCn1 and AE2 in renal sodium reaborption indicates that the development of hypertension associated with the dysfunction of *SLC4A7* and *SLC4A2* could be the result of renal sodium retention. Nevertheles, the development of hypertension is often a multifactorial event. Others have shown that NBCn1 is involved in the regulation of the contraction of blood vessel smooth msucle (Boedtkjer et al., 2011; Jiang et al., 2019), indicating the presence of a cardiovascular component in the development of hypertension caused by the dysfuction of *SLC4A7*.

### The role of IRBIT in renal sodium handling

IRBIT is able to interact with and regulate the function of a series of proteins with distinct physiological functions. The binding partners of IRBIT include membrane channels, such as the endoplasmic reticulum Ca^2+^ channel (InsP3R) and cystic fibrosis transmembrane conductance regulator (CFTR), and membrane transporters such as NHE3 (He et al., 2008; He et al., 2010; Tran et al., 2013; He et al., 2016), NBCe1-B (Shirakabe et al., 2006), NBCn1 (Hong et al., 2013; Wang J. L. et al., 2020), NBCn2 (Wang J. L. et al., 2020), AE2 (Itoh et al., 2021), SLC26A6 (Park et al., 2013; Khamaysi et al., 2019), SLC13A2, Na^+^-succinate cotransporter SLC13A3 (Khamaysi et al., 2019), etc. IRBIT can also interact with protein kinases, such as calcium calmodulin-dependent kinase II alpha (CaMKIIα) (Kawaai et al., 2015), as well as lipid kinases, such as PIPKIα and PIPKIIα (Ando et al., 2015).

Virtually all the above-mentioned channels and transporters that interact with IRBIT are expressed in the kidney. For example, NHE3 (SLC9A3) is expressed in the apical membrane of the proximal tubules and TAL, playing a major role in the regulation of acid-base balance and the reabsorption of Na^+^. NHE3 activity is stimulated by angiotensin II (Ang II) in a manner dependent on IRBIT and CaMKIIα (He et al., 2010). NHE3 in the proximal tubule is shown to be involved in the development of hypertension induced by Ang II (Nwia et al., 2022). The apical SLC26A6 in the proximal tubule plays a dispensible role in the regulation of NaCl homeostasis in the body (Knauf et al., 2001; Knauf et al., 2019). The Na^+^/dicarboxylate cotransporter SLC13A2 is expressed in the apical membrane of the proximal tubule in human kidney (Lee et al., 2017). Here, SLC13A2 forms complex with SLC26A6 (Ohana et al., 2013). The Na^+^-succinate cotransporter SLC13A3/NaDC-3 is abundantly expressed at the basolateral membrane of renal proximal tubule (Burckhardt et al., 2005; Breljak et al., 2016). In the distal segments of the nephron, CFTR in concert with SLC26A4 is involved in the regulation of HCO_3_^−^ secretion under metabolic alkalosis (Berg et al., 2021a; Berg et al., 2021b).

In the present study, we find that IRBIT is almost ubiqitously expressed along the nephron. The wide distribution of IRBIT and its interaction partners in the kidney implies profound significance of IRBIT in renal physiology. It is conceivable that, in the kidney, IRBIT is involved in the regulation of many different physiological processes by regulating diverse target proteins. In the previous study (Wang J. L. et al., 2020), the authors have shown that NaCl overload generally upregulates the expression of IRBIT in rat kidney. In the present study, we show that the abundance of IRBIT is proportionally increased with the level of dietary sodium, indicating that sodium is the major factor that modulate the expression of renal IRBIT under high salt condition. We propose that IRBIT plays an important role to inhibit renal sodium reabsorption.

Given the demonstrated stimulatory effect of IRBIT on NBCn1, NBCn2 (Hong et al., 2013; Wang J. L. et al., 2020), it is possible that, under high sodium condition, IRBIT stimulates the activities of the basolateral NBCn1 and NBCn2—the expressions of which are upregulated under high sodium condition—to inhibit NaCl reabsorption in the mTAL.

IRBIT activates NBCe1-B by abolishing the action of the auto-inhibition domain at the amino-terminal end of NBCe1-B (Lee et al., 2012; Lee and Boron, 2018; Su et al., 2021). The auto-inhibition domains of NBCn1 and NBCn2 are homologous to that of NBCe1-B. It is very likely that IRBIT activates NBCn1 and NBCn2 by a molecular mechanism similar to that for NBCe1-B.

### The role of L-IRBIT in renal sodium handling

L-IRBIT is highly homologous to IRBIT with more than 93% of sequence identity regardless of their unique Nt appendages. Given the high degree of sequence identity, it is very likely that L-IRBIT can interact with most of the binding partners of IRBIT (Kawaai et al., 2017). Previous studies have shown that L-IRBIT interacts with and stimulates the activities of NHE3 (Kawaai et al., 2017), NBCe1-B (Yamaguchi and Ishikawa, 2014; Wang M. et al., 2020), NBCn1 (Wang J. L. et al., 2020), NBCn2 (Wang J. L. et al., 2020), and AE2 (Itoh et al., 2021). L-IRBIT can also interact with CaMKIIα and InsP3R (Kawaai et al., 2017).

In the present sudy, we find that L-IRBIT is almost ubiquitously expressed in the kidney. The broad distribution of L-IRBIT implies that L-IRBIT play diverse roles in renal physiology. Considering the fact that the abundance of L-IRBIT is proportionally upregulated by increasing dietary sodium, we propose that L-IRBIT plays an inhibitory role in renal sodium reabsorption under high sodium condition. Given the demonstrated stimulatory effect of L-IRBIT on NBCn1, NBCn2, and AE2 (Hong et al., 2013; Wang J. L. et al., 2020; Itoh et al., 2021), L-IRBIT could do so by stimulating the basolateral NBCn1, NBCn2, and AE2—the expressions of which are all upregulated under high sodium condition—in the mTAL, therefore increasing the back flux of Na^+^ and Cl^−^, thus decreasing the reabsorption of Na^+^ and Cl^−^.

### The role of PP1 in renal sodium handling

In the present study, we show that PP1 is widely expressed along the nephron with the highest abundance in the TAL. The abundance of renal PP1 is proportionally downregulated by dietary sodium. We propose that PP1 plays an important role to inhibit renal sodium reasorption under high sodium condition. Consistent with this hypothesis, knockingout the inbibitor-1 of PP1 causes hypotension in mouse (Picard et al., 2014).

PP1 could inhibit renal sodium reabsorption *via* diverse mechanisms depending on the specific segments of the nephron. When heterologously expressed in HeLa cells, the activity of NBCn1 is stimulated by PP1 and inhibited by kinase SPAK (Hong et al., 2013). The observations suggest that NBCn1 is activated by dephosphorylation. Given the expressional upregulation of both PP1 and NBCn1 by dietary sodium, we hypothesize that PP1 stimulates NBCn1 in mTAL, therefore inhibiting sodium reabsorption by the mTAL under high sodium condition. So far, little is known about the phosphorylation of NBCn1. The proposed activation of NBCn1 by PP1 call for future studies to address the molecular mechanism underlying the phosphorylation regulation of NBCn1.

Elsewhere, PP1 could inhibit renal sodium reabsorption by down-regulating the activity of the apical NCC in the DCT, where NCC plays a major role in NaCl reabsorption (for review, see refs. (Murthy et al., 2017; Shekarabi et al., 2017; Azlan et al., 2021). Others have shown that dephosphorylation decreases NCC activity in *Xenopus* oocytes (Glover et al., 2010) and in DCT epithelial cells (Picard et al., 2014). A number of studies have shown that PP1 is involved in the phosphorylation regulation of NCC in the kidney (Picard et al., 2014; Penton et al., 2019; Poulsen et al., 2021). Indeed, inhibition of PP1 by expressing I-1, a spefcific PP1 inhibitor, substantially increases the phosphorylation levl as well as the activity of NCC in *Xenopus* oocytes (Picard et al., 2014). Consistently, knocking-out I-1 substantially decreases the phoshorylation level of NCC in mouse kidney (Picard et al., 2014; Penton et al., 2019). In the present study, we show that the renal abundance of pNCC (phosphorylated NCC) is decreased under high sodium intake, an observation consistent with previous studies (Shao et al., 2021; Torres-Pinzon et al., 2021). This decrease in pNCC is likely attributable to the increased expression of PP1.

### Concluding remarks

The present study provided insights into the molecular network underlying the regulation of renal sodium handling. In the mTAL, NaCl reabsorption is facilitated by NKCC2 and NHE3 at the apical membrane, and inhibited by the SLC4 bicarbonate transporters NBCn1, NBCn2, and AE2 at the basolateral membrane. Under high sodium conditions, the bicarbonate transporters NBCn1, NBCn2, and AE2, at least some of them, could be functionally upregulated by protein interaction with the IRBITs and by dephosphorylation by, e.g., protein phosphatase PP1. The functional interaction between the bicarbonate transporters (e.g., NBCn1) and PP1 in the kidney remains to be addressed in future. Moreover, the molecular mechanism underlying the regulation of these bicarbonate transporters by phosphorylation/dephosphorylation remains unkown and calls for future studies. Finally, given the almost ubiquitous expression in the kidney, the IRBITs and PP1 could be involved in the regulation of sodium handling in renal segments beyond the mTAL.

## Data Availability

The original contributions presented in the study are included in the article/Supplementary Materials, further inquiries can be directed to the corresponding authors.

## References

[B1] Alper S. L. Stuart-Tilley A. K. Biemesderfer D. Shmukler B. Brown D. (1997). Immunolocalization of AE2 anion exchanger in rat kidney. Am. J. Physiol. 273, F601–F614. 10.1152/ajprenal.1997.273.4.F601 9362338

[B2] Ando H. Hirose M. Gainche L. Kawaai K. Bonneau B. Ijuin T. (2015). IRBIT interacts with the catalytic core of phosphatidylinositol phosphate kinase type Iα and IIα through conserved catalytic aspartate residues. PLoS One 10, 01415699. 10.1371/journal.pone.0141569 PMC4624786 26509711

[B3] Ares G. R. Caceres P. S. Ortiz P. A. (2011). Molecular regulation of NKCC2 in the thick ascending limb. Am. J. Physiol.-Renal Physiol. 301, F1143–F1159. 10.1152/ajprenal.00396.2011 21900458 PMC3233874

[B4] Azlan N. F. M. Koeners M. P. Zhang J. (2021). Regulatory control of the Na-Cl co-transporter NCC and its therapeutic potential for hypertension. Acta Pharmacol. Sin. B 11, 1117–1128. 10.1016/j.apsb.2020.09.009 PMC8144889 34094823

[B5] Bankir L. Figueres L. Prot-Bertoye C. Bouby N. Crambert G. Pratt J. H. (2020). Medullary and cortical thick ascending limb: Similarities and differences. Am. J. Physiol-Renal Physiol. 318, F422–F442. 10.1152/ajprenal.00261.2019 31841389

[B6] Barkley R. A. Chakravarti A. Cooper R. S. Ellison R. C. Hunt S. C. Province M. A. (2004). Positional identification of hypertension susceptibility genes on chromosome 2. Hypertension 43, 477–482. 10.1161/01.HYP.0000111585.76299.f7 14732741

[B7] Bazua-Valenti S. Castaneda-Bueno M. Gamba G. (2016). Physiological role of SLC12 family members in the kidney. Am. J. Physiol.-Renal Physiol. 311, F131–F144. 10.1152/ajprenal.00071.2016 27097893

[B8] Berg P. Svendsen S. L. Hoang T. T. L. Praetorius H. A. Sorensen M. V. Leipziger J. (2021a). Impaired renal HCO_3_ ^−^ secretion in CFTR deficient mice causes metabolic alkalosis during chronic base-loading. Acta Physiol. 231, e13591. 10.1111/apha.13591 33270356

[B9] Berg P. Svendsen S. L. Sorensen M. V. Schreiber R. Kunzelmann K. Leipziger J. (2021b). The molecular mechanism of CFTR- and secretin-dependent renal bicarbonate excretion. J. Physiol. 599, 3003–3011. 10.1113/jp281285 33963548

[B10] Boedtkjer E. Praetorius J. Matchkov V. V. Stankevicius E. Mogensen S. Fuchtbauer A. C. (2011). Disruption of Na^+^,HCO_3_ ^−^ cotransporter NBCn1 (slc4a7) inhibits NO-mediated vasorelaxation, smooth muscle Ca^2+^ sensitivity, and hypertension development in mice. Circulation 124, 1819–1829. 10.1161/CIRCULATIONAHA.110.015974 21947296

[B11] Boger C. A. Gorski M. McMahon G. M. Xu H. Chang Y. C. van der Most P. J. (2017). *NFAT5* and *SLC4A10* loci associate with plasma osmolality. J. Am. Soc. Nephrol. 28, 2311–2321. 10.1681/ASN.2016080892 28360221 PMC5533231

[B12] Bourgeois S. Masse S. Paillard M. Houillier P. (2002). Basolateral membrane Cl^−^-Na^+^-and K^+^-coupled base transport mechanisms in rat mTALH. Am. J. Physiol.-Renal Physiol. 282, F655–F668. 10.1152/ajprenal.00220.2000 11880327

[B13] Breljak D. Ljubojevic M. Hagos Y. Micek V. Eror D. B. Madunic I. V. (2016). Distribution of organic anion transporters NaDC3 and OAT1-3 along the human nephron. Am. J. Physiol.-Renal. Physiol. 311, F227–F238. 10.1152/ajprenal.00113.2016 27053689

[B14] Burckhardt B. C. Lorenz J. Kobbe C. Burckhardt G. (2005). Substrate specificity of the human renal sodium dicarboxylate cotransporter, hNaDC-3, under voltage-clamp conditions. Am. J. Physiol.-Renal. Physiol. 288, F792–F799. 10.1152/ajprenal.00360.2004 15561973

[B15] Caceres P. S. Ortiz P. A. (2019). Molecular regulation of NKCC2 in blood pressure control and hypertension. Curr. Opin. Nephrol. Hypertens. 28, 474–480. 10.1097/Mnh.0000000000000531 31313674 PMC7226929

[B16] Campean V. Kricke J. Ellison D. Luft F. C. Bachmann S. (2001). Localization of thiazide-sensitive Na^+^-Cl^−^ cotransport and associated gene products in mouse DCT. Am. J. Physiol.-Renal. Physiol. 281, F1028–F1035. 10.1152/ajprenal.0148.2001 11704553

[B17] Carey R. M. Schoeffel C. D. Gildea J. J. Jones J. E. McGrath H. E. Gordon L. N. (2012). Salt sensitivity of blood pressure is associated with polymorphisms in the sodium-bicarbonate cotransporter. Hypertension 60, 1359–1366. 10.1161/Hypertensionaha.112.196071 22987918 PMC3495588

[B18] Chen L. H. Chou C. L. Knepper M. A. (2021). A comprehensive map of mRNAs and their isoforms across all 14 renal tubule segments of mouse. J. Am. Soc. Nephrol. 32, 897–912. 10.1681/Asn.2020101406 33769951 PMC8017530

[B19] Dimke H. Schnermann J. (2018). Axial and cellular heterogeneity in electrolyte transport pathways along the thick ascending limb. Acta Physiol. 223, e13057. 10.1111/apha.13057 29476644

[B20] Ecelbarger C. A. Terris J. Hoyer J. R. Nielsen S. Wade J. B. Knepper M. A. (1996). Localization and regulation of the rat renal Na^+^-K^+^-2Cl^−^ cotransporter, BSC-1. Am. J. Physiol. 271, F619–F628. 10.1152/ajprenal.1996.271.3.F619 8853424

[B21] Ehret G. B. Munroe P. B. Rice K. M. Bochud M. Johnson A. D. Chasman D. I. (2011). Genetic variants in novel pathways influence blood pressure and cardiovascular disease risk. Nature 478, 103–109. 10.1038/nature10405 21909115 PMC3340926

[B22] Gimenez I. Forbush B. (2003). Short-term stimulation of the renal Na-K-Cl cotransporter (NKCC2) by vasopressin involves phosphorylation and membrane translocation of the protein. J. Biol. Chem. 278, 26946–26951. 10.1074/jbc.M303435200 12732642

[B23] Glover M. Zuber A. M. Figg N. O'Shaughnessy K. M. (2010). The activity of the thiazide-sensitive Na^+^-Cl^−^ cotransporter is regulated by protein phosphatase PP4. Can. J. Physiol. Pharmacol. 88, 986–995. 10.1139/y10-080 20962898

[B24] Gonzalez-Vicente A. Saez F. Monzon C. M. Asirwatham J. Garvin J. L. (2019). Thick ascending limb sodium transport in the p athogenesis of hypertension. Physiol. Rev. 99, 235–309. 10.1152/physrev.00055.2017 30354966 PMC6335098

[B25] Groger N. Vitzthum H. Frohlich H. Kruger M. Ehmke H. Braun T. (2012). Targeted mutation of *SLC4A5* induces arterial hypertension and renal metabolic acidosis. Hum. Mol. Genet. 21, 1025–1036. 10.1093/hmg/ddr533 22082831

[B26] Guo L. Liu F. Chen S. Yang X. Huang J. He J. (2016). Common variants in the Na^+^-coupled bicarbonate transporter genes and salt sensitivity of blood pressure: The GenSalt study. J. Hum. Hypertens. 30, 543–548. 10.1038/jhh.2015.113 26582410 PMC4873465

[B27] Guo Y. M. Liu Y. Liu M. Wang J. L. Xie Z. D. Chen K. J. (2017). Na^+^/HCO_3_ ^−^ cotransporter NBCn2 mediates HCO_3_ ^−^ reclamation in the apical membrane of renal proximal tubules. J. Am. Soc. Nephrol. 28, 2409–2419. 10.1681/ASN.2016080930 28280139 PMC5533233

[B28] Guyton A. C. (1989). Dominant role of the kidneys and accessory role of whole-body auto-regulation in the pathogenesis of hypertension. Am. J. Hyperten. 2, 575–585. 10.1093/Ajh/2.7.575 2667575

[B29] He P. J. Zhao L. Q. No Y. R. Karvar S. Yun C. C. (2016). The NHERF1 PDZ1 domain and IRBIT interact and mediate the activation of Na^+^/H^+^ exchanger 3 by ANG II. Am. J. Physiol-Renal. Physiol. 311, F343–F351. 10.1152/ajprenal.00247.2016 27279487 PMC6189742

[B30] He P. Klein J. Yun C. C. (2010). Activation of Na^+^/H^+^ exchanger NHE3 by angiotensin II is mediated by inositol 1,4,5-triphosphate (IP3) receptor-binding protein released with IP3 (IRBIT) and Ca^2+^/calmodulin-dependent protein kinase II. J. Biol. Chem. 285, 27869–27878. 10.1074/jbc.M110.133066 20584908 PMC2934654

[B31] He P. Zhang H. Yun C. C. (2008). IRBIT, inositol 1,4,5-triphosphate (IP3) receptor-binding protein released with IP3, binds Na^+^/H^+^ exchanger NHE3 and activates NHE3 activity in response to calcium. J. Biol. Chem. 283, 33544–33553. 10.1074/jbc.M805534200 18829453 PMC2586249

[B32] Hong J. H. Yang D. Shcheynikov N. Ohana E. Shin D. M. Muallem S. (2013). Convergence of IRBIT, phosphatidylinositol (4,5) bisphosphate, and WNK/SPAK kinases in regulation of the Na^+^-HCO_3_ ^−^ cotransporters family. Proc. Natl. Acad. Sci. USA. 110, 4105–4110. 10.1073/pnas.1221410110 23431199 PMC3593885

[B33] Itoh R. Hatano N. Murakami M. Mitsumori K. Kawasaki S. Wakagi T. (2021). Both IRBIT and long-IRBIT bind to and coordinately regulate Cl^−^/HCO_3_ ^−^ exchanger AE2 activity through modulating the lysosomal degradation of AE2. Sci. Rep. 11, 5990. 10.1038/S41598-021-85499-6 33727633 PMC7966362

[B34] Ji W. Foo J. N. O'Roak B. J. Zhao H. Larson M. G. Simon D. B. (2008). Rare independent mutations in renal salt handling genes contribute to blood pressure variation. Nat. Genet. 40, 592–599. 10.1038/ng.118 18391953 PMC3766631

[B35] Jiang S. Wang X. M. Jin W. Zhang G. S. Zhang J. Xie P. (2019). NaHCO_3_ dilates mouse afferent arteriole via Na^+^/HCO_3_ ^−^ cotransporters NBCs. Hypertension 74, 1104–1112. 10.1161/Hypertensionaha.119.13235 31522618 PMC6785401

[B36] Kawaai K. Ando H. Satoh N. Yamada H. Ogawa N. Hirose M. (2017). Splicing variation of Long-IRBIT determines the target selectivity of IRBIT family proteins. Proc. Natl. Acad. Sci. USA. 114, 3921–3926. 10.1073/pnas.1618514114 28348216 PMC5393198

[B37] Kawaai K. Mizutani A. Shoji H. Ogawa N. Ebisui E. Kuroda Y. (2015). IRBIT regulates CaMKIIα activity and contributes to catecholamine homeostasis through tyrosine hydroxylase phosphorylation. Proc. Natl. Acad. Sci. USA. 112, 5515–5520. 10.1073/pnas.1503310112 25922519 PMC4418850

[B38] Khamaysi A. Anbtawee-Jomaa S. Fremder M. Eini-Rider H. Shimshilashvili L. Aharon S. (2019). Systemic succinate homeostasis and local succinate signaling affect blood pressure and modify risks for calcium oxalate lithogenesis. J. Am. Soc. Nephrol. 30, 381–392. 10.1681/ASN.2018030277 30728179 PMC6405146

[B39] Kikeri D. Azar S. Sun A. Zeidel M. L. Hebert S. C. (1990). Na^+^-H^+^ antiporter and Na^+^-(HCO_3_ ^−^)_n_ symporter regulate intracellular pH in mouse medullary thick limbs of Henle. Am。J. Physiol. 258, F445–F456. 10.1152/ajprenal.1990.258.3.F445 2156445

[B40] Kim Y. H. Kwon T. H. Christensen B. M. Nielsen J. Wall S. M. Madsen K. M. (2003). Altered expression of renal acid-base transporters in rats with lithium-induced NDI. Am. J. Physiol.-Renal Physiol. 285, F1244–F1257. 10.1152/ajprenal.00176.2003 12944321

[B41] Knauf F. Velazquez H. Pfann V. Jiang Z. Aronson P. S. (2019). Characterization of renal NaCl and oxalate transport in Slc26a6^-/-^ mice. Am. J. Physiol.-Renal Physiol. 316, F128–F133. 10.1152/ajprenal.00309.2018 30427220 PMC6383200

[B42] Knauf F. Yang C. L. Thomson R. B. Mentone S. A. Giebisch G. Aronson P. S. (2001). Identification of a chloride-formate exchanger expressed on the brush border membrane of renal proximal tubule cells. Proc. Natl. Acad. Sci. USA. 98, 9425–9430. 10.1073/pnas.141241098 11459928 PMC55437

[B43] Kokubo Y. Tomoike H. Tanaka C. Banno M. Okuda T. Inamoto N. (2006). Association of sixty-one non-synonymous polymorphisms in forty-one hypertension candidate genes with blood pressure variation and hypertension. Hypertens. Res. 29, 611–619. 10.1291/hypres.29.611 17137217

[B44] Lee H.-W. Handlogten M. E. Osis G. Clapp W. L. Wakefield D. N. Verlander J. W. (2017). Expression of sodium-dependent dicarboxylate transporter 1 (NaDC1/SLC13A2) in normal and neoplastic human kidney. Am. J. Physiol.-Renal Physiol. 312, F427–F435. 10.1152/ajprenal.00559.2016 27927654 PMC5374311

[B45] Lee S. K. Boron W. F. (2018). Exploring the autoinhibitory domain of the electrogenic Na^+^/HCO_3_ ^−^ transporter NBCe1-B, from residues 28 to 62. J. Physiol. 596, 3637–3653. 10.1113/JP276241 29808931 PMC6092277

[B46] Lee S. K. Boron W. F. Parker M. D. (2012). Relief of autoinhibition of the electrogenic Na-HCO_3_ cotransporter NBCe1-B: Role of IRBIT vs. amino-terminal truncation. Am.J. Physiol.-Cell Physiol. 302, C518–C526. 10.1152/ajpcell.00352.2011 22012331 PMC3287159

[B47] Liu Y. Wang D. K. Jiang D. Z. Qin X. Xie Z. D. Wang Q. K. (2013). Cloning and functional characterization of novel variants and tissue-specific expression of alternative amino and carboxyl termini of products of Slc4a10. PLoS One 8, e55974. 10.1371/journal.pone.0055974 23409100 PMC3567025

[B48] Liu Y. Yang J. Chen L. M. (2015). Structure and function of SLC4 family HCO_3_ ^−^ transporters. Front. Physiol. 6, 355. 10.3389/fphys.2015.00355 26648873 PMC4664831

[B49] Lu X. Wang L. Lin X. Huang J. Charles Gu C. He M. (2015). Genome-wide association study in Chinese identifies novel loci for blood pressure and hypertension. Hum. Mol. Genet. 24, 865–874. 10.1093/hmg/ddu478 25249183 PMC4303798

[B50] McAlear S. D. Liu X. Williams J. B. McNicholas-Bevensee C. M. Bevensee M. O. (2006). Electrogenic Na/HCO_3_ cotransporter (NBCe1) variants expressed in *Xenopus* oocytes: Functional comparison and roles of the amino and carboxy termini. J. Gen. Physiol. 127, 639–658. 10.1085/jgp.200609520 16735752 PMC2151535

[B51] Murthy M. Kurz T. O'Shaughnessy K. M. (2017). WNK signalling pathways in blood pressure regulation. Cell. Mol. Life Sci. 74, 1261–1280. 10.1007/s00018-016-2402-z 27815594 PMC5346417

[B52] Mutig K. (2017). Trafficking and regulation of the NKCC2 cotransporter in the thick ascending limb. Curr. Opin. Nephrol. Hypertens. 26, 392–397. 10.1097/Mnh.0000000000000351 28614115

[B53] Nwia S. M. Li X. C. Leite A. P. d. O. Hassan R. Zhuo J. L. (2022). The Na^+^/H^+^ exchanger 3 in the intestines and the proximal tubule of the kidney: Localization, physiological function, and key roles in angiotensin II-induced hypertension. Front. Physiol. 13, 861659. 10.3389/fphys.2022.861659 35514347 PMC9062697

[B54] Odgaard E. Jakobsen J. K. Frische S. Praetorius J. Nielsen S. Aalkjær C. (2004). Basolateral Na^+^-dependent HCO_3_ ^−^ transporter NBCn1-mediated HCO_3_ ^−^ influx in rat medullary thick ascending limb. J. Physiol. 555, 205–218. 10.1113/jphysiol.2003.046474 14673192 PMC1664813

[B55] Ohana E. Shcheynikov N. Moe O. W. Muallem S. (2013). SLC26A6 and NaDC-1 transporters interact to regulate oxalate and citrate homeostasis. J. Am. Soc. Nephrol. 24, 1617–1626. 10.1681/Asn.2013010080 23833257 PMC3785279

[B56] Park S. Shcheynikov N. Hong J. H. Zheng C. Suh S. H. Kawaai K. (2013). IRBIT mediates synergy between Ca^2+^ and cAMP signaling pathways during epithelial transport in mice. Gastroenterology 145, 232–241. 10.1053/j.gastro.2013.03.047 23542070 PMC3696401

[B57] Parker M. D. Boron W. F. (2013). The divergence, actions, roles, and relatives of sodium coupled bicarbonate transporters. Physiol. Rev. 93, 803–959. 10.1152/physrev.00023.2012 23589833 PMC3768104

[B58] Penton D. Moser S. Wengi A. Czogalla J. Rosenbaek L. L. Rigendinger F. (2019). Protein phosphatase 1 inhibitor-1 mediates the cAMP-dependent stimulation of the renal NaCl cotransporter. J. Am. Soc. Nephrol. 30, 737–750. 10.1681/asn.2018050540 30902838 PMC6493980

[B59] Peti-Peterdi J. Harris R. C. (2010). Macula densa sensing and signaling mechanisms of renin release. J. Am. Soc. Nephrol. 21, 1093–1096. 10.1681/Asn.2009070759 20360309 PMC4577295

[B60] Picard N. Trompf K. Yang C. L. Miller R. L. Carrel M. Loffing-Cueni D. (2014). Protein phosphatase 1 inhibitor-1 deficiency reduces phosphorylation of renal NaCl cotransporter and causes arterial hypotension. J. Am. Soc. Nephrol. 25, 511–522. 10.1681/Asn.2012121202 24231659 PMC3935578

[B61] Poulsen S. B. Cheng L. Penton D. Kortenoeven M. L. A. Matchkov V. V. Loffing J. (2021). Activation of the kidney sodium chloride cotransporter by the beta 2-adrenergic receptor agonist salbutamol increases blood pressure. Kidney Intl 100, 321–335. 10.1016/j.kint.2021.04.021 33940111

[B62] Praetorius J. Kim Y. H. Bouzinova E. V. Frische S. Rojek A. Aalkjaer A. (2004). NBCn1 is a basolateral Na^+^-HCO_3_ ^−^ cotransporter in rat kidney inner medullary collecting ducts. Am. J. Physiol.-Renal Physiol. 286, F903–F912. 10.1152/ajprenal.00437.2002 15075186

[B63] Quentin F. Eladari D. Frische S. Cambillau M. Nielsen S. Alper S.L. (2004). Regulation of the Cl^−^/HCO_3_ ^−^ exchanger AE2 in rat thick ascending limb of Henle's loop in response to changes in acid-base and sodium balance. J. Am. Soc. Nephrol. 15, 2988–2997. 10.1097/01.ASN.0000146426.93319.16 15579501

[B64] Richardson C. Alessi D. R. (2008). The regulation of salt transport and blood pressure by the WNK-SPAK/OSR1 signalling pathway. J. Cell Sci. 121, 3293–3304. 10.1242/jcs.029223 18843116

[B65] Shao S. Li X.-D. Lu Y.-Y. Li S.-J. Chen X.-H. Zhou H.-D. (2021). Renal natriuretic peptide receptor-C deficiency attenuates NaCl cotransporter activity in angiotensin II-induced hypertension. Hypertension 77, 868–881. 10.1161/hypertensionaha.120.15636 33486984

[B66] Shekarabi M. Zhang J. Khanna A. R. Ellison D. H. Delpire E. Kahle K. T. (2017). WNK kinase signaling in ion homeostasis and human disease. Cell Metab. 25, 285–299. 10.1016/j.cmet.2017.01.007 28178566

[B67] Shirakabe K. Priori G. Yamada H. Ando H. Horita S. Fujita T. (2006). IRBIT, an inositol 1,4,5-trisphosphate receptor-binding protein, specifically binds to and activates pancreas-type Na^+^/HCO_3_ ^–^ cotransporter 1 (pNBC1). Proc. Natl. Acad. Sci. USA. 103, 9542–9547. 10.1073/pnas.0602250103 16769890 PMC1480443

[B68] Sober S. Org E. Kepp K. Juhanson P. Eyheramendy S. Gieger C. (2009). Targeting 160 candidate genes for blood pressure regulation with a genome-wide genotyping array. PLoS One 4, e6034. 10.1371/journal.pone.0006034 19562039 PMC2699027

[B69] Su P. Wu H. Wang M. Cai L. Liu Y. Chen L. M. (2021). IRBIT activates NBCe1-B by releasing the auto-inhibition module from the transmembrane domain. J. Physiol. 599, 1151–1172. 10.1113/JP280578 33237573 PMC7898672

[B70] Torres-Pinzon D. L. Ralph D. L. Veiras L. C. McDonough A. A. (2021). Sex-specific adaptations to high-salt diet preserve electrolyte homeostasis with distinct sodium transporter profiles. Am. J. Physiol-Cell Physiol. 321, C897–C909. 10.1152/ajpcell.00282.2021 34613843 PMC8616593

[B71] Tran T. M. Park M. Y. Lee J. Bae J. S. Hwang S. M. Choi S. Y. (2013). IRBIT plays an important role in NHE3-mediated pH_i_ regulation in HSG cells. Biochem. Biophys. Res. Commun. 437, 18–22. 10.1016/j.bbrc.2013.06.010 23769829

[B72] Trudeau M. C. Warmke J. W. Ganetzky B. Robertson G. A. (1995). HERG, a human inward rectifier in the voltage-gated potassium channel family. Science 269, 92–95. 10.1126/science.7604285 7604285

[B73] Wadei H. M. Textor S. C. (2012). The role of the kidney in regulating arterial blood pressure. Nat. Rev. Nephrol. 8, 602–609. 10.1038/nrneph.2012.191 22926246

[B74] Wang J. L. Wang X. Y. Wang D. K. Parker M. D. Musa-Aziz R. Popple J. (2020a). Multiple acid-base and electrolyte disturbances upregulate NBCn1, NBCn2, IRBIT and L-IRBIT in the mTAL. J. Physiol. 598, 3395–3415. 10.1113/JP279009 32359081 PMC8403746

[B75] Wang L. Li H. Yang B. Guo L. Han X. Li L. (2017). The hypertension risk variant rs820430 functions as an enhancer of SLC4A7. Am. J. Hyperten. 30, 202–208. 10.1093/ajh/hpw127 27784683

[B76] Wang M. Wu H. Liu Y. Chen L.-M. (2020b). Activation of mouse NBCe1-B by *Xenopus* laevis and mouse IRBITs: Role of the variable Nt appendage of IRBITs. BBA-Biomembranes. 1862, 183240. 10.1016/j.bbamem.2020.183240 32119862

[B77] Wetzel R. K. Sweadner K. J. (2001). Immunocytochemical localization of Na-K-ATPase alpha- and gamma-subunits in rat kidney. Am. J. Physiol.-Renal Physiol. 281, F531–F545. 10.1152/ajprenal.2001.281.3.F531 11502602

[B78] Yamaguchi S. Ishikawa T. (2014). AHCYL2 (long-IRBIT) as a potential regulator of the electrogenic Na^+^-HCO_3_ ^−^ cotransporter NBCe1-B. FEBS Lett. 588, 672–677. 10.1016/j.febslet.2013.12.036 24472682

[B79] Yang H. C. Liang Y. J. Chen J. W. Chiang K. M. Chung C. M. Ho H. Y. (2012). Identification of IGF1, SLC4A4, WWOX, and SFMBT1 as hypertension susceptibility genes in Han Chinese with a genome-wide gene-based association study. PLoS One 7, e32907. 10.1371/journal.pone.0032907 22479346 PMC3315540

