# Supplementary material for: Dietary sodium enhances the expression of SLC4 family transporters, IRBIT, L-IRBIT, and PP1 in rat kidney: Insights into the molecular mechanism for renal sodium handling
Source: Front Physiol. 2023 Apr 4;14:1154694. doi: 10.3389/fphys.2023.1154694 (PMC10111226; doi:10.3389/fphys.2023.1154694)
Supplement: Supplementary file 1 [file DataSheet3.PDF]

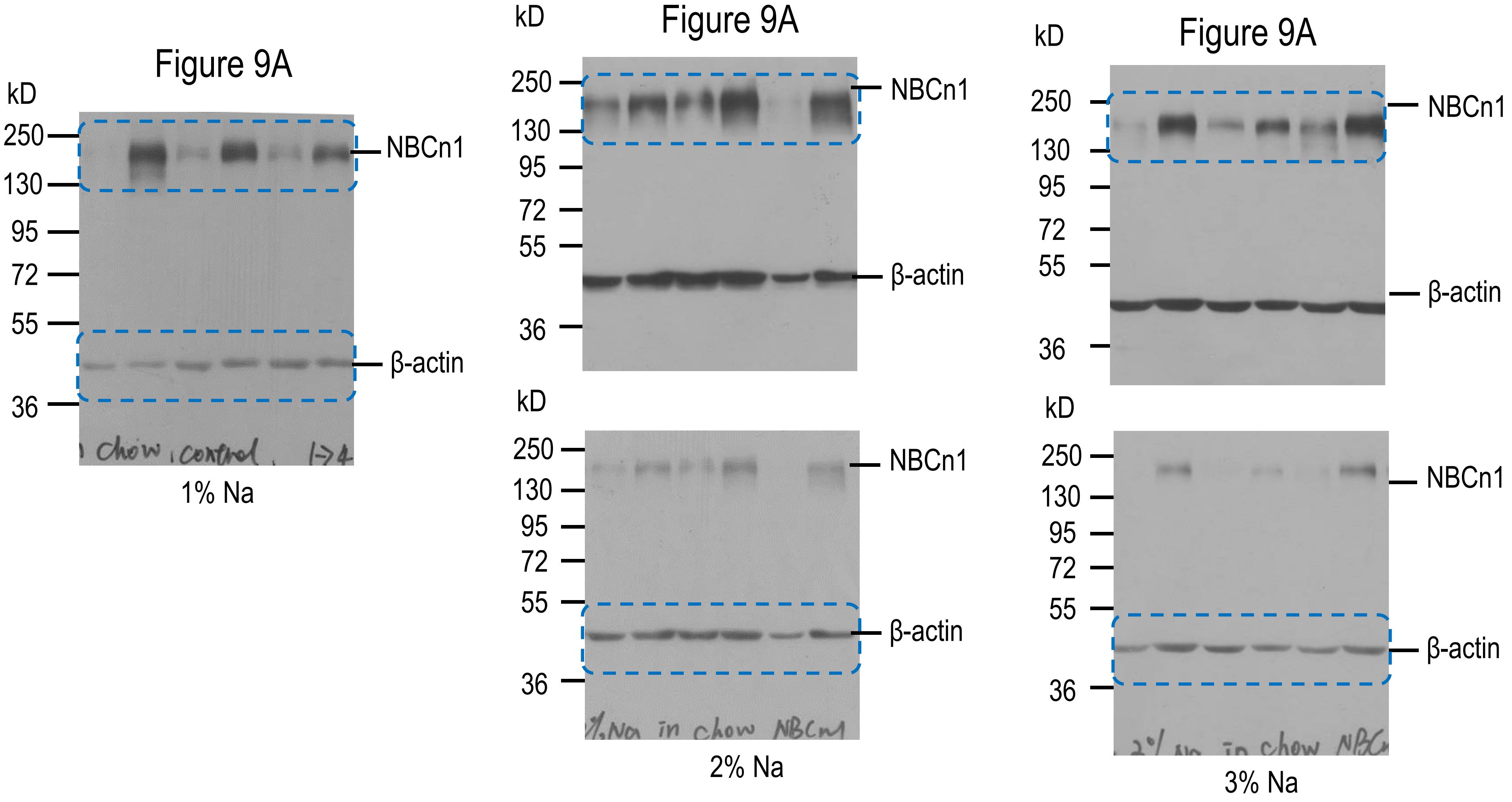

The dashed boxes indicate the bands shown in the figures in the manuscript.

Figure 9C

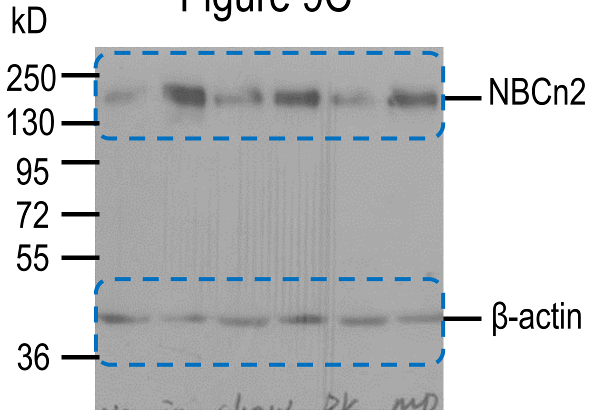

1% Na

Figure 9C

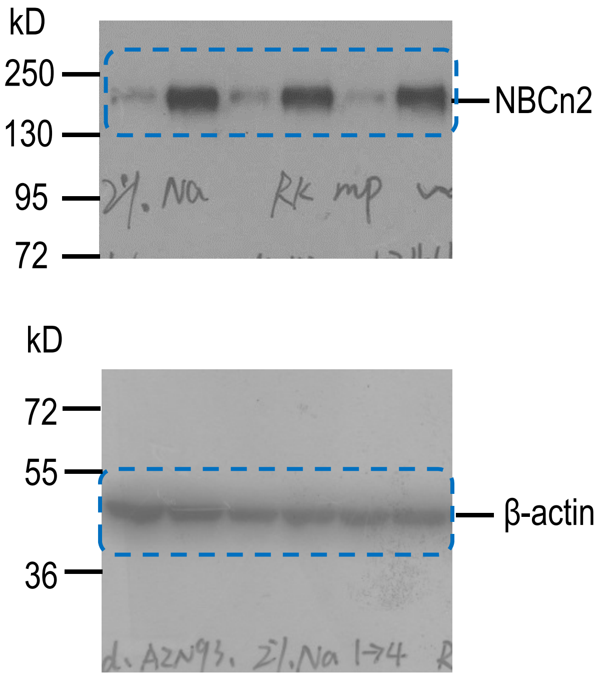

2% Na

Figure 9C

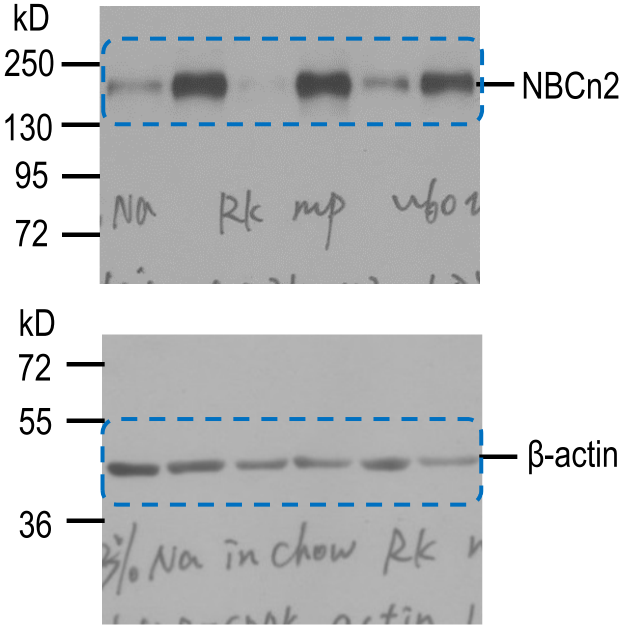

3% Na

The blot was cut into two halves. The upper half was probed for NBCn2, and the lower half was probed with actin.

Figure 9E

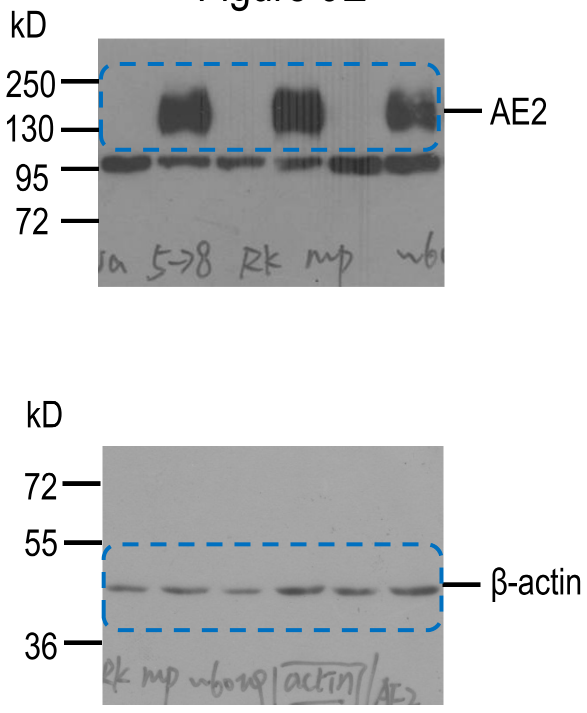

1% Na

Figure 9E

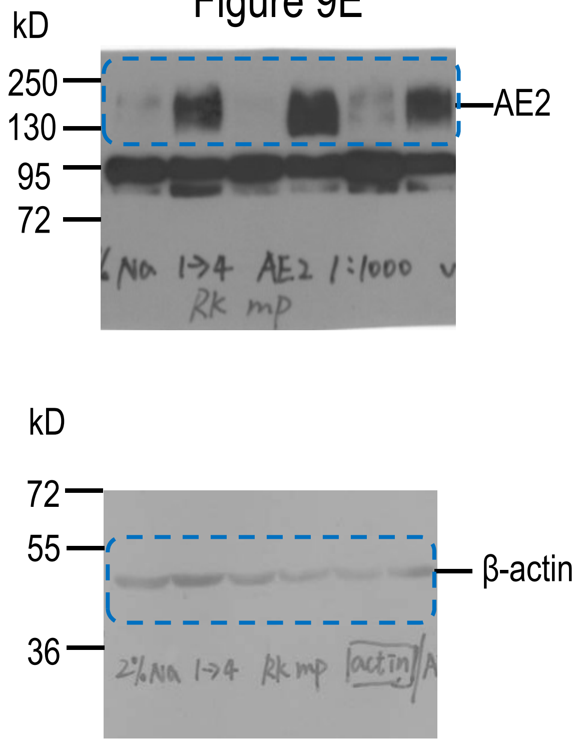

2% Na

Figure 9E

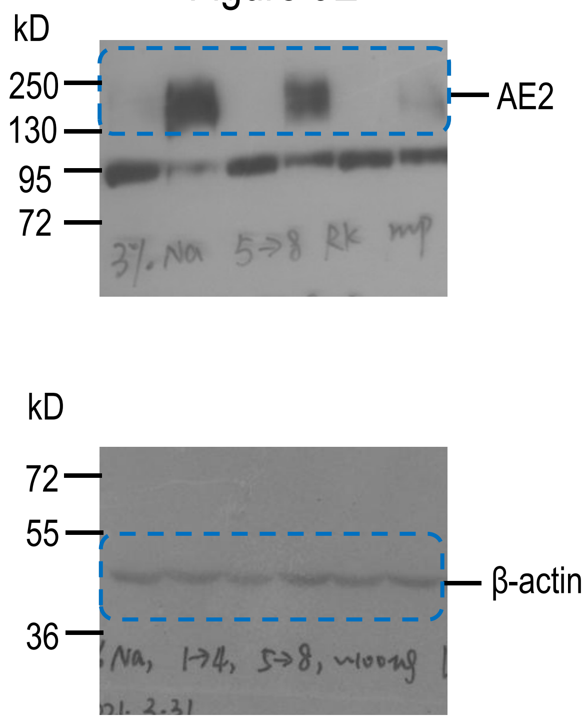

3% Na

↑ ↑ ↑

The blots were cut into two halves. The upper halves were probed for AE2, and the lower halves were probed with actin.

Figure 9F

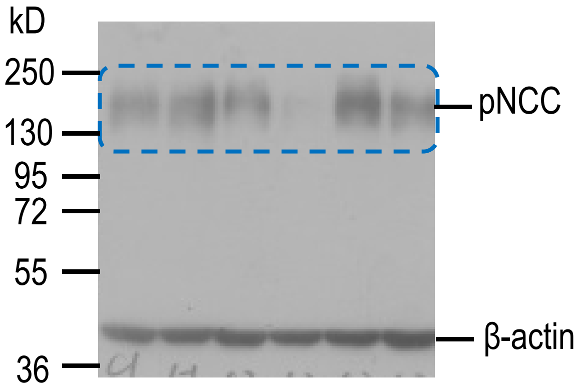

Figure 9F

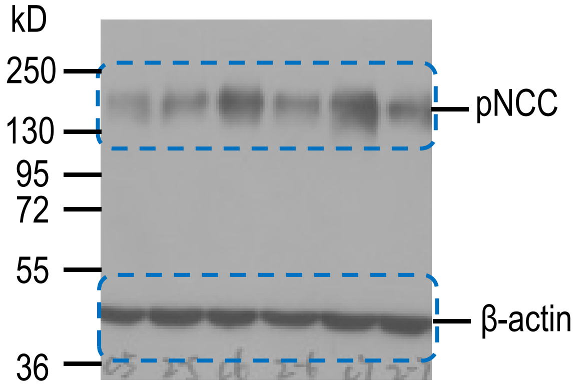

2% Na

Figure 9F

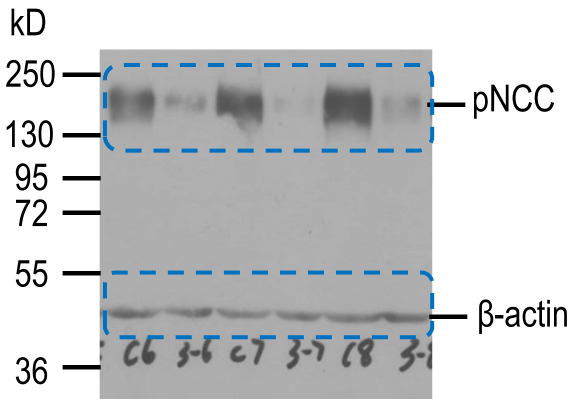

3% Na

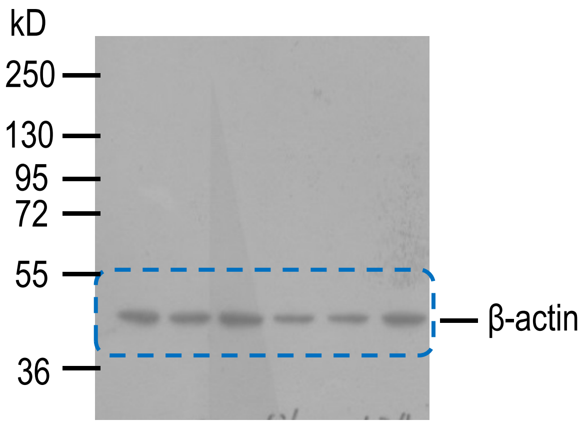

1% Na

Figure 10A

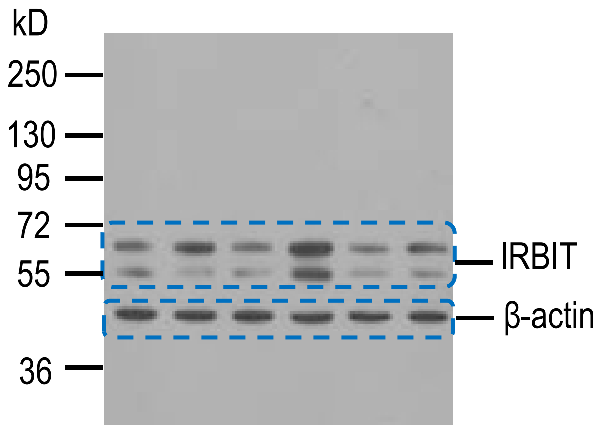

1% Na

Figure 10A

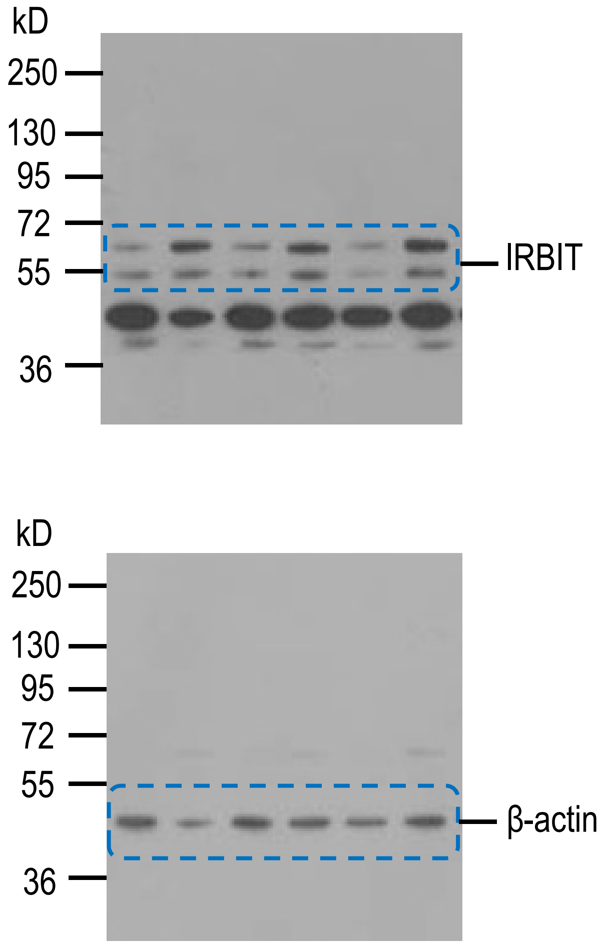

2% Na

Figure 10A

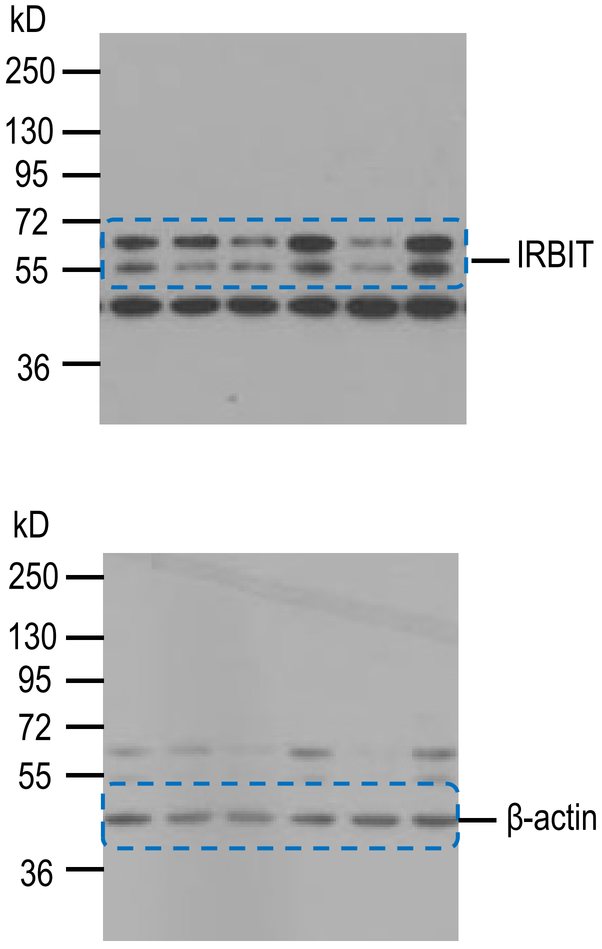

3% Na

Figure 10C

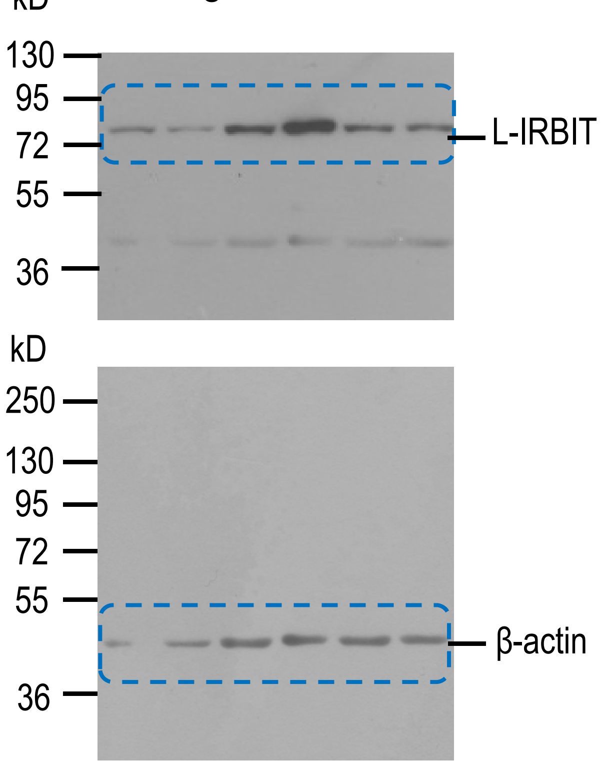

1% Na

Figure 10C

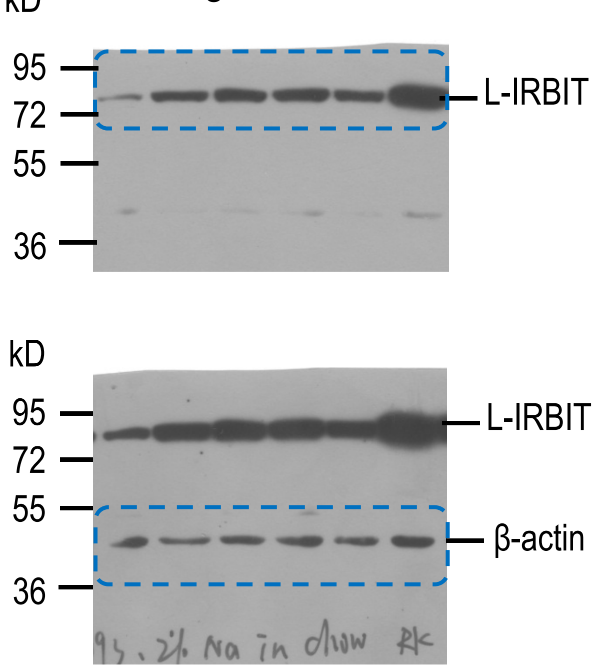

2% Na

Figure 10C

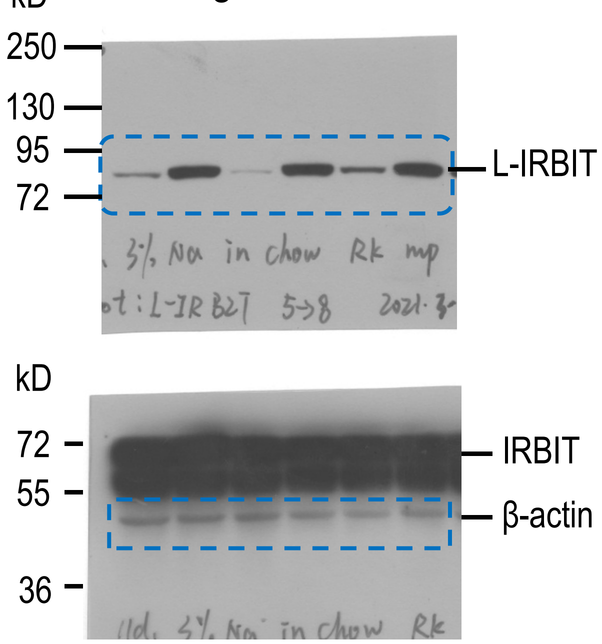

3% Na

The blot was cut into two halves. The upper half was probed with L-IRBIT. The lower half was probed with IRBIT and actin.

Figure 10E

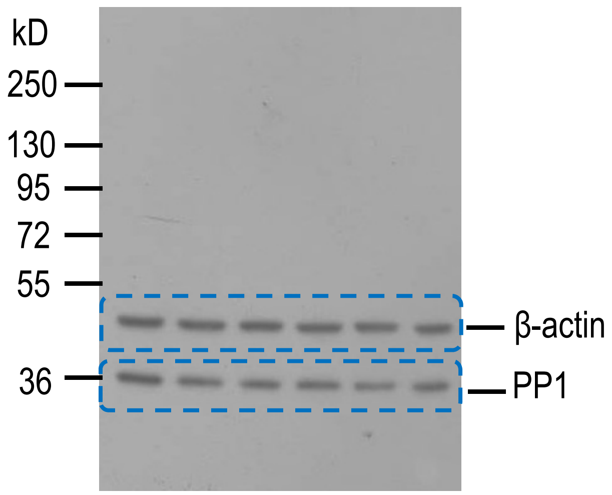

1% Na

Figure 10E

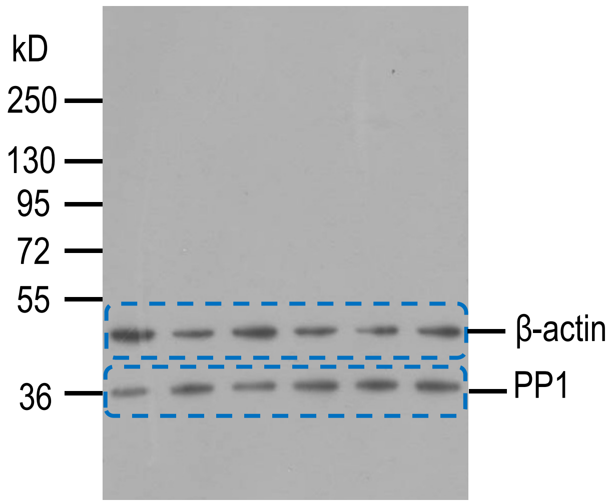

2% Na

Figure 10E

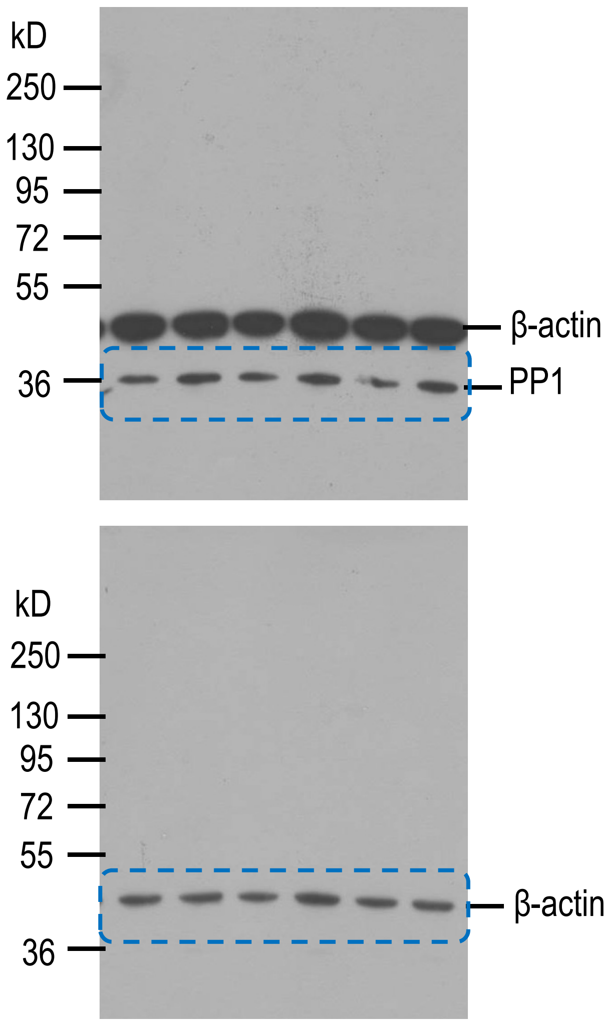

3% Na

Figure 11E

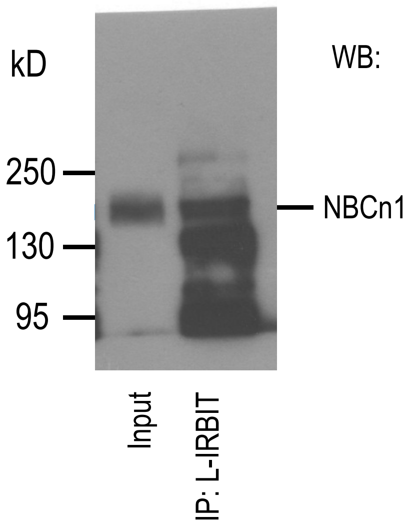

Figure 11E

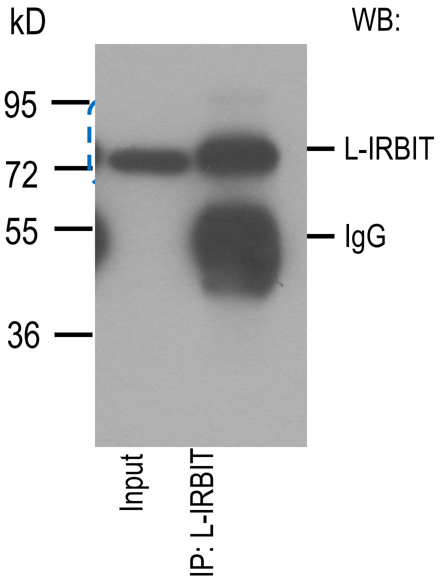

The blot was cut into two halves. The upper half was probed with NBCn1. The lower half was probed with L-IRBIT
