# Supplementary material for: Dietary sodium enhances the expression of SLC4 family transporters, IRBIT, L-IRBIT, and PP1 in rat kidney: Insights into the molecular mechanism for renal sodium handling
Source: Front Physiol. 2023 Apr 4;14:1154694. doi: 10.3389/fphys.2023.1154694 (PMC10111226; doi:10.3389/fphys.2023.1154694)
Supplement: Supplementary file 2 [file DataSheet1.PDF]

Supplementary Material

Dietary sodium enhances the expression of SLC4 family transporters, IRBIT, L-IRBIT, and PP1 in rat kidney: insights into the molecular mechanism for renal sodium handling

Lu Cai[1], Dengke Wang[2], Tianxiang Gui[1], Xiaoyu Wang[1], Lingyu Zhao[1], Walter F. Boron[2], Li-Ming Chen[1]*, Ying Liu[1]*

[1]Key Laboratory of Molecular Biophysics of Ministry of Education, School of Life Science & Technology, Huazhong University of Science & Technology, Wuhan, Hubei 430074, China
[2]Department of Physiology and Biophysics, Case Western Reserve University School of Medicine, Cleveland, OH 44106, USA

*** Correspondence:**

Ying Liu: liuying@hust.edu.cn

Li-Ming Chen: liming.chen@hust.edu.cn

1 Supplementary Figures and Tables

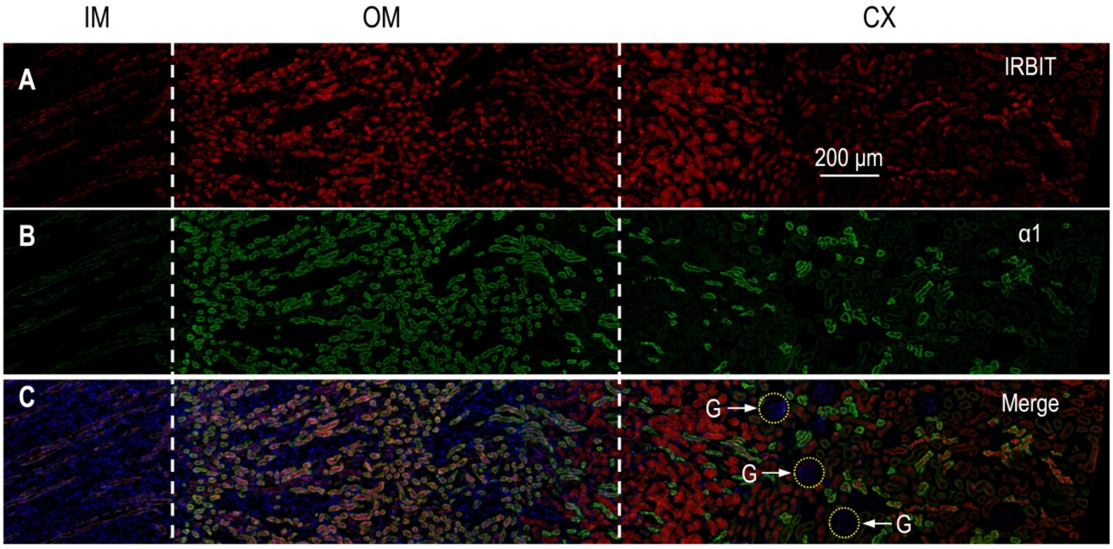

Supplementary Figure S1. Overview of double-staining of IRBIT and α1 of Na^{+}-K^{+} ATPase in rat kidney. (A) Distribution of immunofluorescence signals of IRBIT in the kidney. **(B)** Distribution of immunofluorescence signals of α1 in the kidney. **(C)** Merge of IRBIT and α1. IM: inner medulla. OM: outer medulla. CX: cortex. G: glomerulus. Blue indicates the nuclei counter-stained with DAPI.

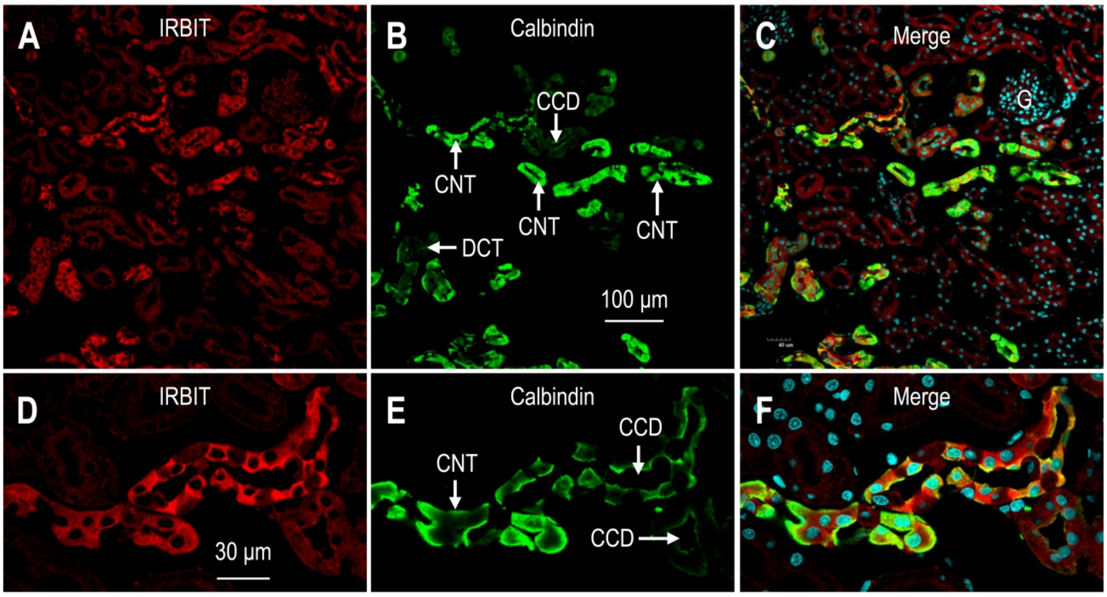

Supplementary Figure S2. Indirect immunofluorescence microscopy of IRBIT and Calbindin in the cortex of rat kidney. (A−C) Double staining of IRBIT and calbindin in renal cortex. **(D−F)** High-magnification view showing the immunofluorescence localization of IRBIT and calbindin in the cortical tubules. The structures strongly labeled by calbindin represent the cortical connecting tubule (CNT). The structures with relatively lower levels of calbindin signals presumably represent cortical collecting duct (CCD) or distal convoluted tubules (DCT). G: glomerulus.

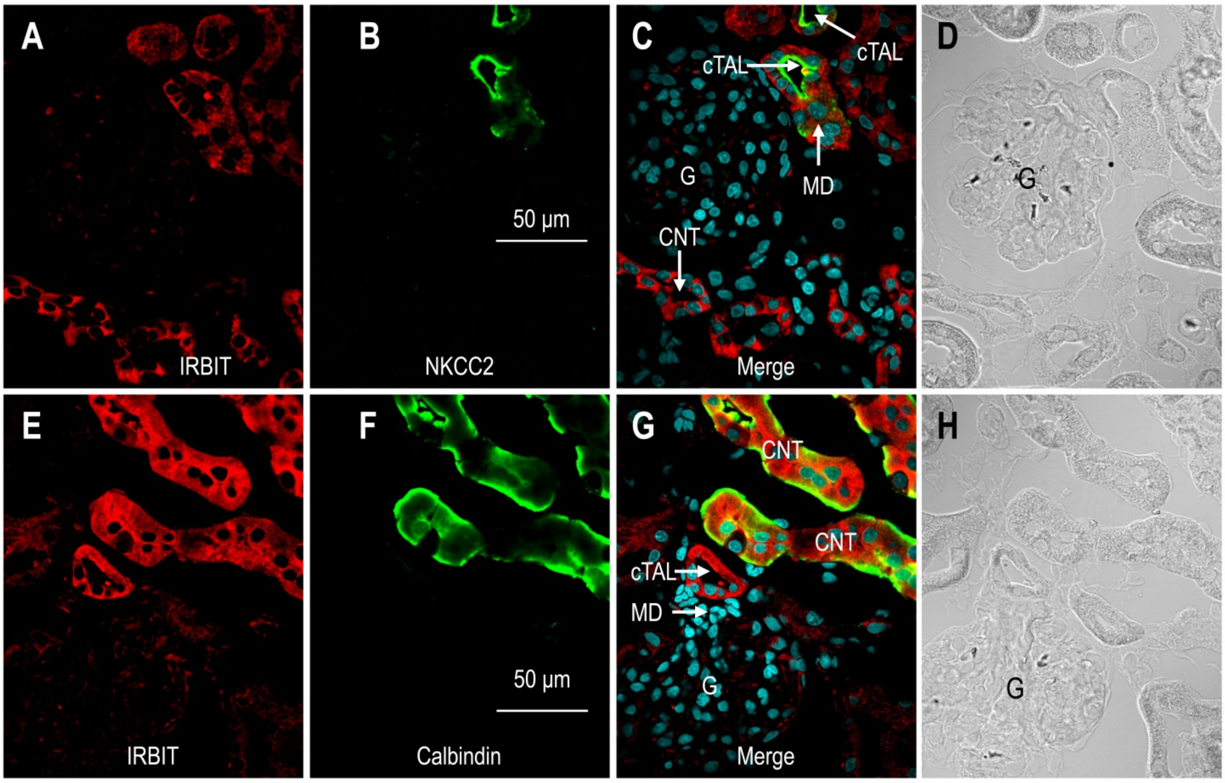

Supplementary Figure S3. Localization of IRBIT by immunofluorescence microscopy in renal cortex of rat. (A−C) Double-staining of IRBIT (red) and NKCC2 (green) in the cortex. **(D)** Light-field view of the same section shown in panels **A−C**. **(E−G)** Double-staining of IRBIT (red) and calbindin (green) in the cortex. **(H)** Light-field view of the same section shown in panels **E−G**. In panels **A−C**, the structures stained with IRBIT but not NKCC2 presumably represent cortical connecting tubules (CNT), whereas the juxtaglomerular structure with both IRBIT and NKCC2 represents the cortical thick ascending limb (cTAL). In panel G, the juxtaglomerular structure with with IRBIT but calbindin represents the cTAL, whereas the structures labeled by both IRBIT and calbindin represent the CNTs. G: glomerulus. MD: macula densa.

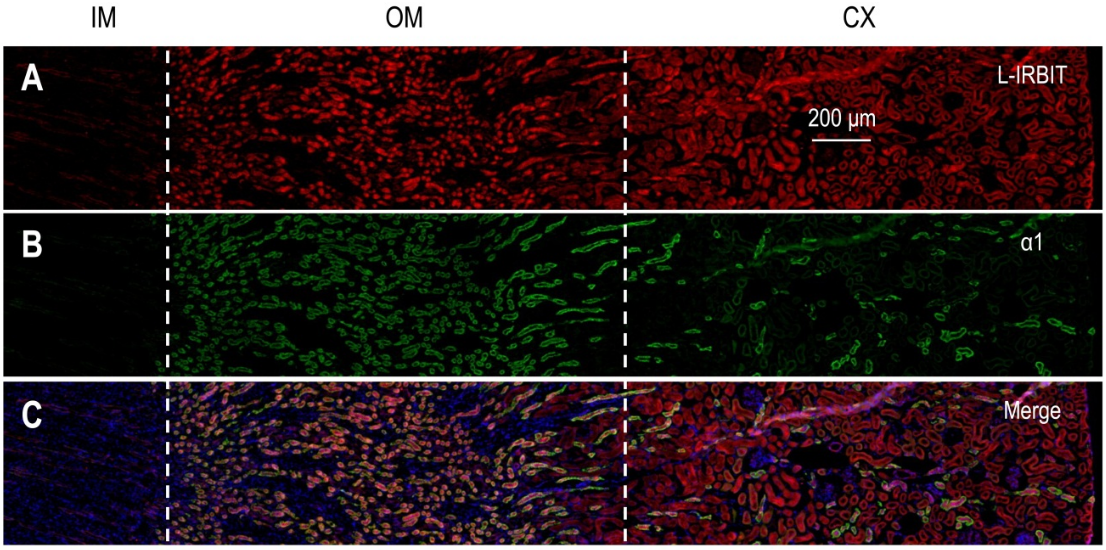

Supplementary Figure S4. Overview of double-staining of L-IRBIT and α1 in rat kidney. (A) Distribution of immunofluorescence signals of L-IRBIT. **(B)** Distribution of immunofluorescence signals of α1. **(C)** Merge of L-IRBIT and α1. IM: inner medulla. OM: outer medulla. CX: cortex. In panel C, the blue indicates the nuclei counter-stained by DAPI.

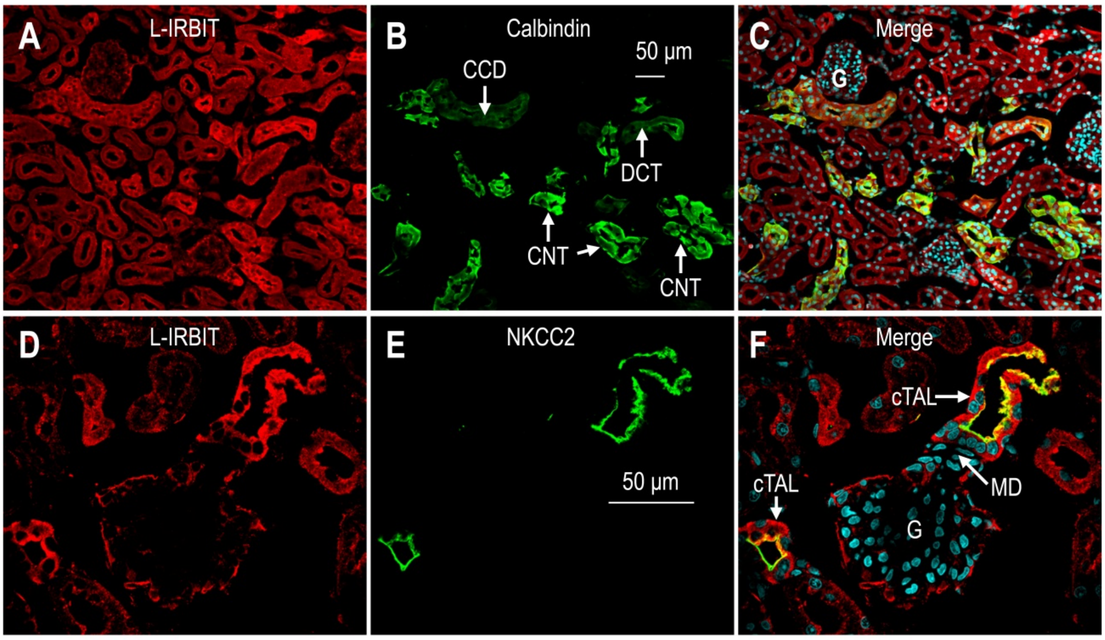

Supplementary Figure S5. Localization of L-IRBIT by immunofluorescence microscopy in renal cortex of rat. (A−B) Double-staining of L-IRBIT (red) and calbindin (green) in the cortex. The structures strongly labeled by calbindin represent cortical connecting duct (CNT). The structures with relatively lower levels of calbindin are presumably cortical collecting duct (CCD) or distal convoluted tubule (DCT). **(D−F)** High magnification view showing the double-staining of L-IRBIT and NKCC2 in the cortex. The juxtaglomerular tubules labeled by both L-IRBIT and NKCC2 represent the cortical thick ascending limb (cTAL). MD: macula densa. G: glomerulus. Blue indicates the nuclei counter-stained by DAPI.

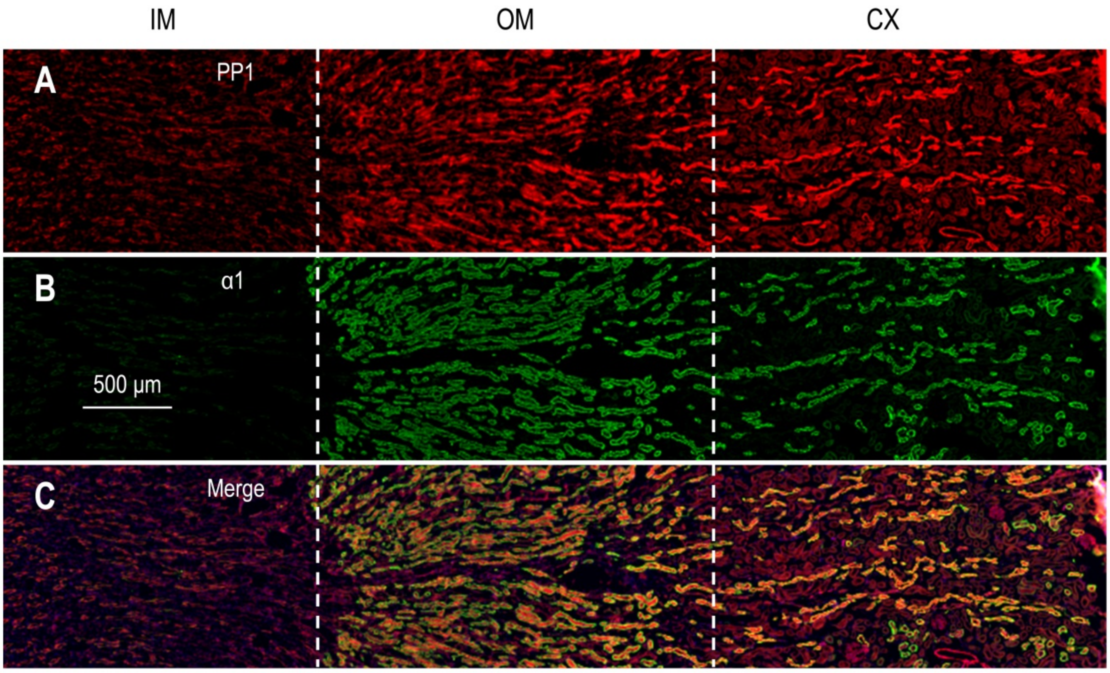

Supplementary Figure S6. Overview of double-staining of PP1 and α1 in rat kidney. (A−C) Distribution of immunofluorescence signals of PP1 in the kidney. (B) Distribution of immunofluorescence of α1 in the kidney. (C) Merge of PP1 and α1. IM: inner medulla. OM: outer medulla. CX: Cortex. In panel C, the blue indicates the nuclei counter-stained by DAPI.

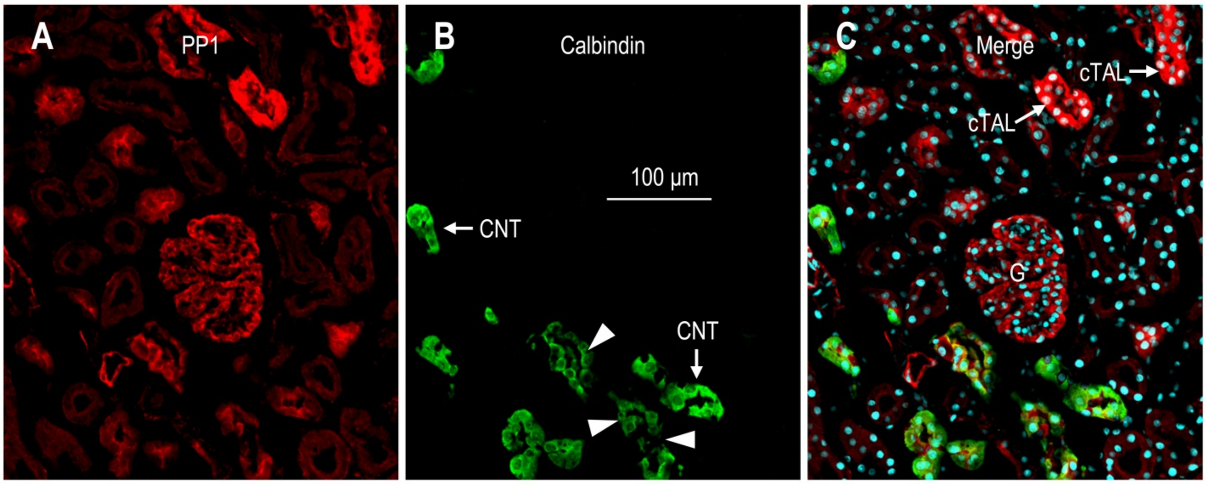

Supplementary Figure S7. Immunofluorescence double-staining of PP1 and calbindin in rat kidney. (A) Localization of immunofluorescence signals of PP1 in the cortex. (B) Localization of immunofluorescence signals of calbindin in the cortex. (C) Merge of PP1 and α1. The structures strongly labeled by calbindin represent cortical connecting tubule (CNT). The structures with relatively lower level of calbindin (indicated by arrow heads) are presumably cortical collecting duct (CCD) or distal convoluted tubule (DCT). The structures labeled by PP1 but not calbindin represent cortical thick ascending limb (cTAL). G, glomerulus.
