# Supplementary figures and images for: Dietary sodium enhances the expression of SLC4 family transporters, IRBIT, L-IRBIT, and PP1 in rat kidney: Insights into the molecular mechanism for renal sodium handling
Source: Front Physiol. 2023 Apr 4;14:1154694. doi: 10.3389/fphys.2023.1154694 (PMC10111226; doi:10.3389/fphys.2023.1154694)

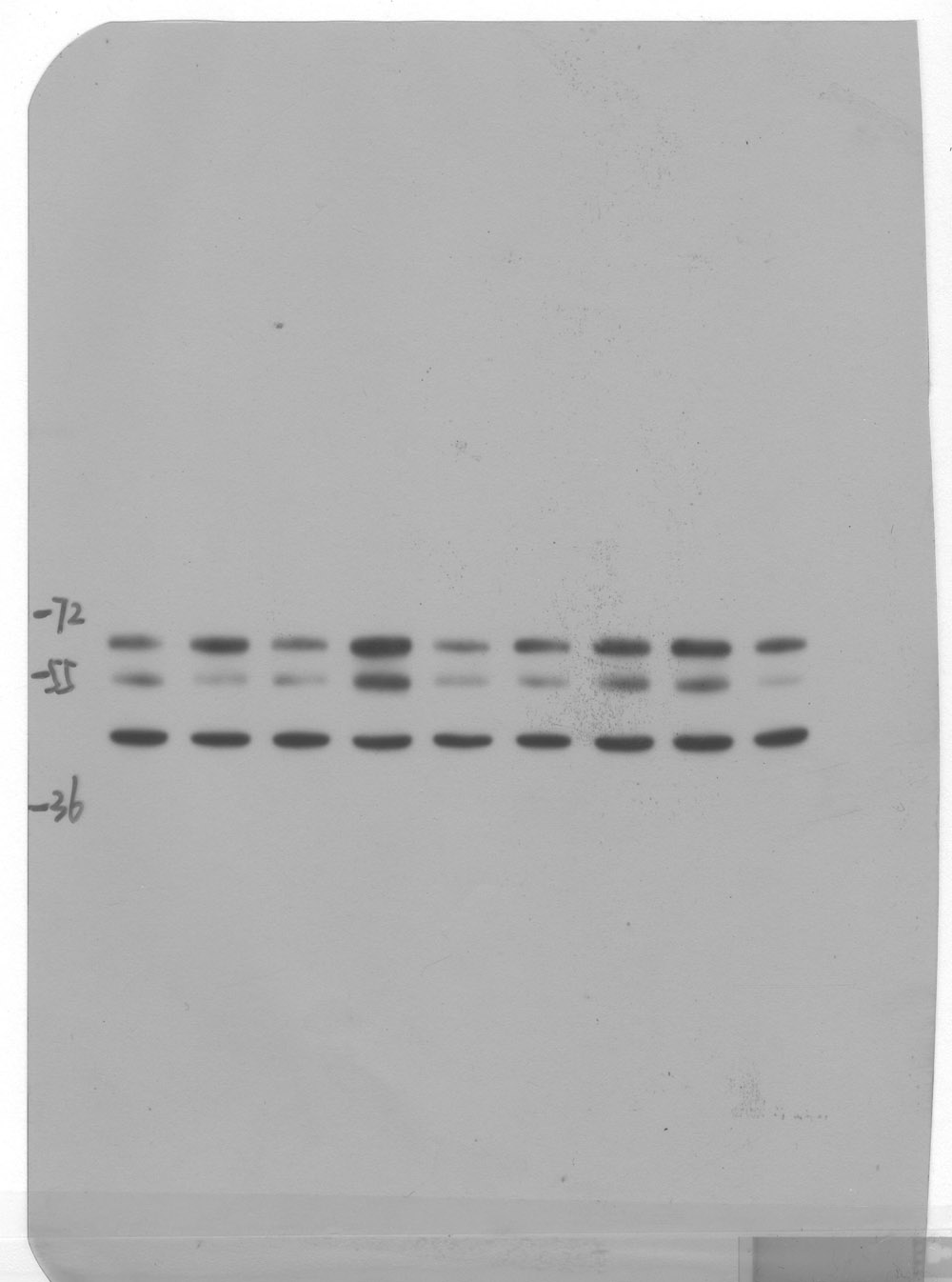

Supplement: Supplementary file 3 [file DataSheet2.ZIP › Small sized/Figure 10A-1% Na-IRBIT+actin.jpg]

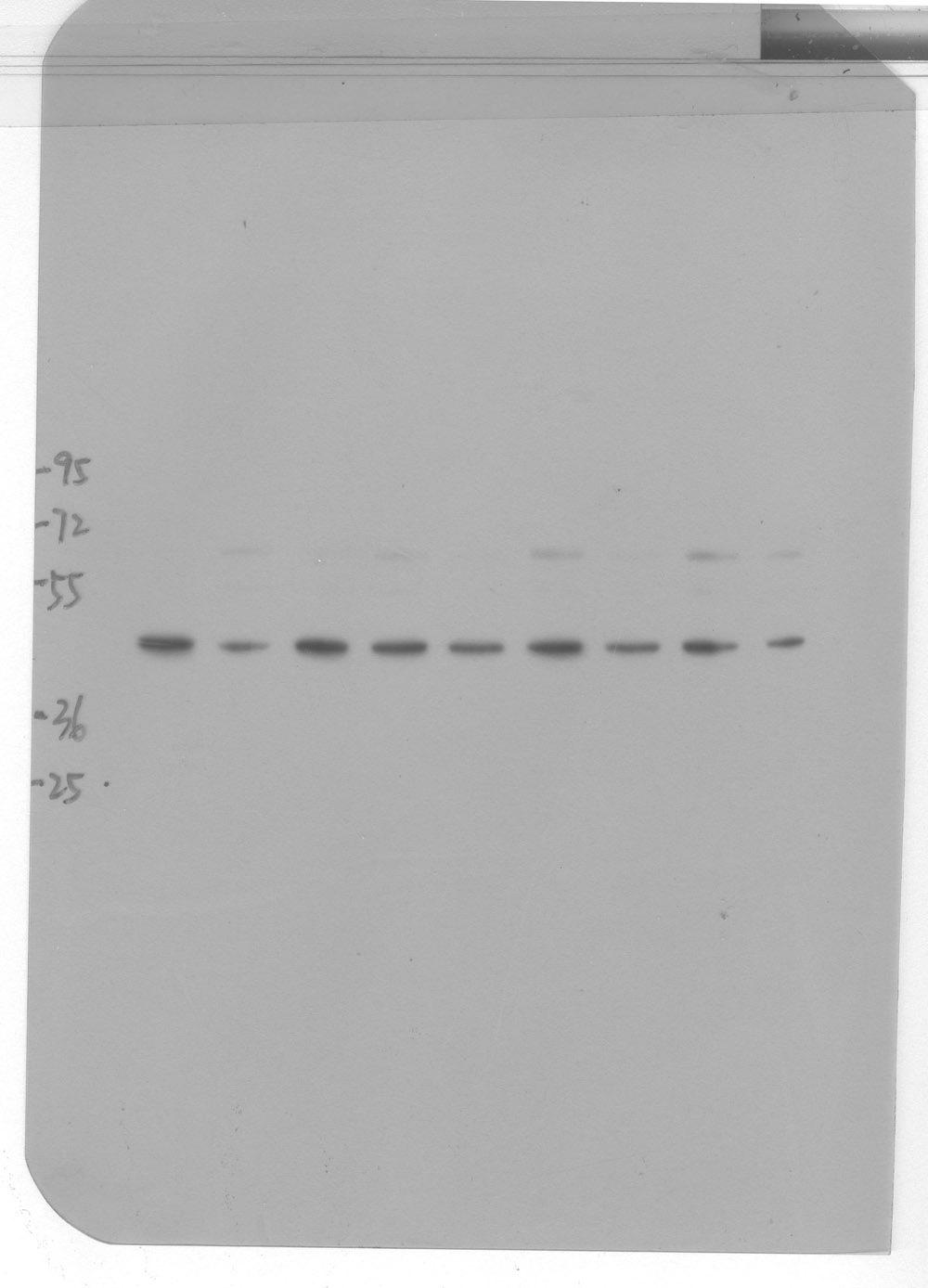

Supplement: Supplementary file 3 [file DataSheet2.ZIP › Small sized/Figure 10A-2% Na-Actin.jpg]

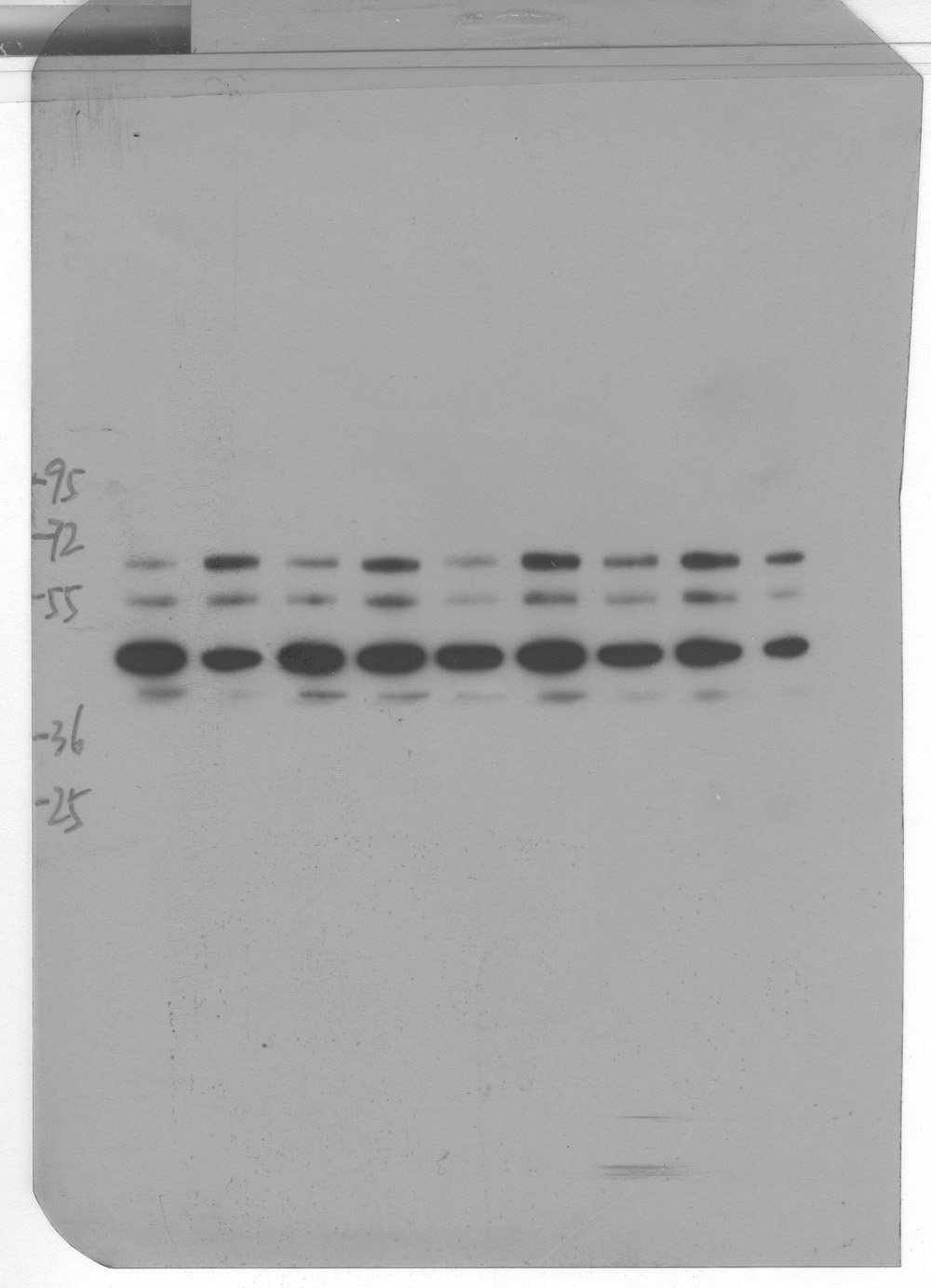

Supplement: Supplementary file 3 [file DataSheet2.ZIP › Small sized/Figure 10A-2% Na-IRBIT.jpg]

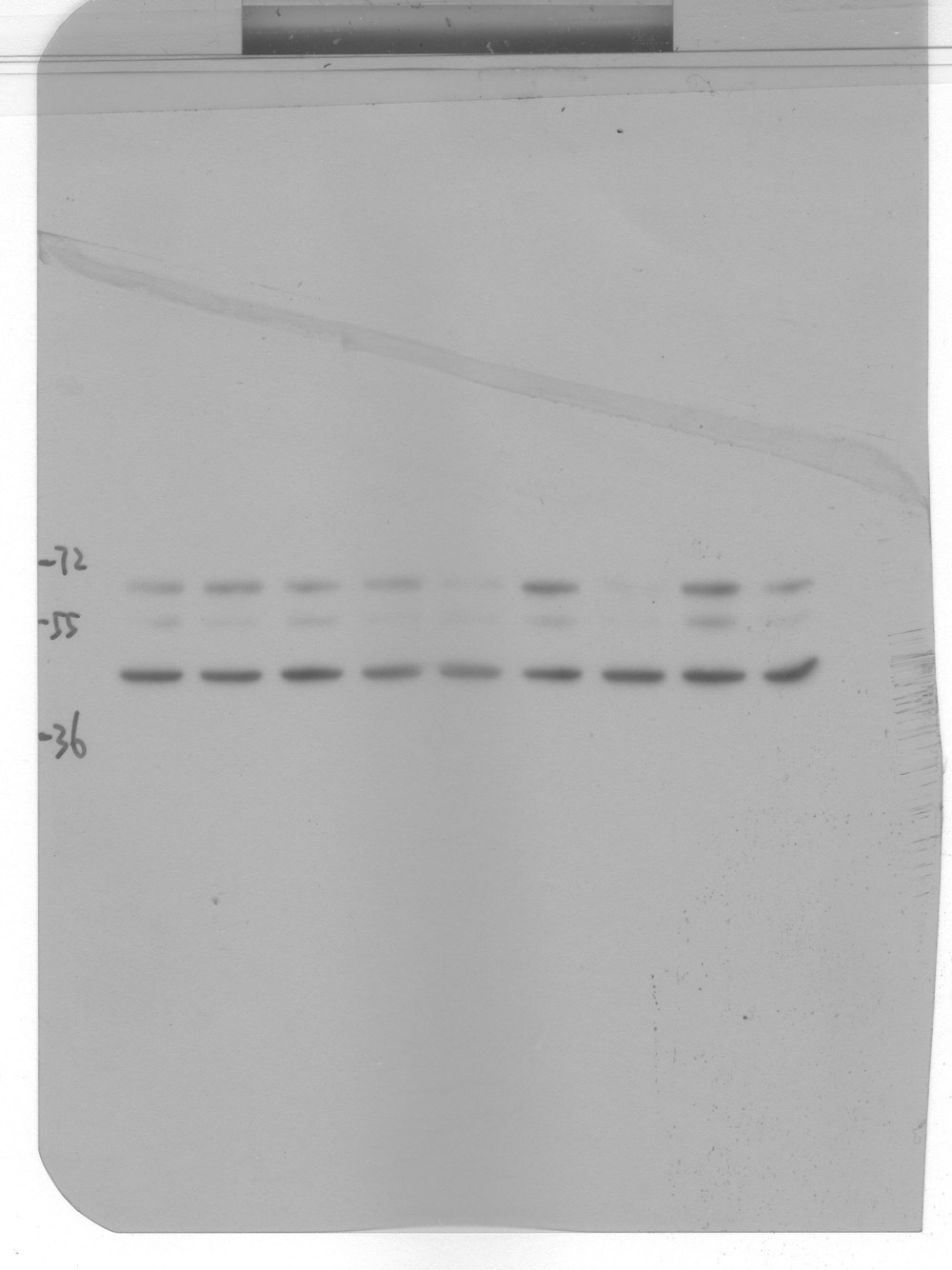

Supplement: Supplementary file 3 [file DataSheet2.ZIP › Small sized/Figure 10A-3% Na-Actin.jpg]

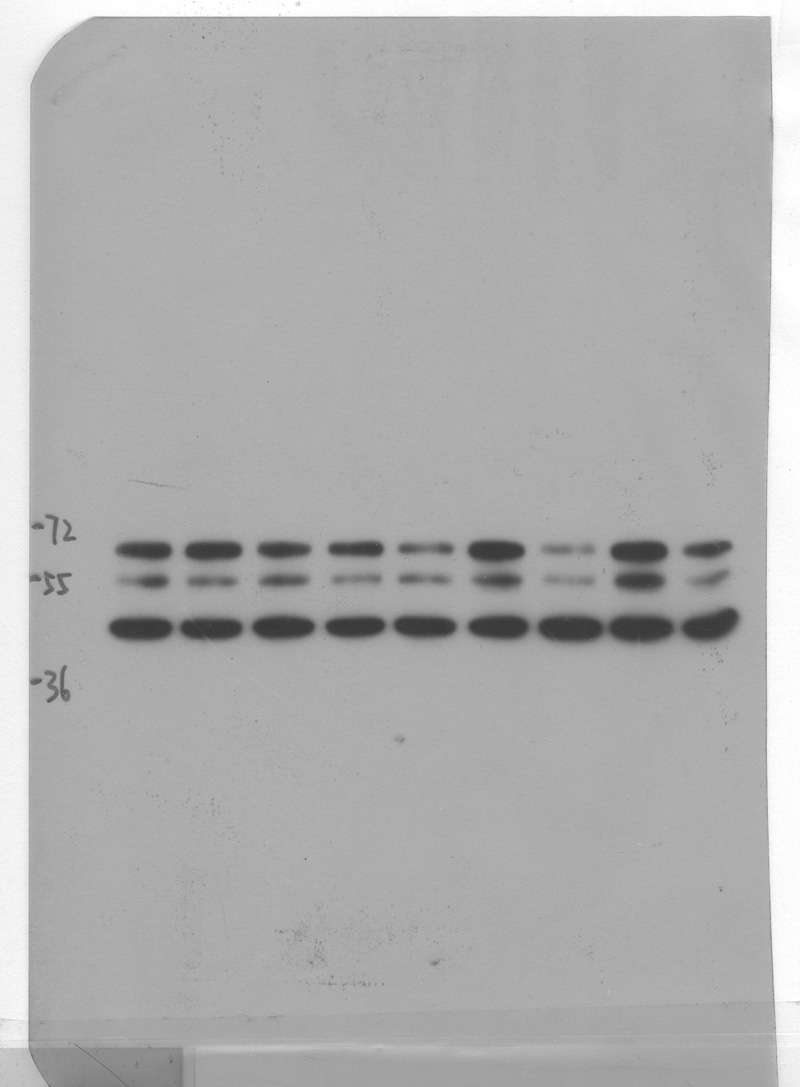

Supplement: Supplementary file 3 [file DataSheet2.ZIP › Small sized/Figure 10A-3% Na-IRBIT.jpg]

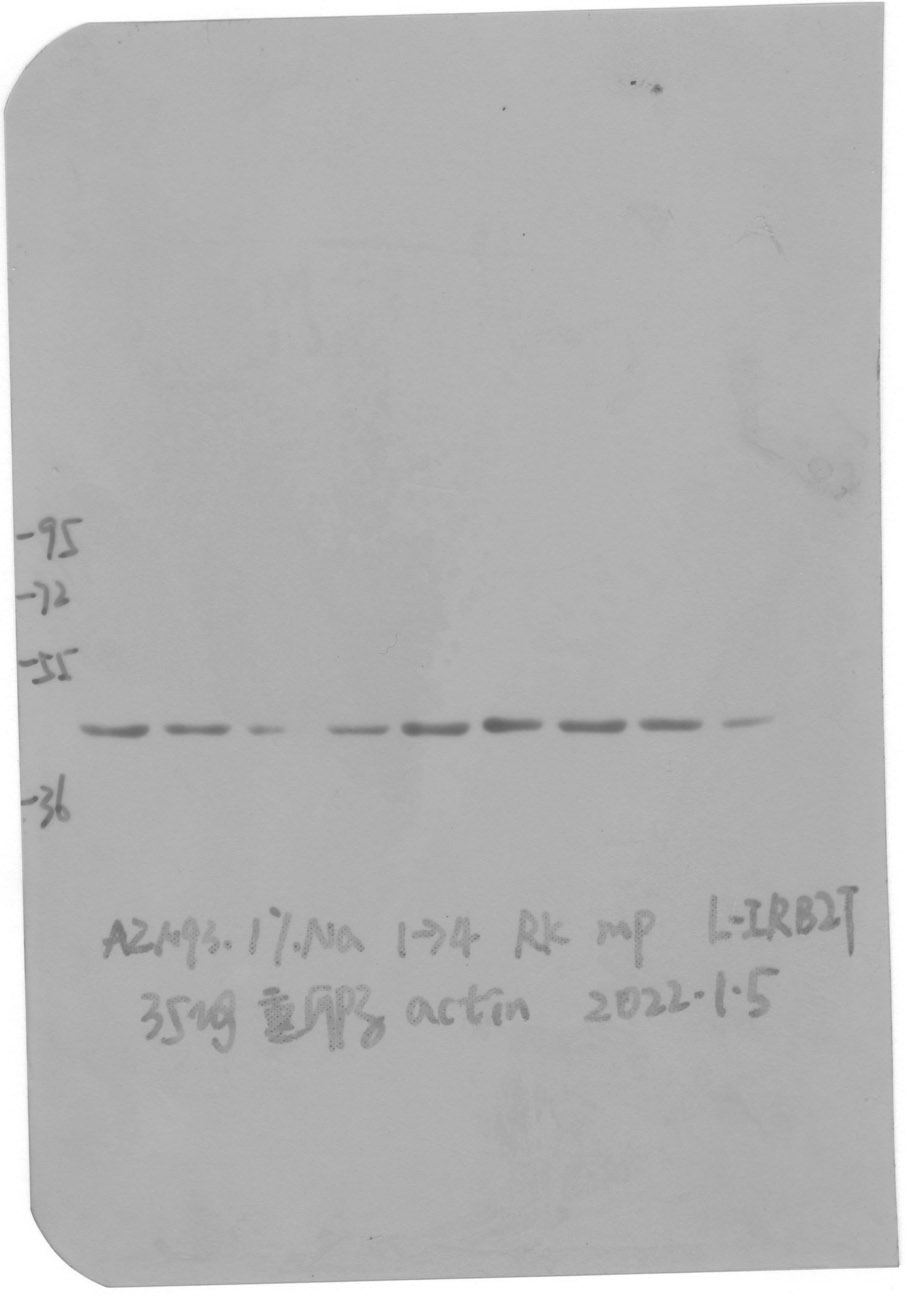

Supplement: Supplementary file 3 [file DataSheet2.ZIP › Small sized/Figure 10C-1% Na-Actin.jpg]

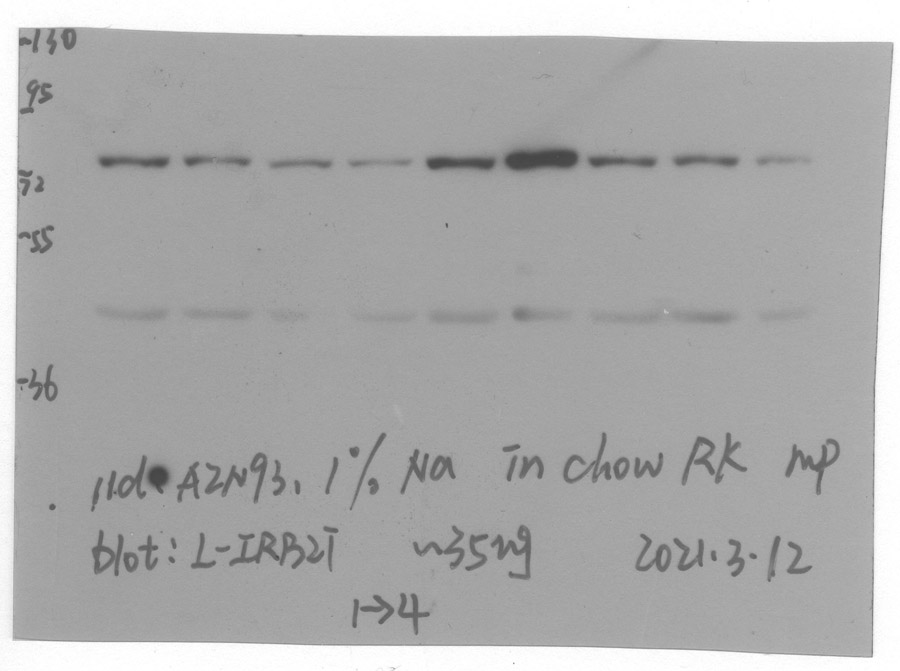

Supplement: Supplementary file 3 [file DataSheet2.ZIP › Small sized/Figure 10C-1% Na-L-IRBIT.jpg]

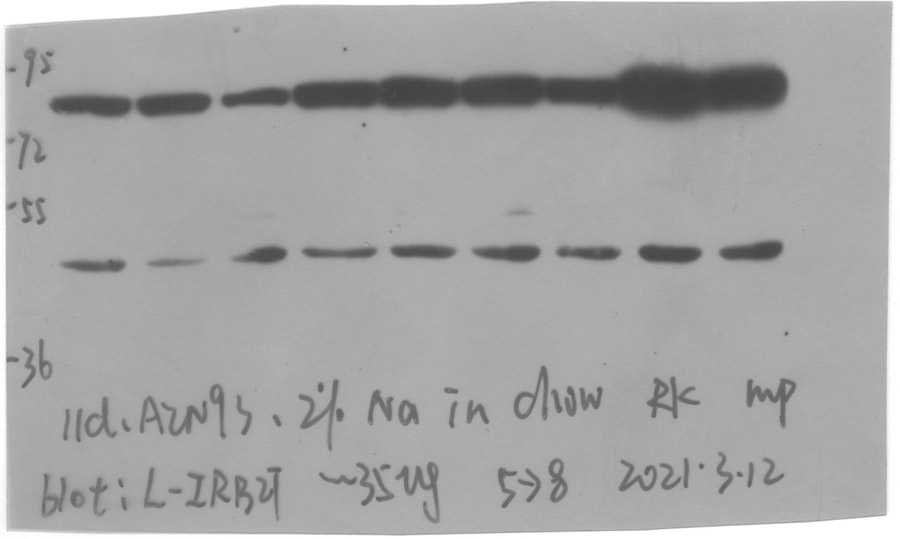

Supplement: Supplementary file 3 [file DataSheet2.ZIP › Small sized/Figure 10C-2% Na-Actin.jpg]

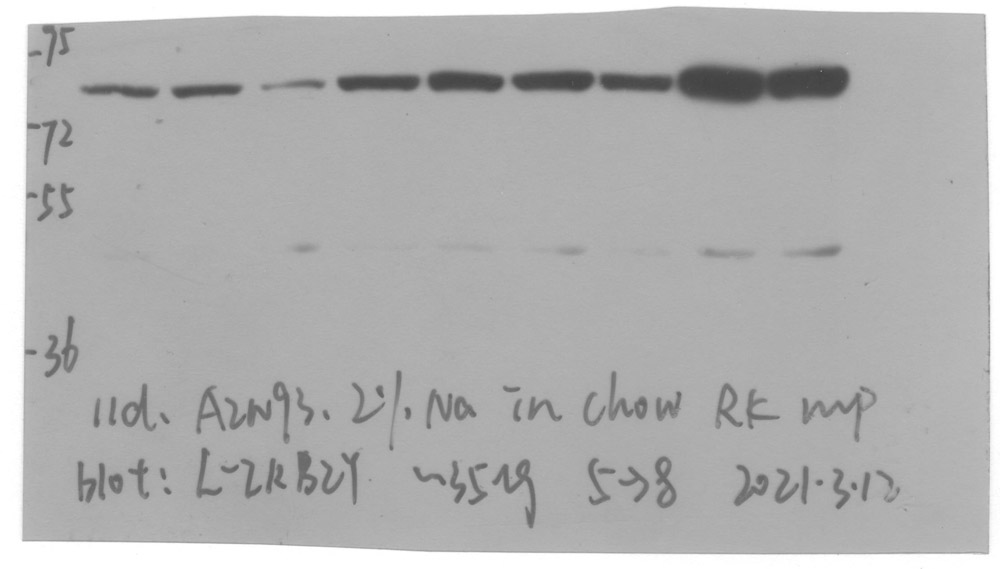

Supplement: Supplementary file 3 [file DataSheet2.ZIP › Small sized/Figure 10C-2% Na-L-IRBIT.jpg]

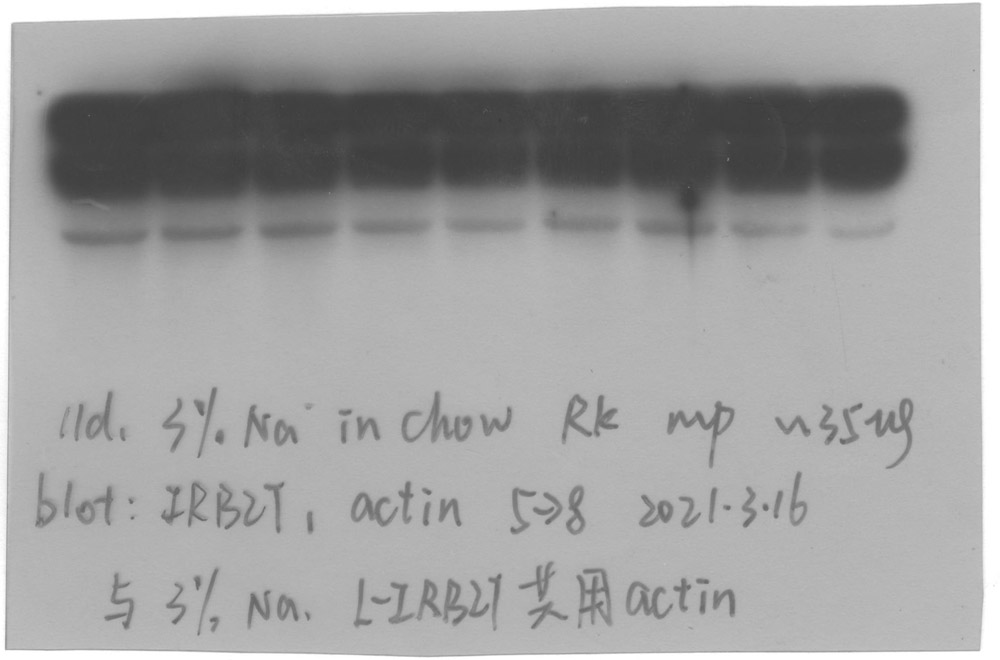

Supplement: Supplementary file 3 [file DataSheet2.ZIP › Small sized/Figure 10C-3% Na-Actin.jpg]

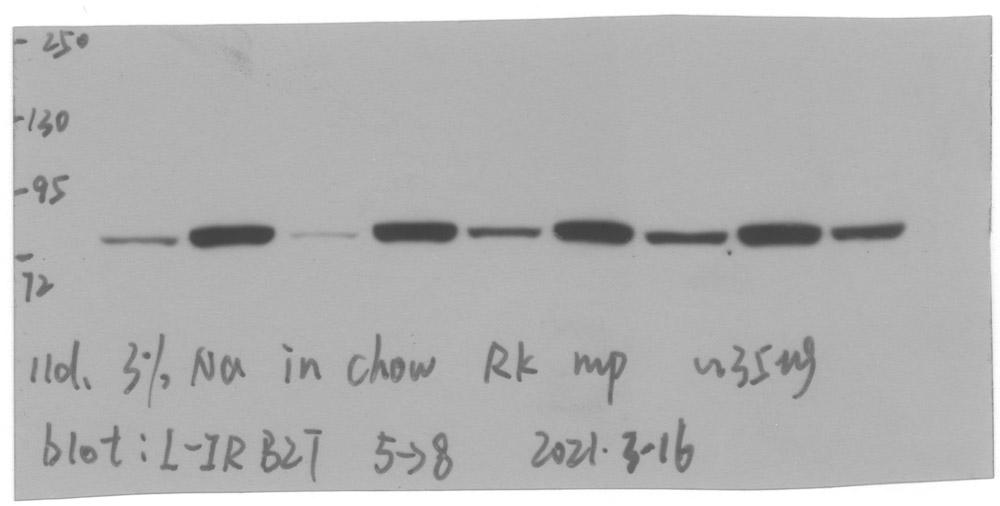

Supplement: Supplementary file 3 [file DataSheet2.ZIP › Small sized/Figure 10C-3% Na-L-IRBIT.jpg]

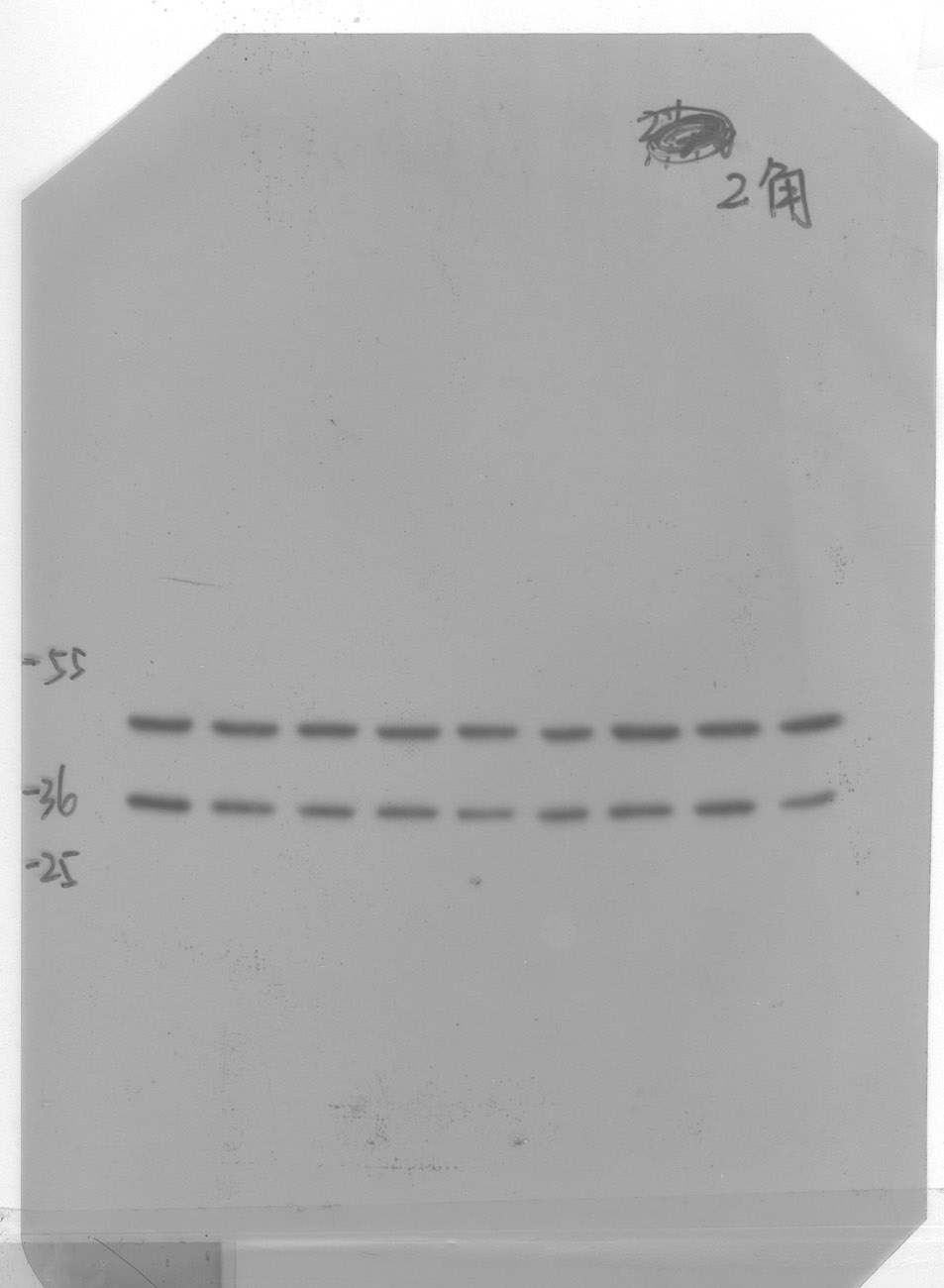

Supplement: Supplementary file 3 [file DataSheet2.ZIP › Small sized/Figure 10E-1% Na-PP1+Actin.jpg]

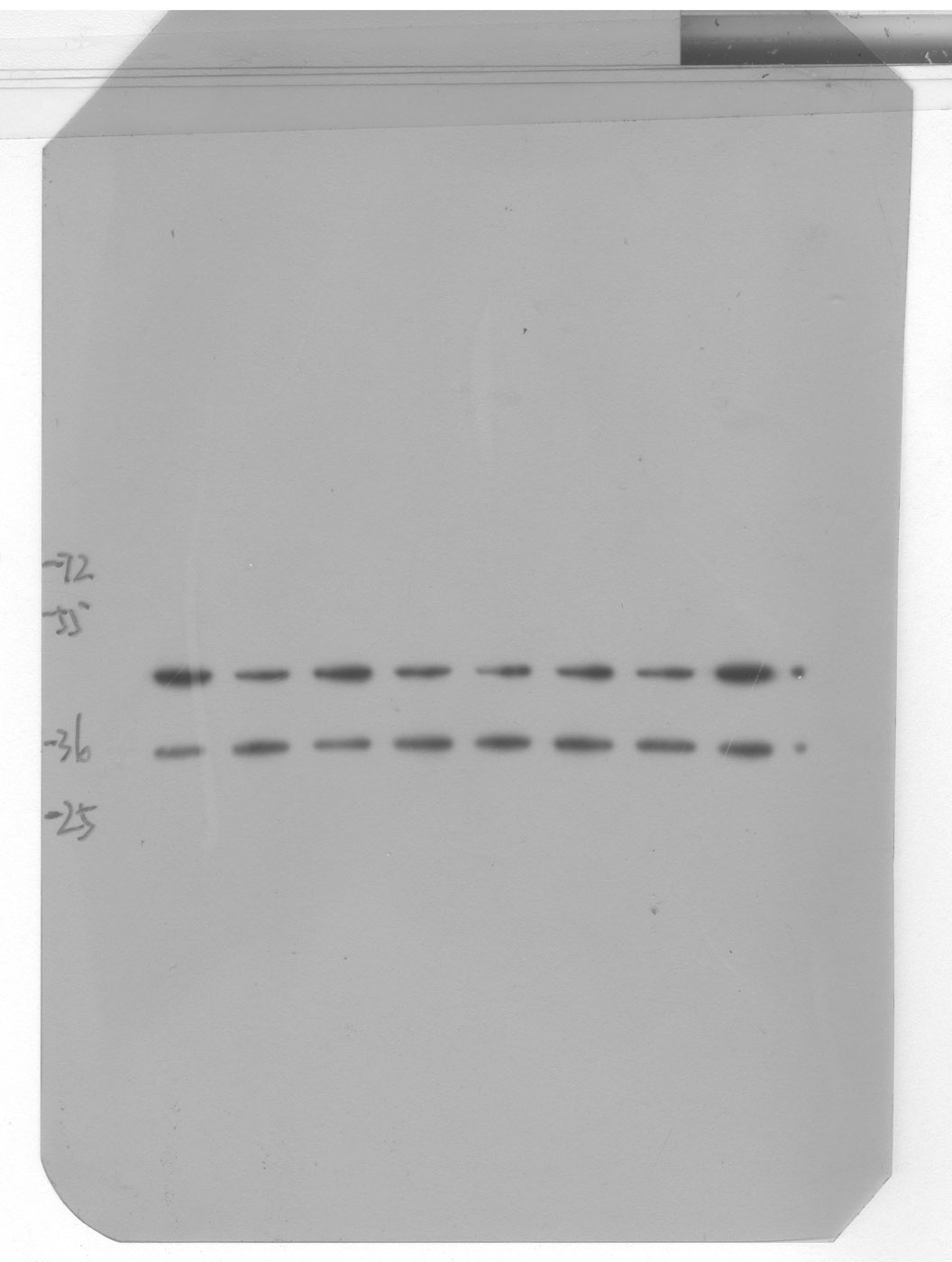

Supplement: Supplementary file 3 [file DataSheet2.ZIP › Small sized/Figure 10E-2% Na-PP1+Actin.jpg]

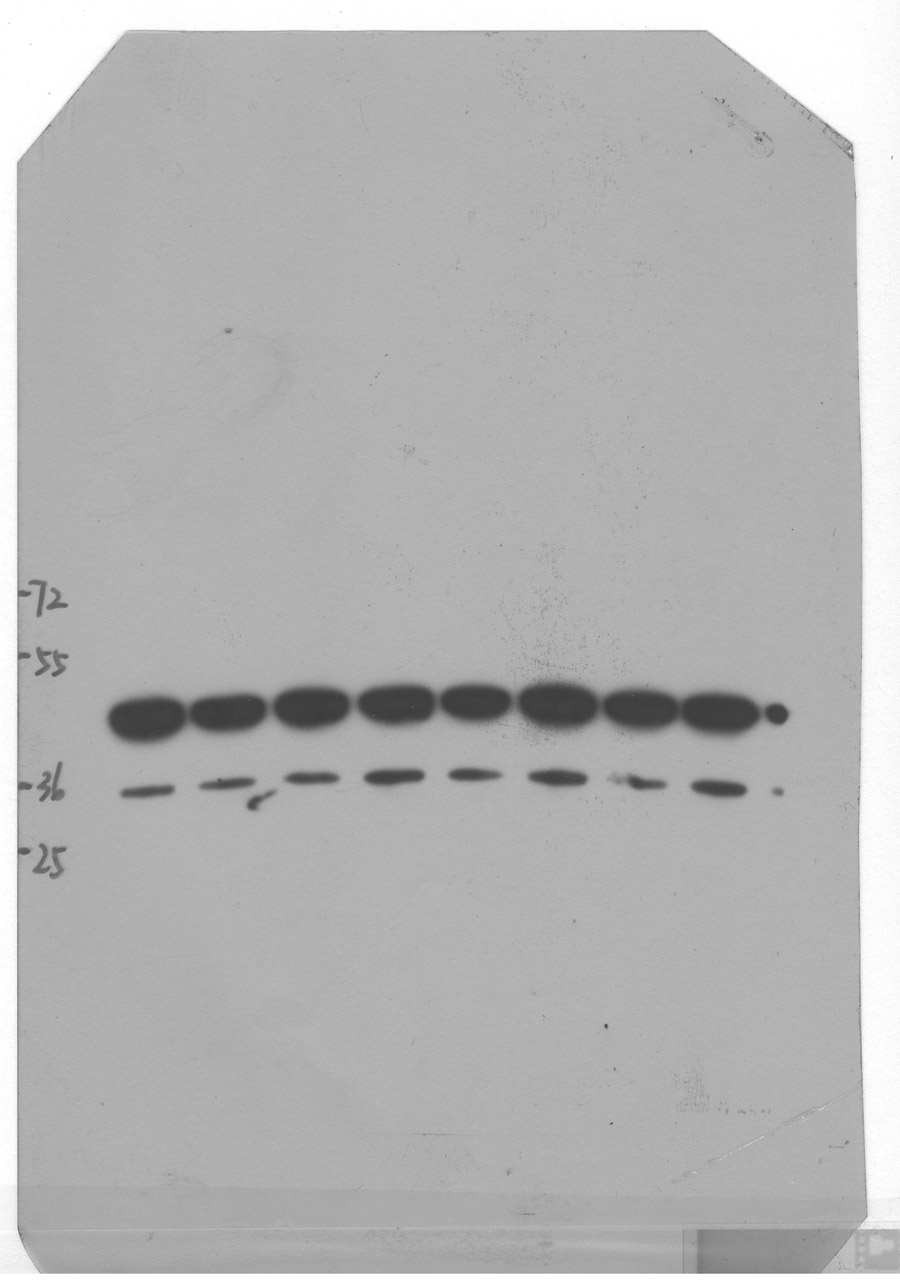

Supplement: Supplementary file 3 [file DataSheet2.ZIP › Small sized/Figure 10E-3 Na-PP1.jpg]

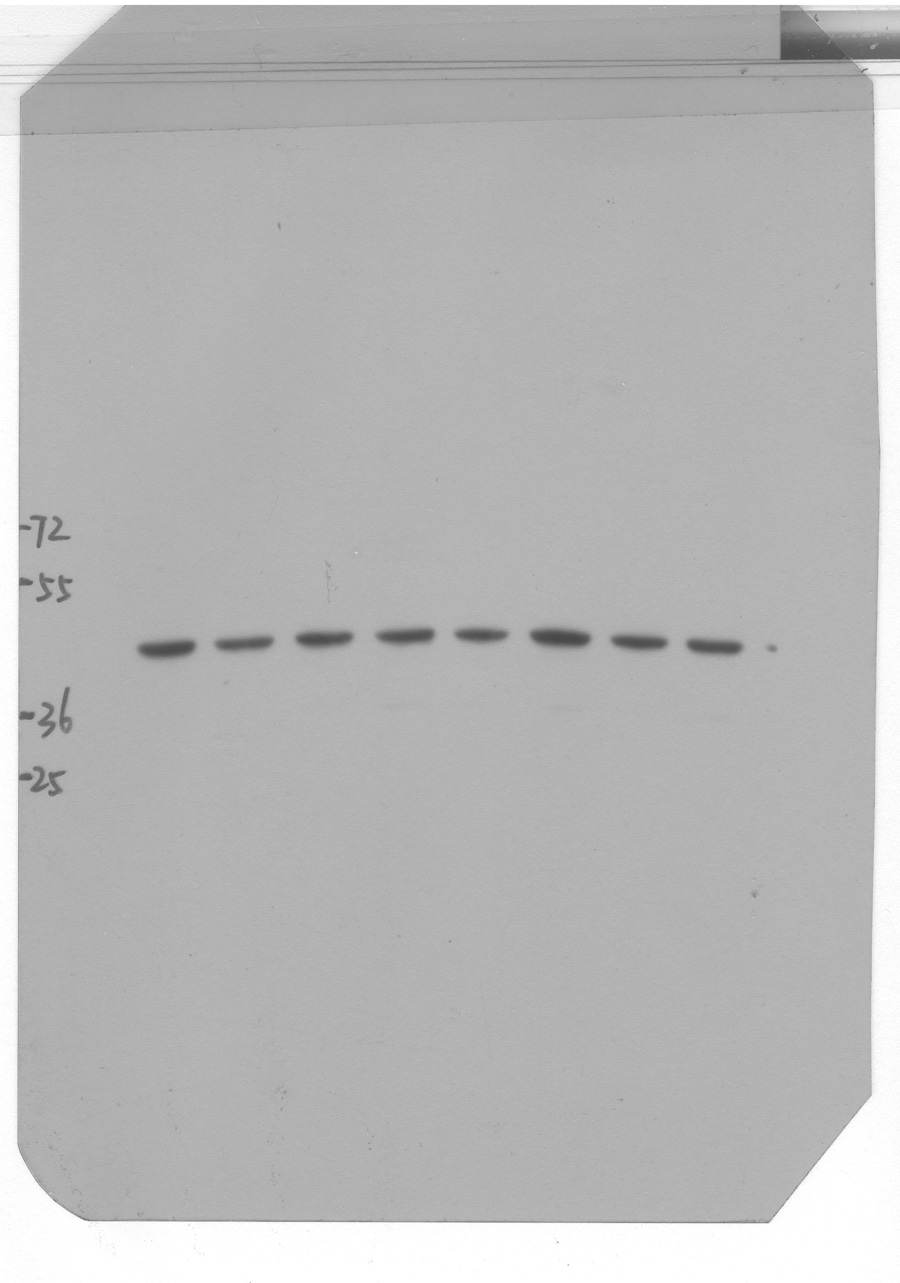

Supplement: Supplementary file 3 [file DataSheet2.ZIP › Small sized/Figure 10E-3% Na-Actin.jpg]

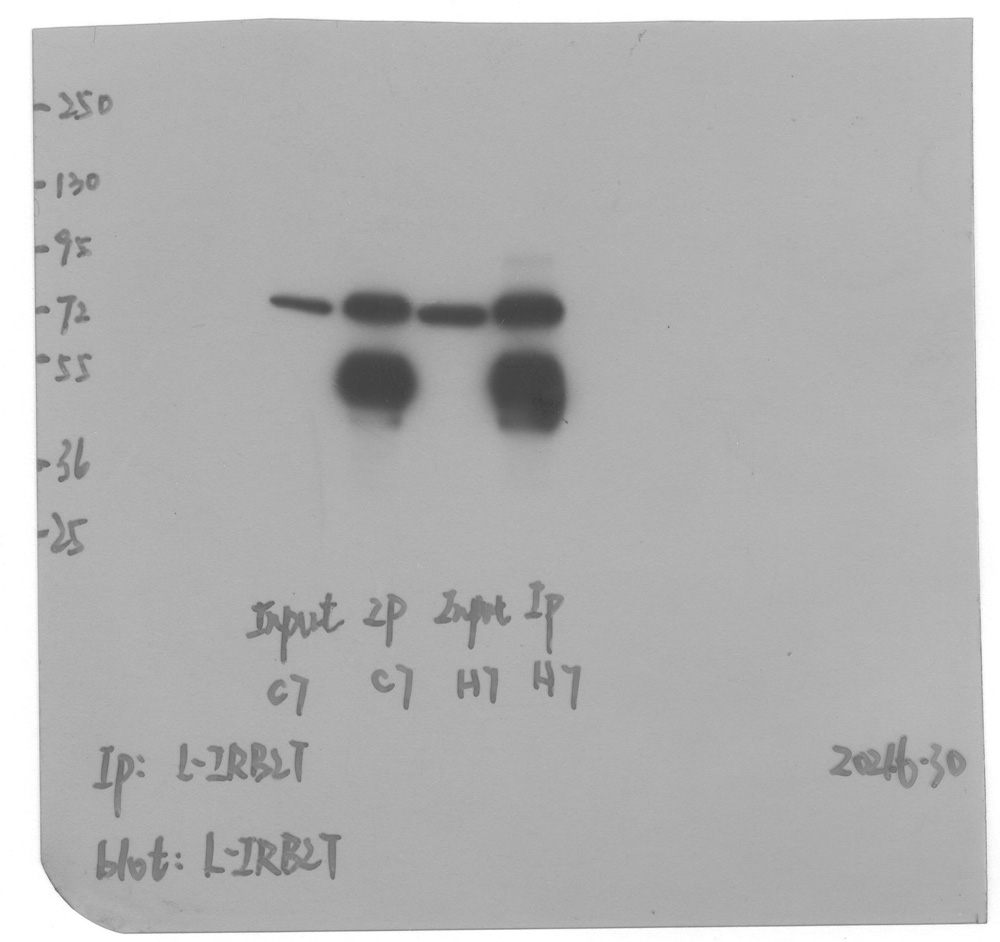

Supplement: Supplementary file 3 [file DataSheet2.ZIP › Small sized/Figure 11E-L-IRBIT.jpg]

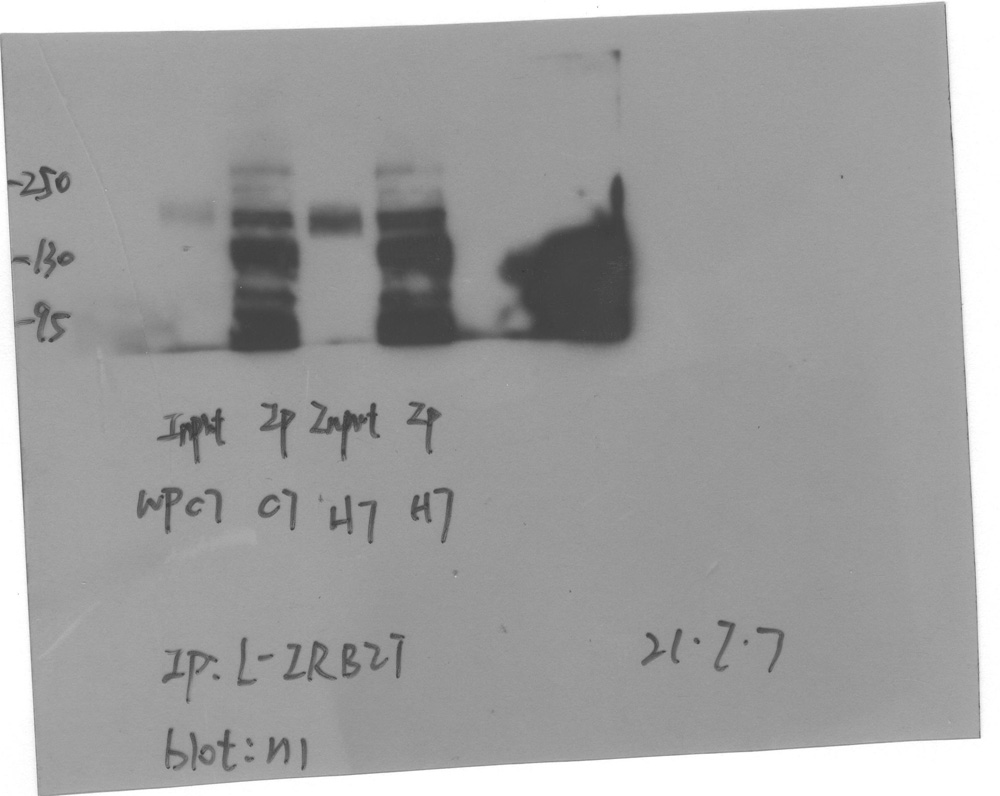

Supplement: Supplementary file 3 [file DataSheet2.ZIP › Small sized/Figure 11E-NBCn1.jpg]

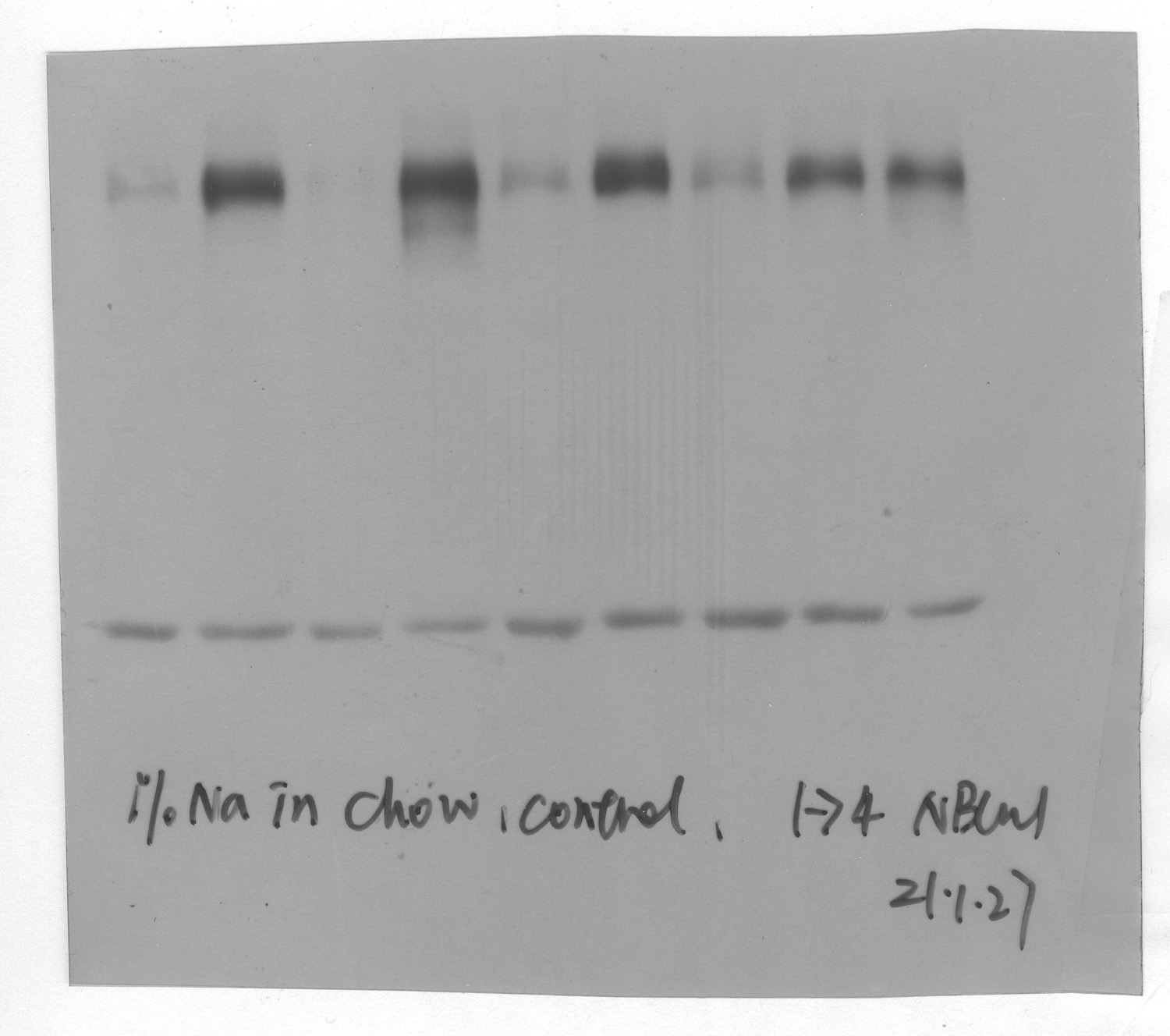

Supplement: Supplementary file 3 [file DataSheet2.ZIP › Small sized/Figure 9A-1% Na-NBCn1+Actin.jpg]

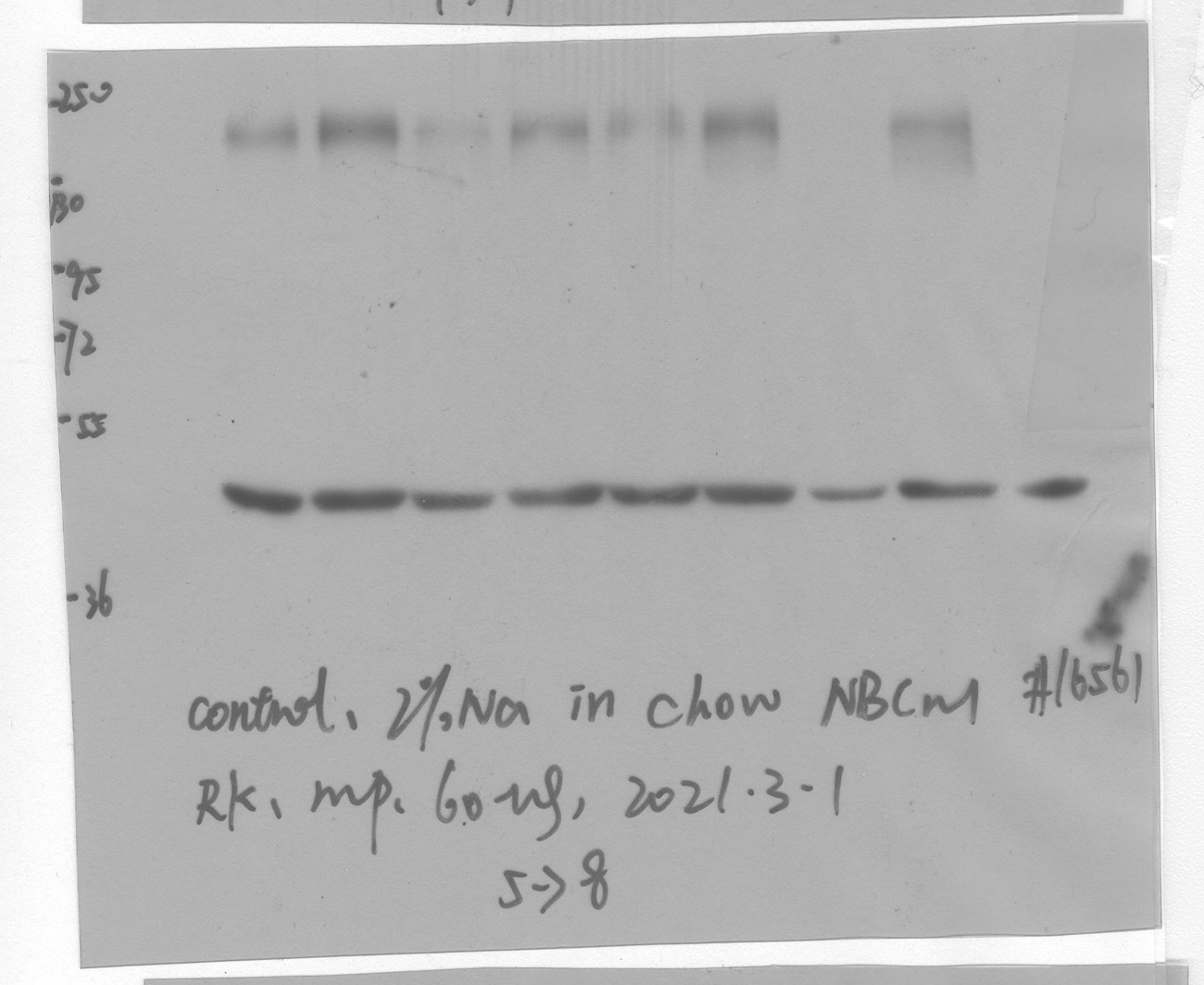

Supplement: Supplementary file 3 [file DataSheet2.ZIP › Small sized/Figure 9A-2% Na-Actin.jpg]

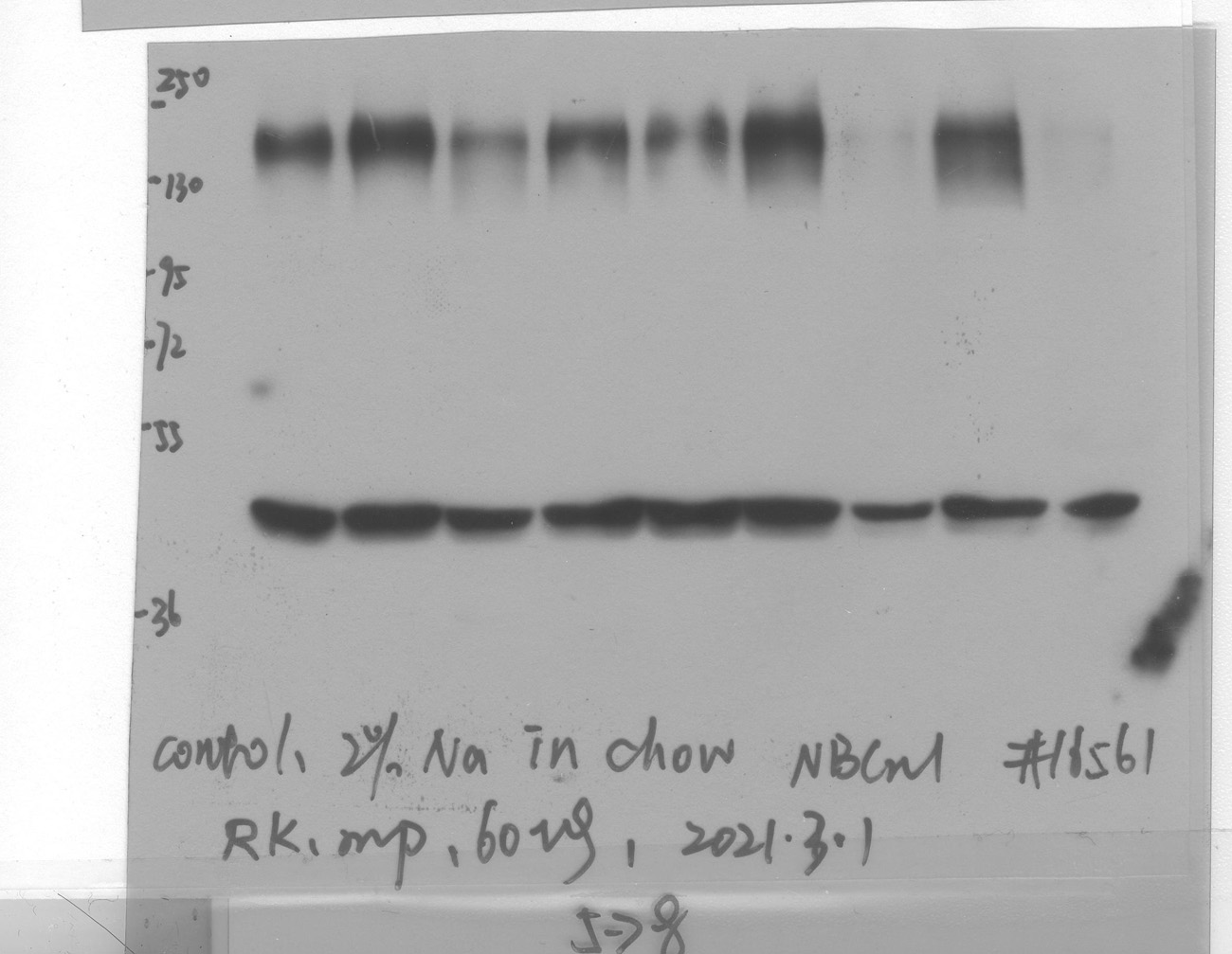

Supplement: Supplementary file 3 [file DataSheet2.ZIP › Small sized/Figure 9A-2% Na-NBCn1.jpg]

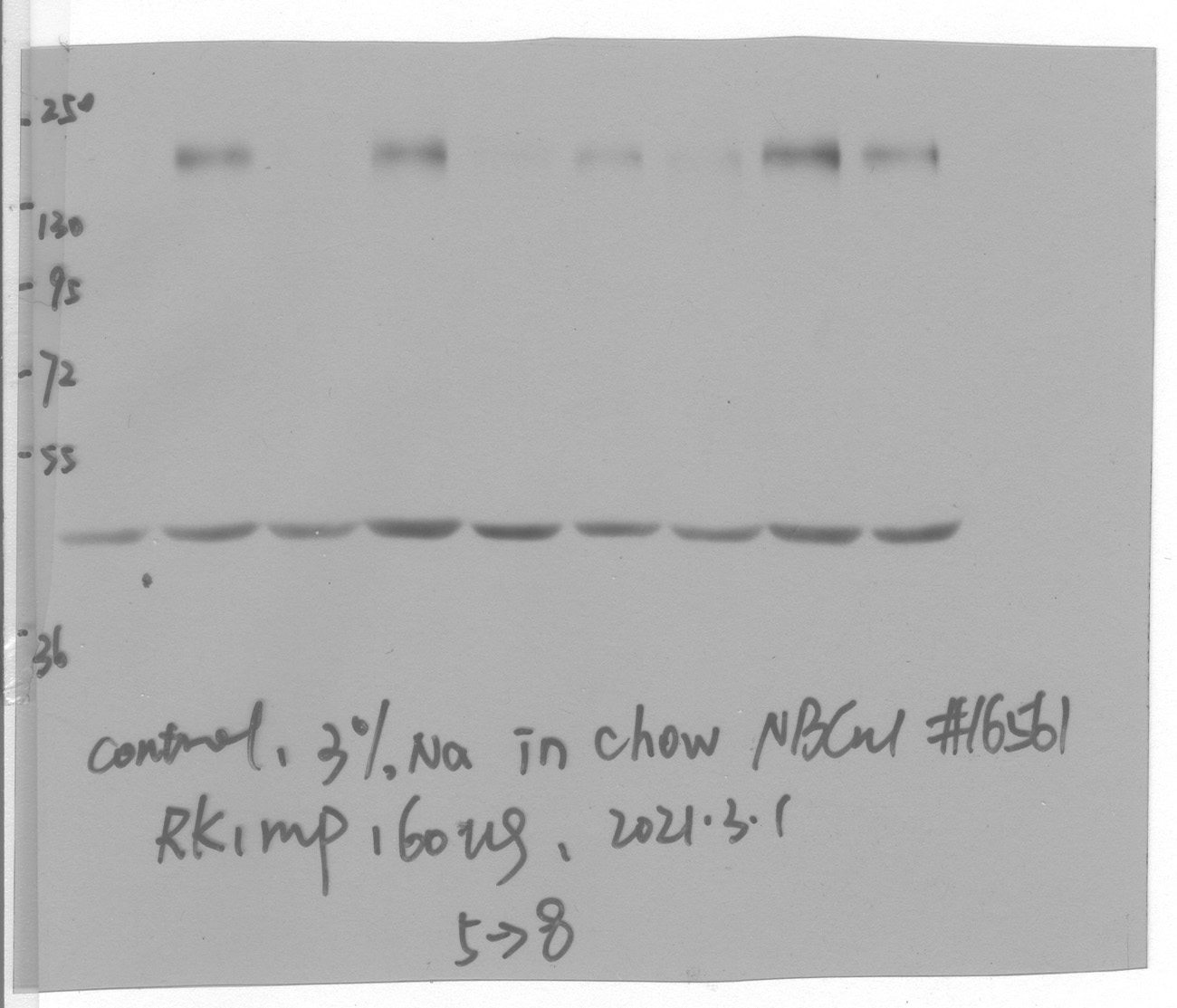

Supplement: Supplementary file 3 [file DataSheet2.ZIP › Small sized/Figure 9A-3% Na-Actin.jpg]

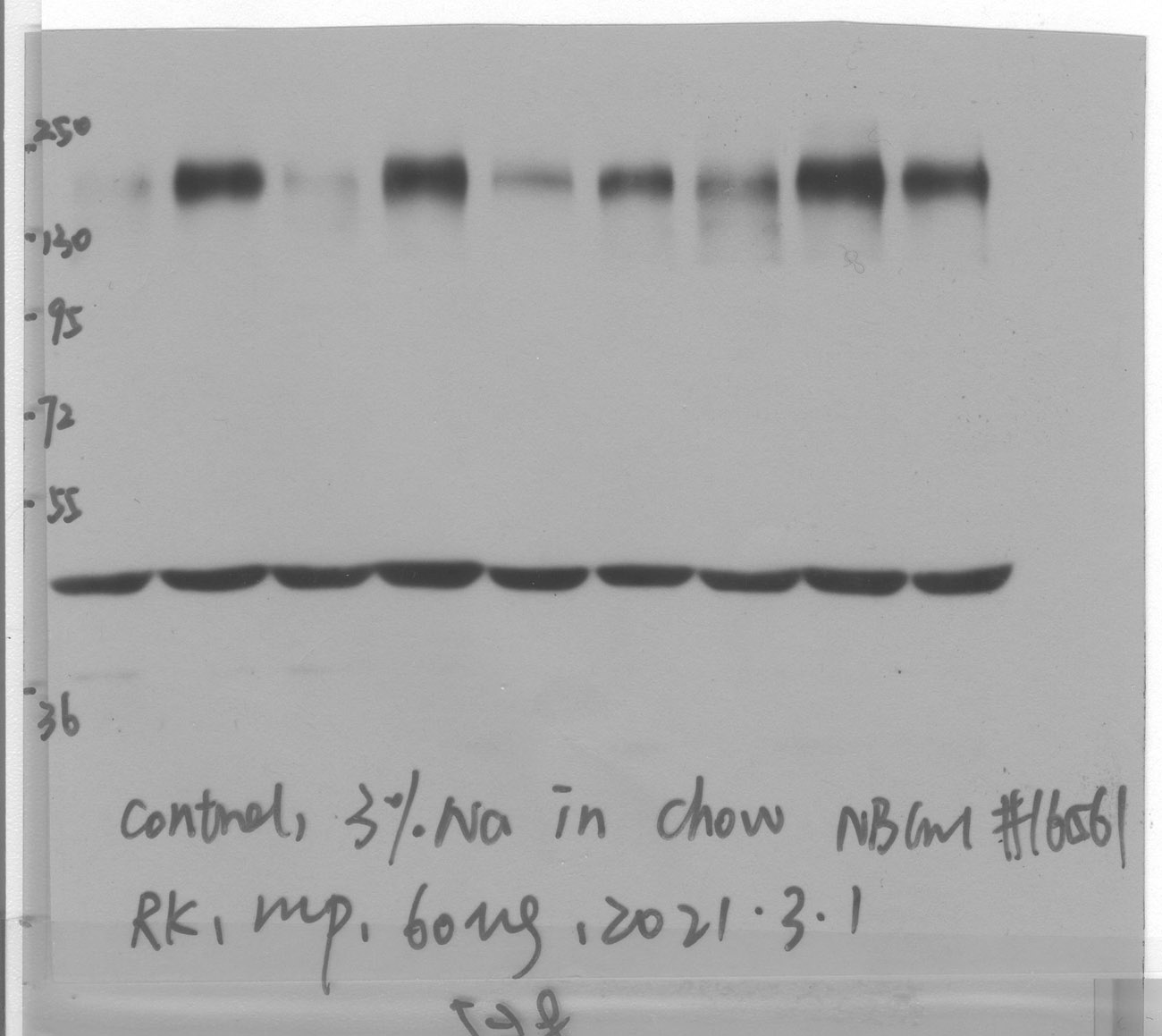

Supplement: Supplementary file 3 [file DataSheet2.ZIP › Small sized/Figure 9A-3% Na-NBCn1.jpg]

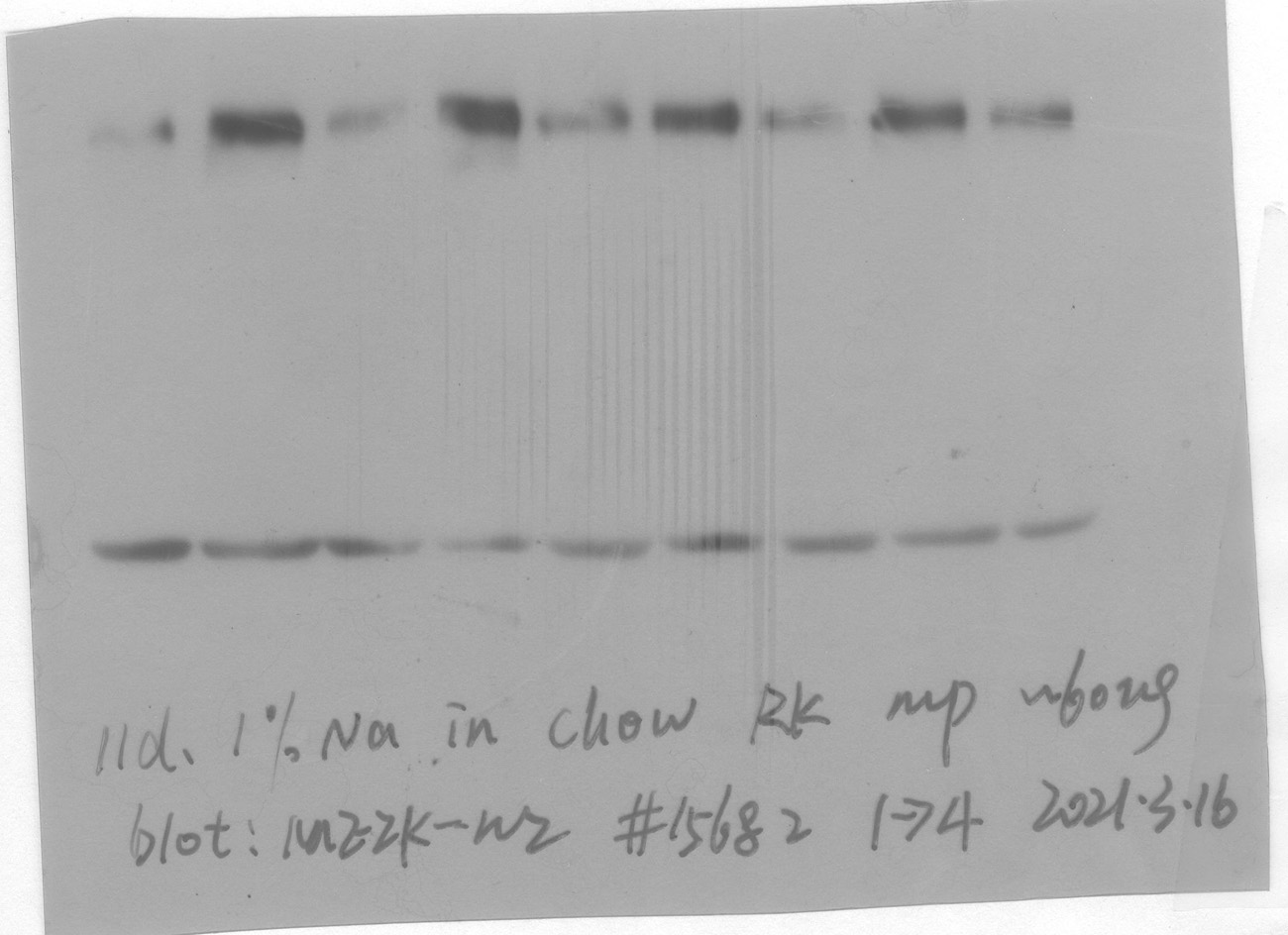

Supplement: Supplementary file 3 [file DataSheet2.ZIP › Small sized/Figure 9C-1% Na-NBCn2+Actin.jpg]

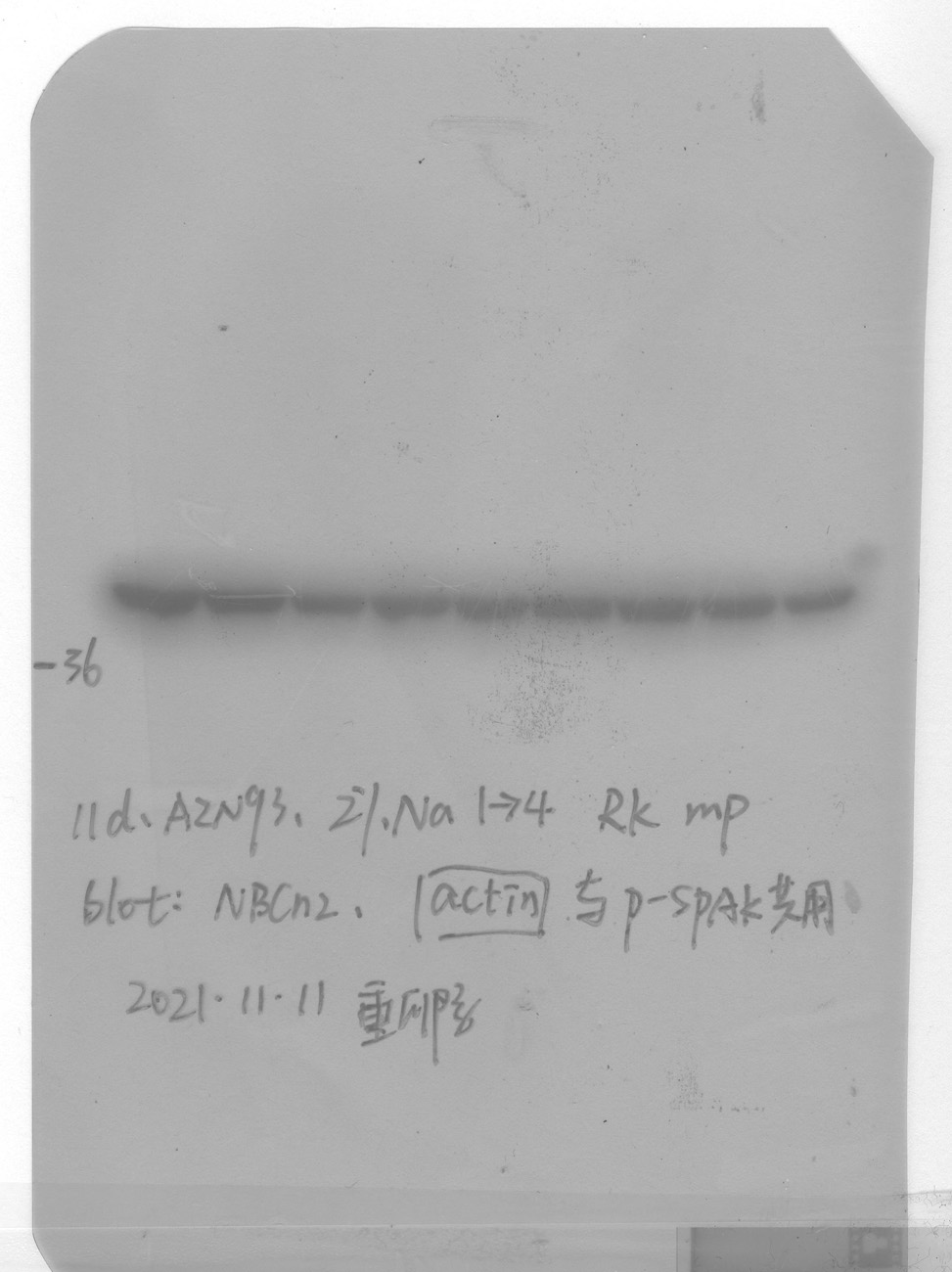

Supplement: Supplementary file 3 [file DataSheet2.ZIP › Small sized/Figure 9C-2% Na-Actin.jpg]

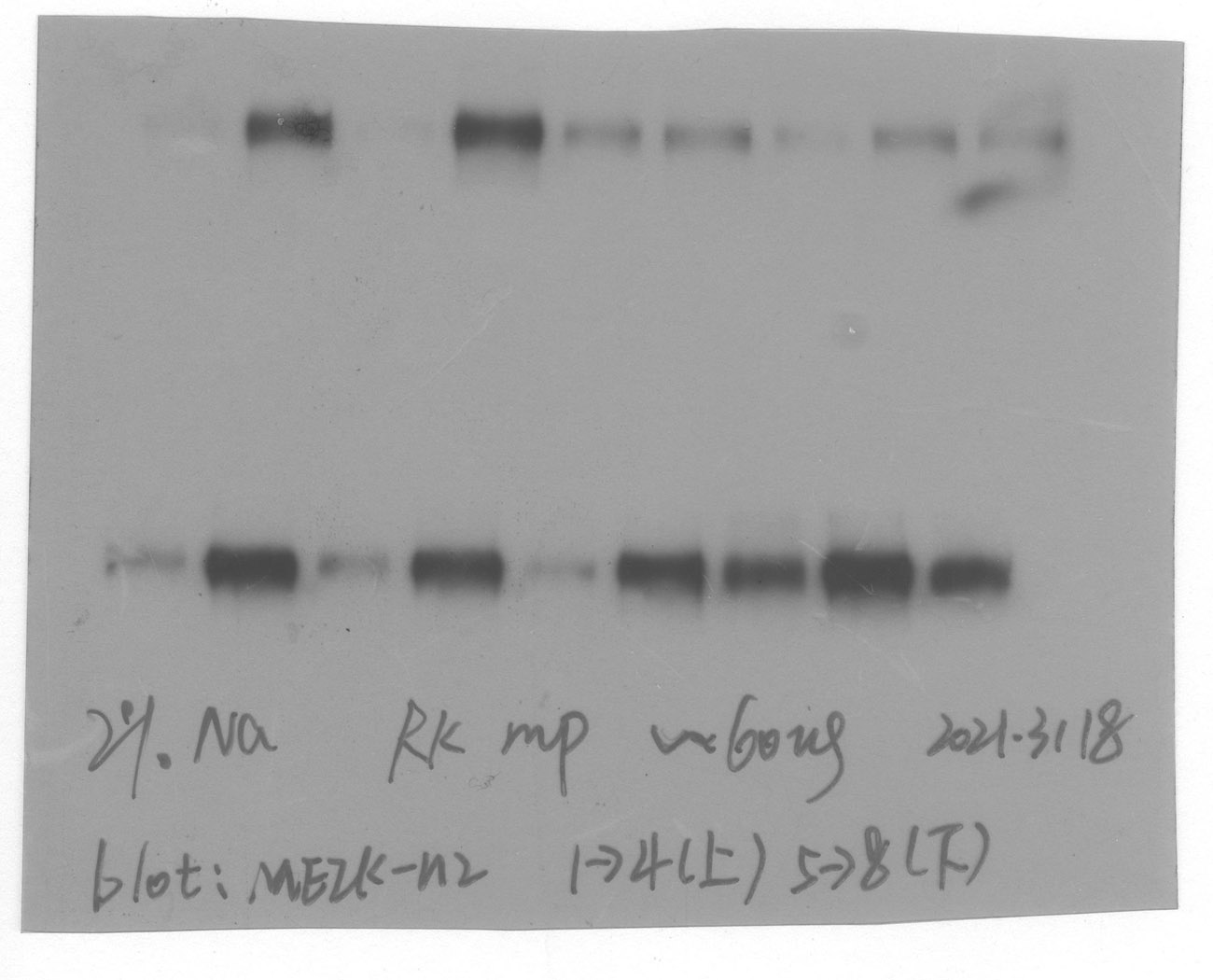

Supplement: Supplementary file 3 [file DataSheet2.ZIP › Small sized/Figure 9C-2% Na-NBCn2.jpg]

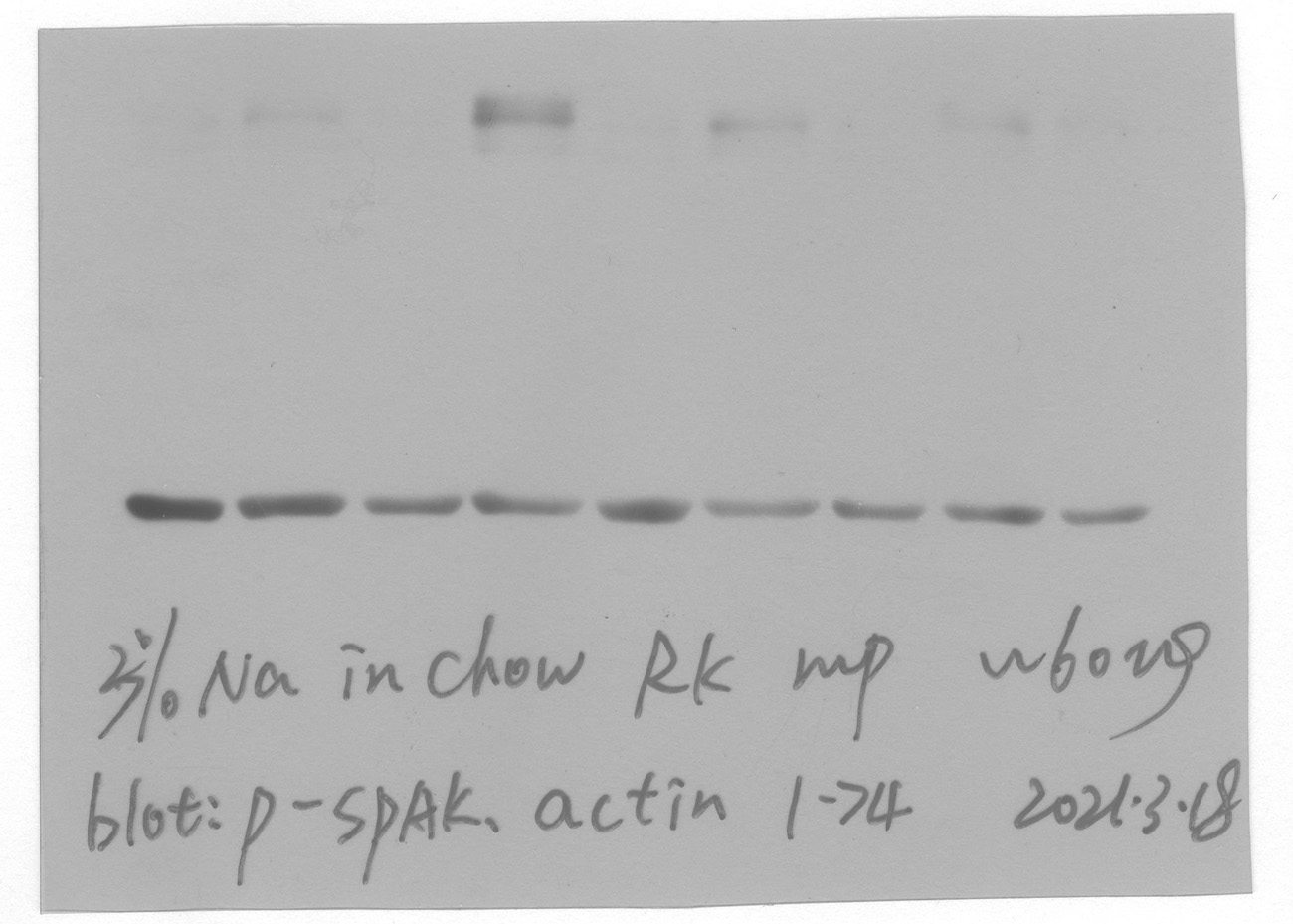

Supplement: Supplementary file 3 [file DataSheet2.ZIP › Small sized/Figure 9C-3% Na-NBCn2-actin.jpg]

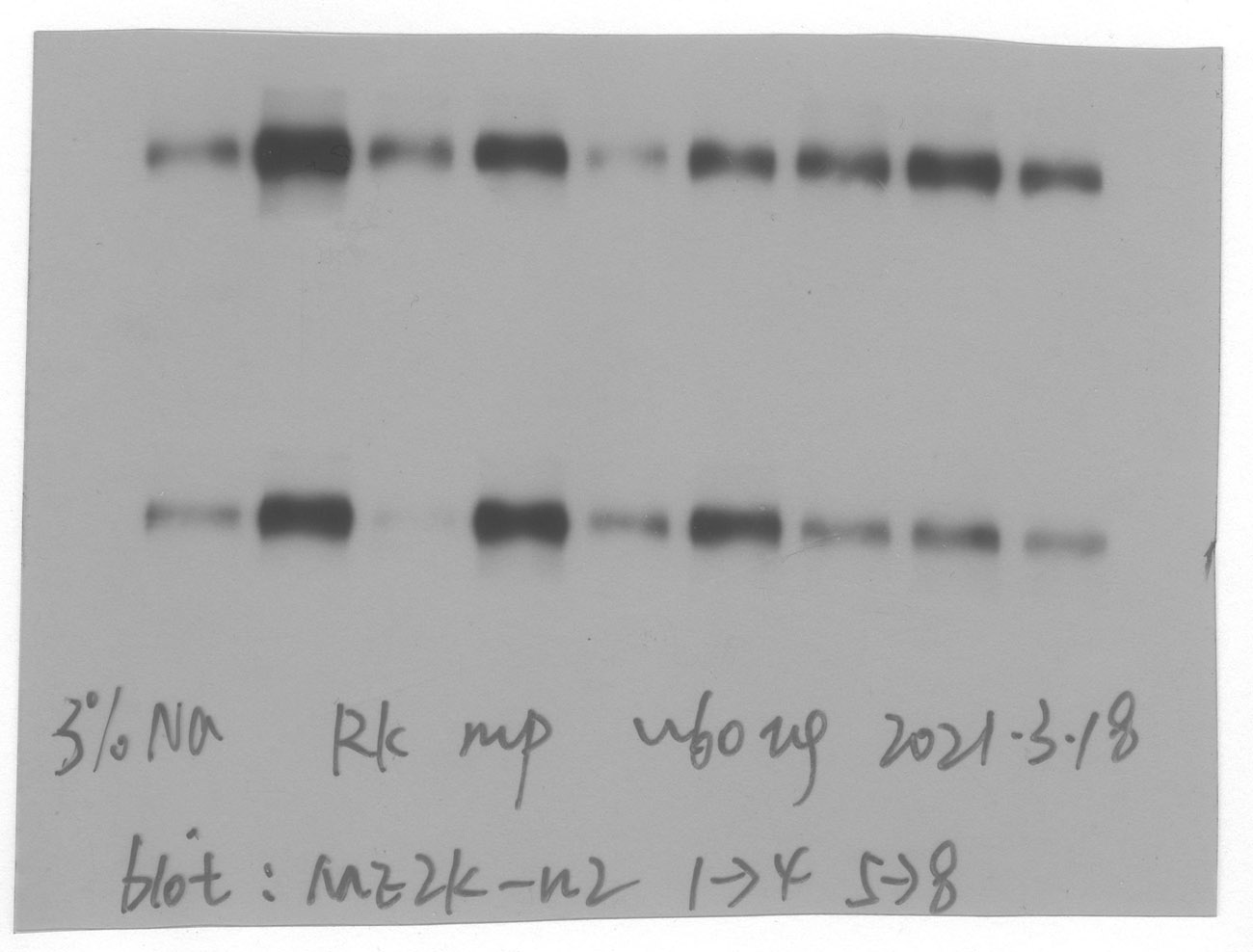

Supplement: Supplementary file 3 [file DataSheet2.ZIP › Small sized/Figure 9C-3% Na-NBCn2.jpg]

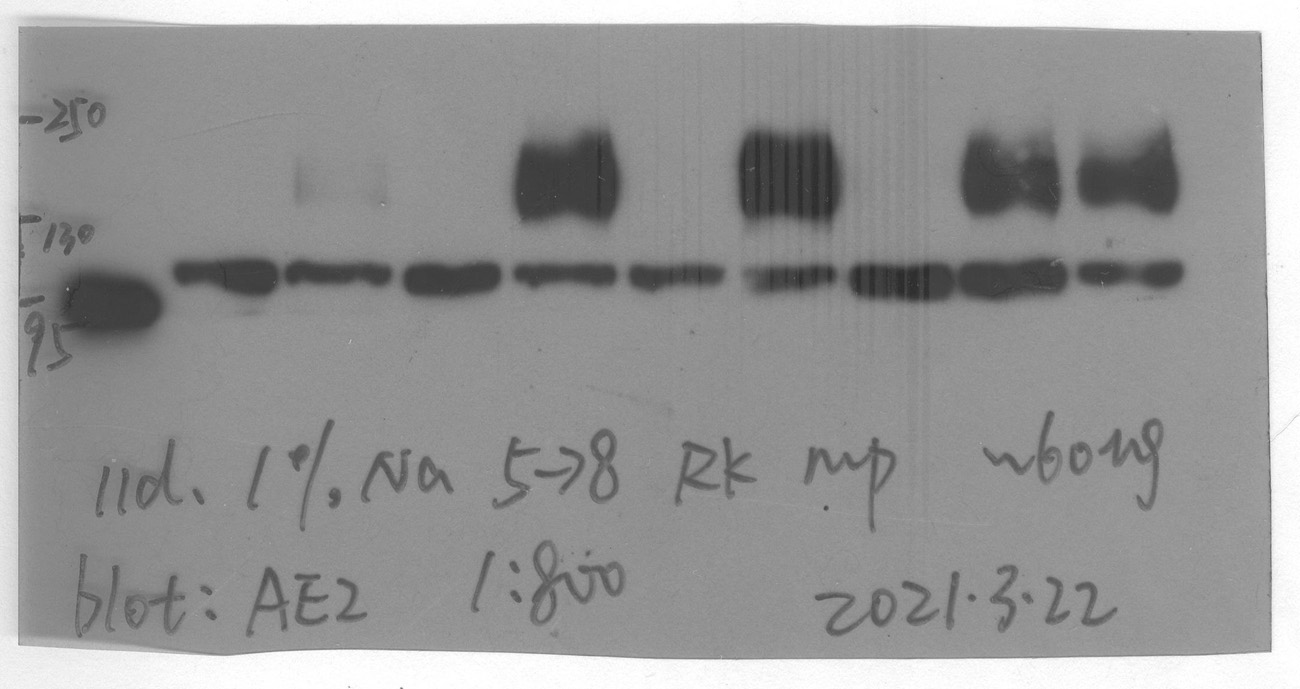

Supplement: Supplementary file 3 [file DataSheet2.ZIP › Small sized/Figure 9E-1% Na-AE2.jpg]

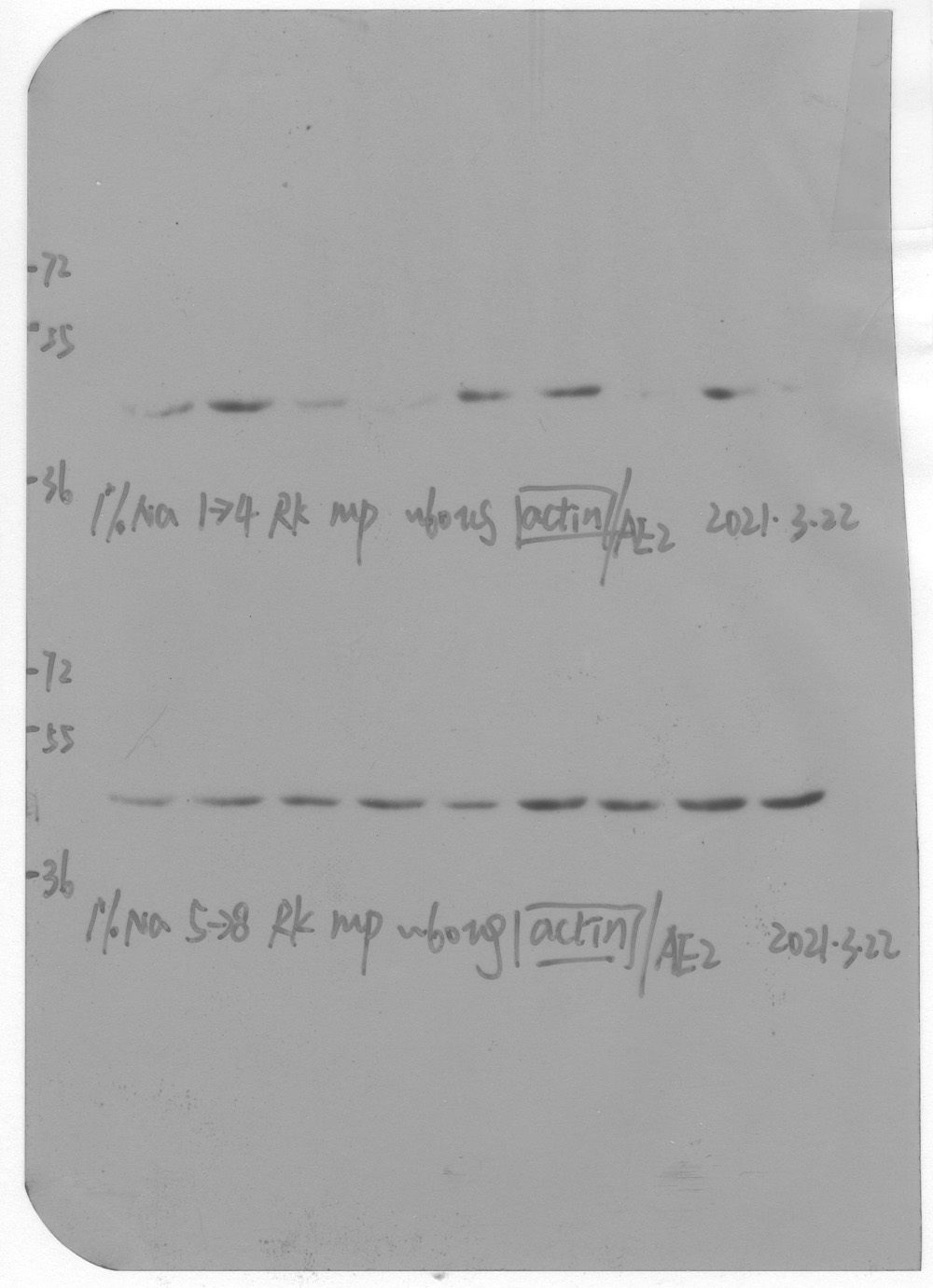

Supplement: Supplementary file 3 [file DataSheet2.ZIP › Small sized/Figure 9E-1% Na-Actin.jpg]

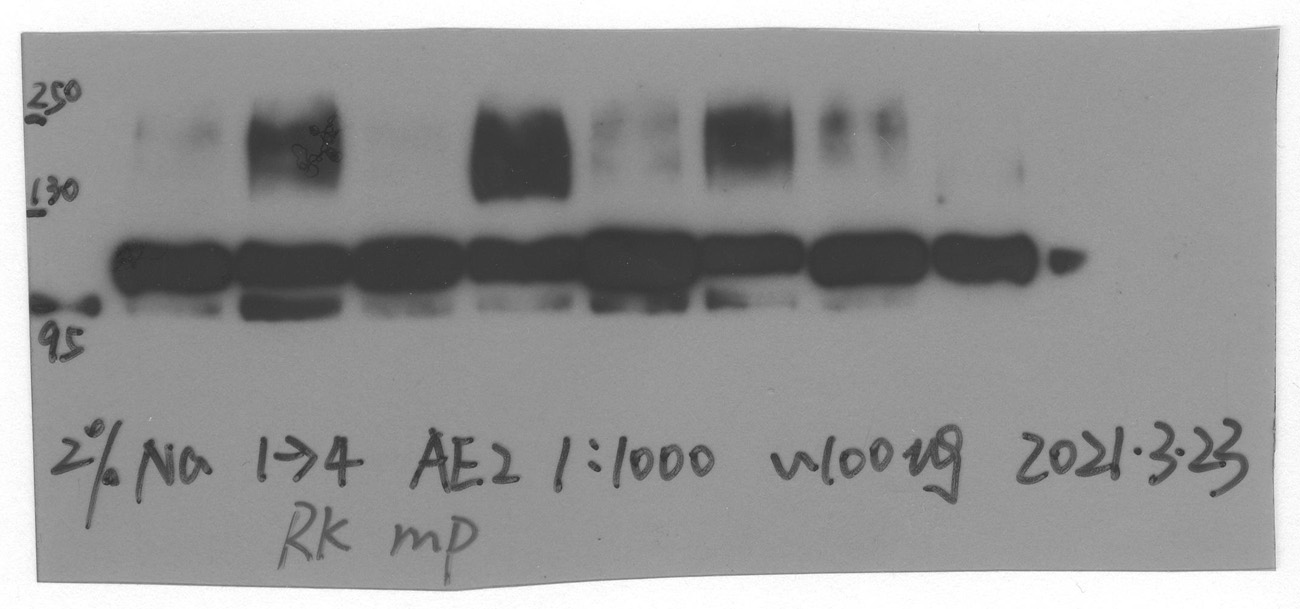

Supplement: Supplementary file 3 [file DataSheet2.ZIP › Small sized/Figure 9E-2% Na-AE2.jpg]

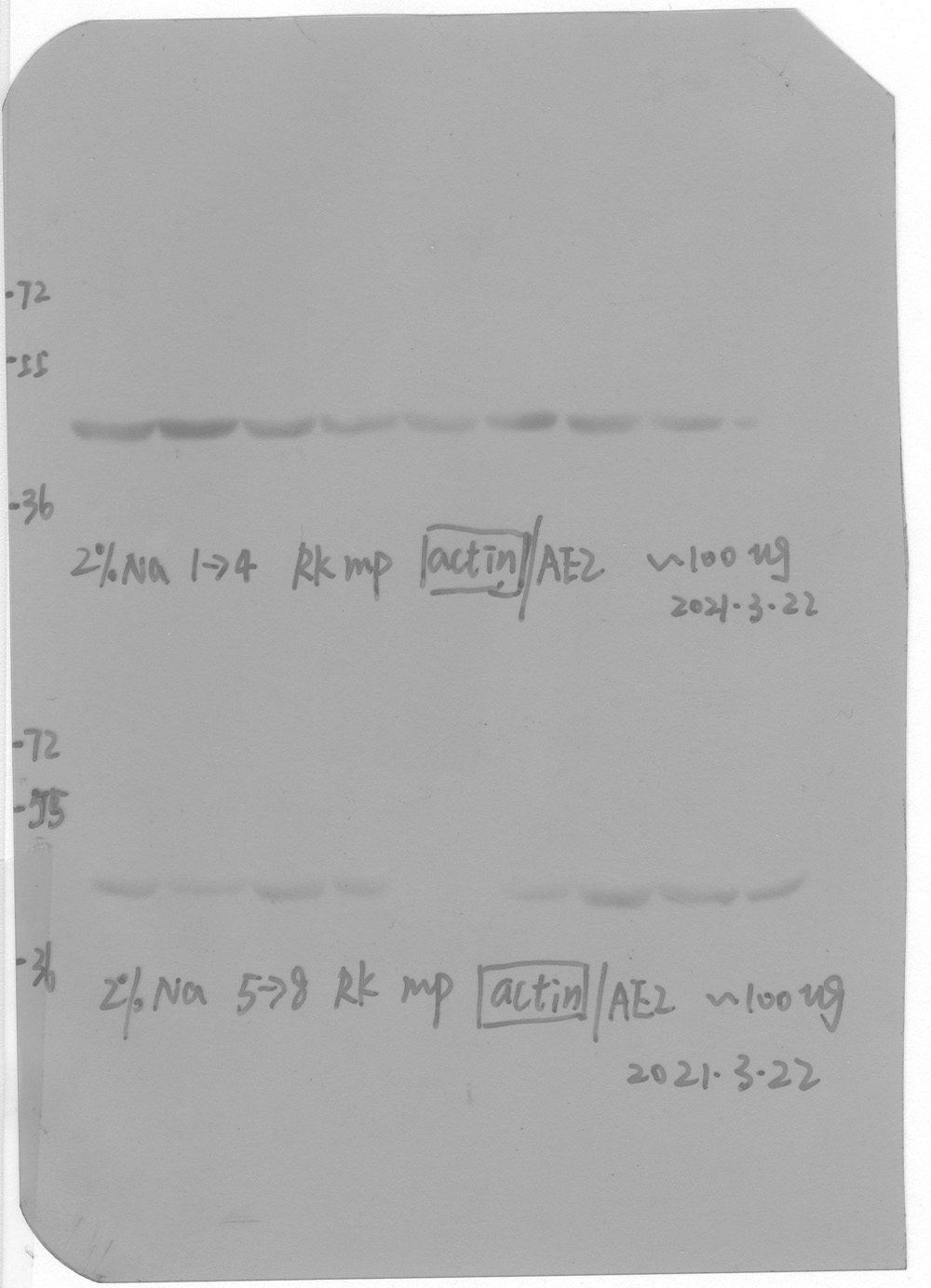

Supplement: Supplementary file 3 [file DataSheet2.ZIP › Small sized/Figure 9E-2% Na-Actin.jpg]

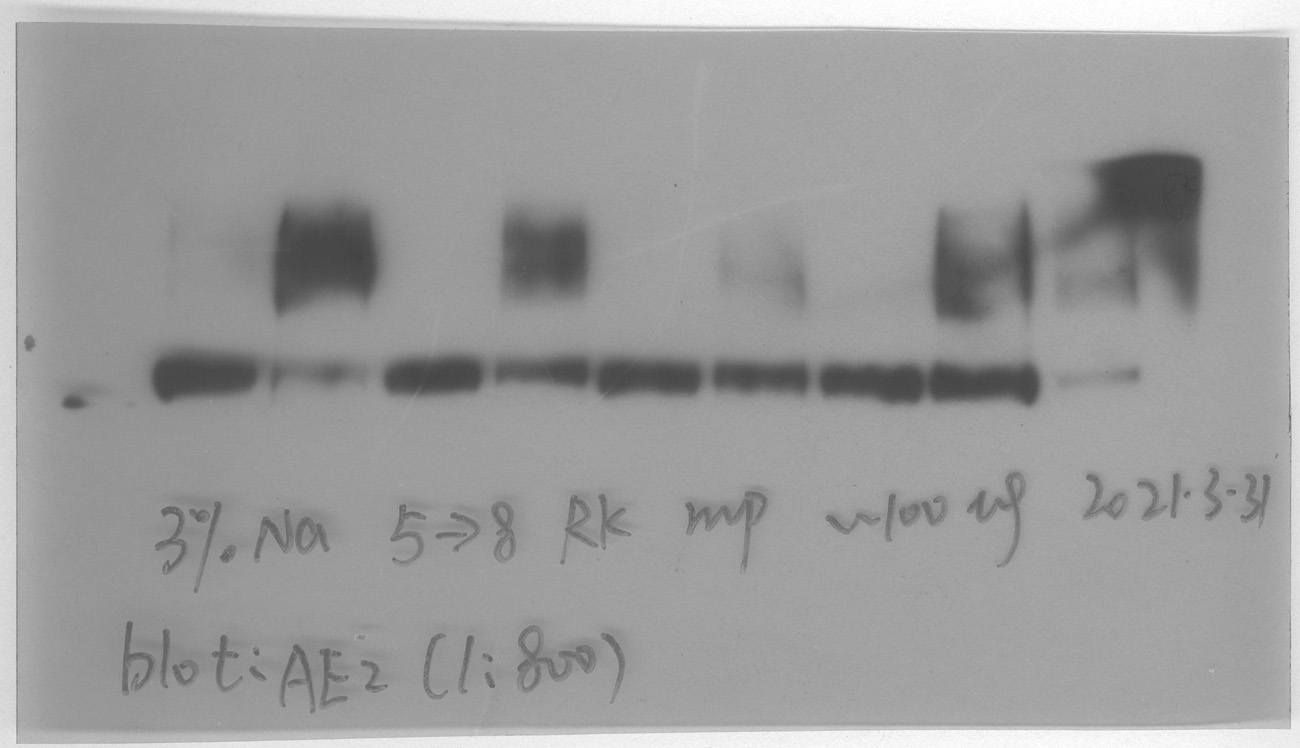

Supplement: Supplementary file 3 [file DataSheet2.ZIP › Small sized/Figure 9E-3% Na-AE2.jpg]

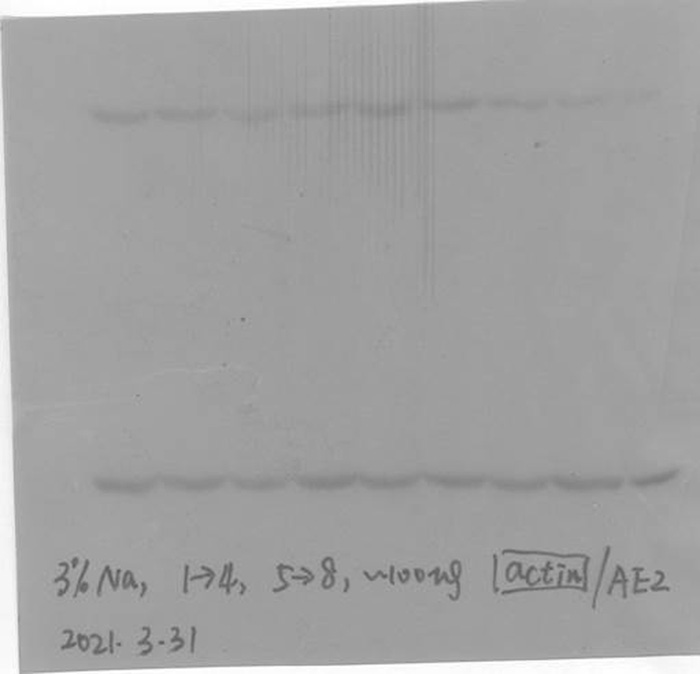

Supplement: Supplementary file 3 [file DataSheet2.ZIP › Small sized/Figure 9E-3% Na-Actin.jpg]

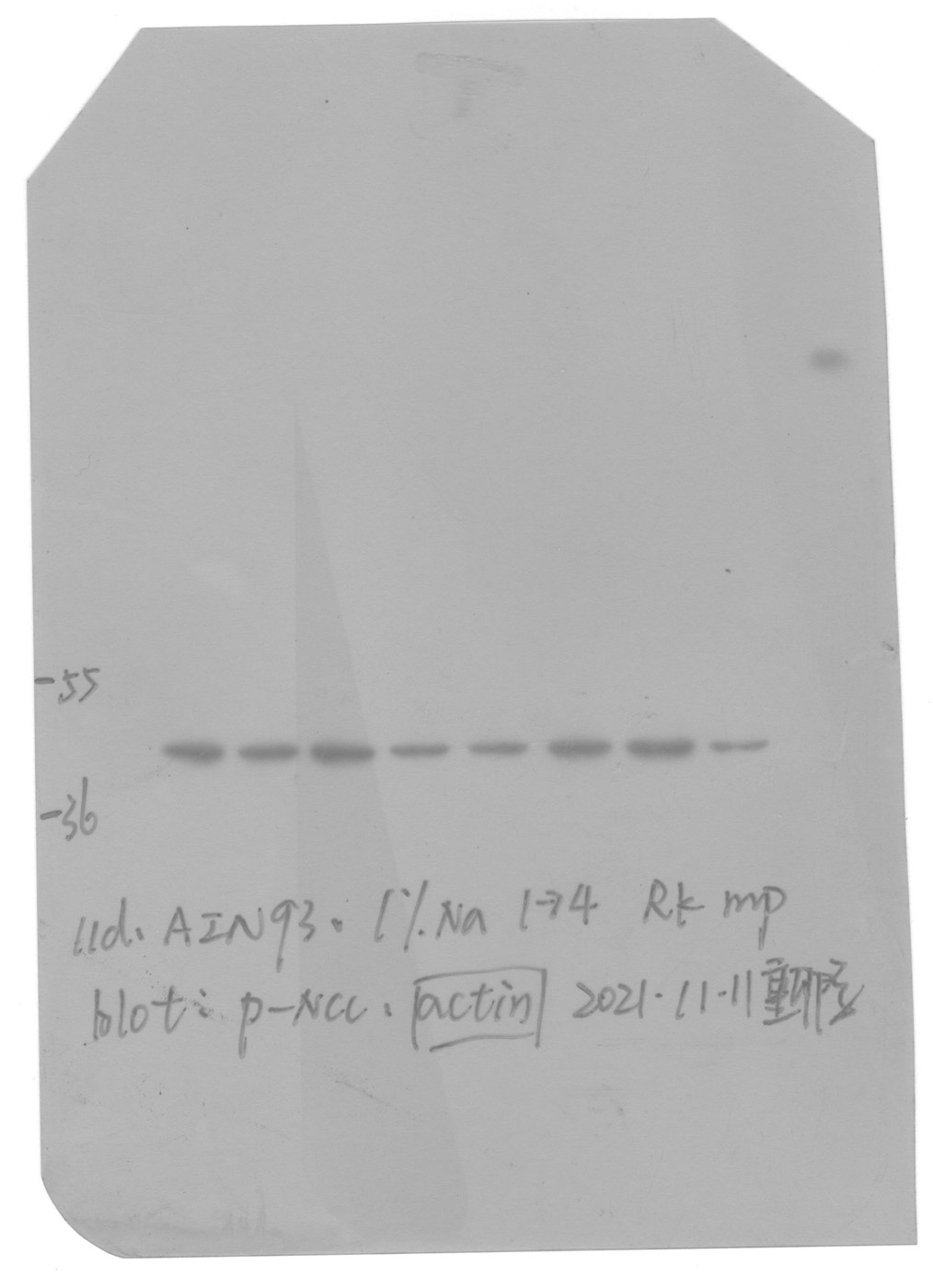

Supplement: Supplementary file 3 [file DataSheet2.ZIP › Small sized/Figure 9F-1% Na-Actin.jpg]

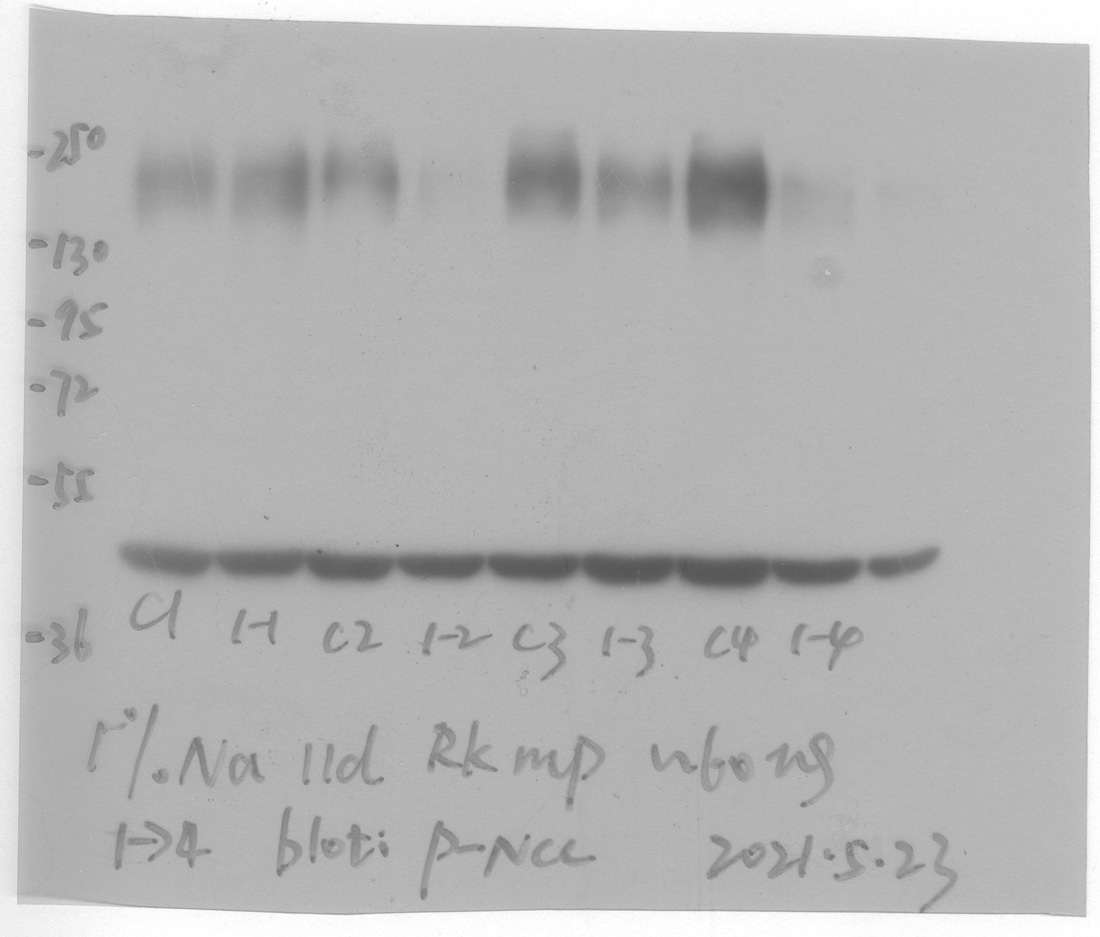

Supplement: Supplementary file 3 [file DataSheet2.ZIP › Small sized/Figure 9F-1% Na-p-NCC.jpg]

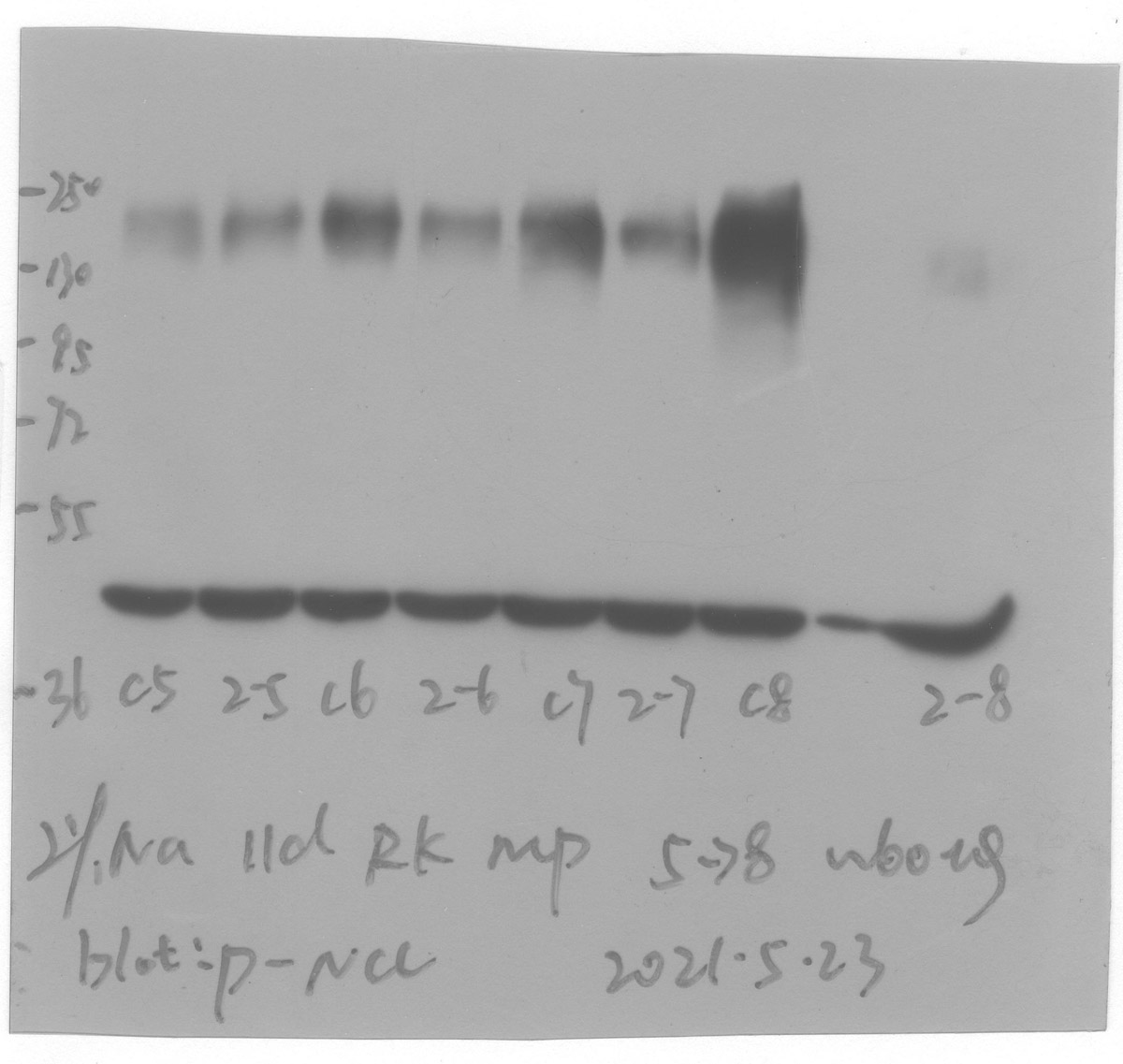

Supplement: Supplementary file 3 [file DataSheet2.ZIP › Small sized/Figure 9F-2% Na-p-NCC + Actin.jpg]

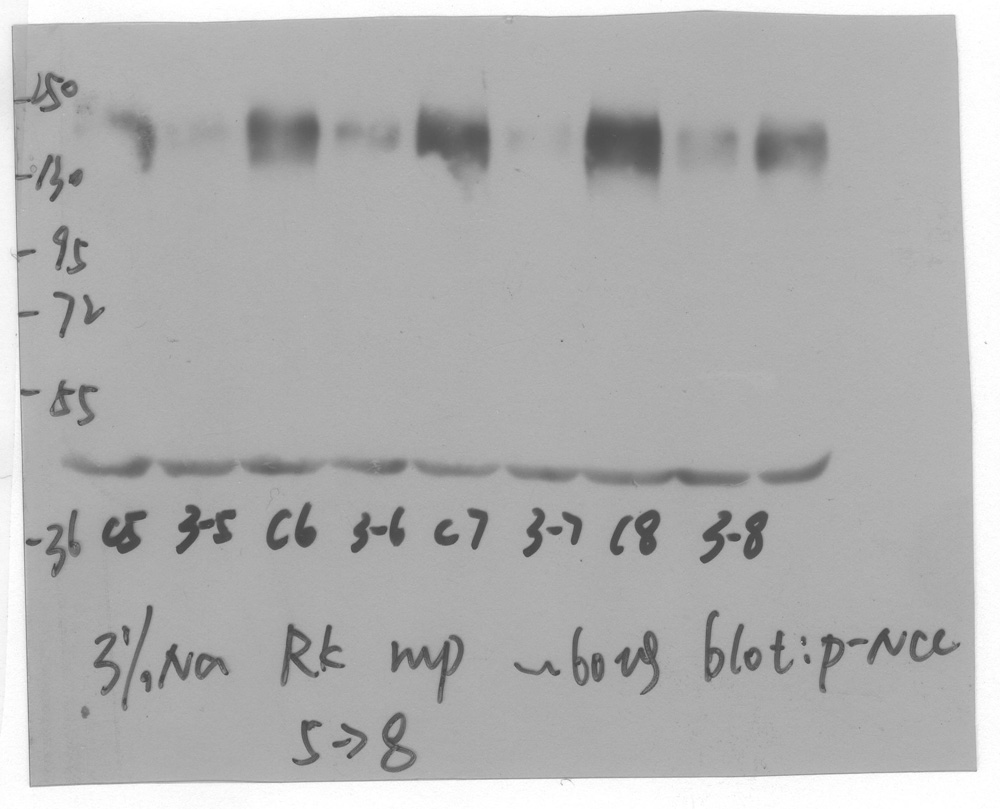

Supplement: Supplementary file 3 [file DataSheet2.ZIP › Small sized/Figure 9F-3% Na-p-NCC + Actin.jpg]
